# Innovative Arylimidazole‐Fused Phytovirucides via Carbene‐Catalyzed [3+4] Cycloaddition: Locking Viral Cell‐To‐Cell Movement by Out‐Competing Virus Capsid‐Host Interactions

**DOI:** 10.1002/advs.202309343

**Published:** 2024-03-13

**Authors:** Chunle Wei, Chunni Zhao, Jiao Li, Chunyi Li, Baoan Song, Runjiang Song

**Affiliations:** ^1^ National Key Laboratory of Green Pesticide Key Laboratory of Green Pesticide and Agricultural Bioengineering Ministry of Education Center for R&D of Fine Chemicals of Guizhou University Guiyang 550025 China

**Keywords:** antiviral activity, cell to cell movement, mechanism of action, N‐heterocyclic carbene, potato virus Y

## Abstract

The control of potato virus Y (PVY) induced crop failure is a challengeable issue in agricultural chemistry. Although many anti‐PVY agents are designed to focus on the functionally important coat protein (CP) of virus, how these drugs act on CP to inactivate viral pathogenicity, remains largely unknown. Herein, a PVY CP inhibitor **‐3j** (*S*) is disclosed, which is accessed by developing unusually efficient (up to 99% yield) and chemo‐selective (> 99:1 er in most cases) carbene‐catalyzed [3+4] cycloaddition reactions. Compound **‐3j** bears a unique arylimidazole‐fused diazepine skeleton and shows chirality‐preferred performance against PVY. In addition, **‐3j** (*S*) as a mediator allows ARG191 (R^191^) of CP to be identified as a key amino acid site responsible for intercellular movement of virions. R^191^ is further demonstrated to be critical for the interaction between PVY CP and the plant functional protein NtCPIP, enabling virions to cross plasmodesmata. This key step can be significantly inhibited through bonding with the **‐3j** (*S*) to further impair pathogenic behaviors involving systemic infection and particle assembly. The study reveals the in‐depth mechanism of action of antiviral agents targeting PVY CP, and contributes to new drug structures and synthetic strategies for PVY management.

## Graphical abstract

A highly efficient and selective carbene‐catalyzed [3+4] cycloaddition is developed in this study, which allows the one‐step synthesis of a series of optically‐enriched imidazo[1,2‐α][1,4]diazepins. Compound −**3j** (*S*) is screened out as a potential phytovirucide that locks cell‐to‐cell movement of potato virus Y by out‐competing virus capsid‐host interactions at the molecular level.

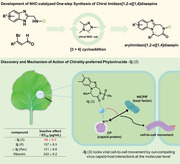

## 1 Introduction

Asymmetric catalytic synthesis is a powerful tool for designing and producing functional chiral molecules.^[^ ^1^ ^]^ Especially in the medical field, human drug discoveries enabled by asymmetric catalytic synthesis have received much attention with numerous achievements. As a remarkable instance, L‐dopa, a classic medicine used for the treatment of Parkinson's disease, has been successfully industrialized via asymmetric catalytic hydrogenation processes.^[^ ^2^ ^]^ When it comes to pesticide areas, however, (*S*)‐metolachlor is a rare success story that produces over 10 000 tons each year through asymmetric catalytic reactions as an efficient herbicide all over the world.^[^ ^3^ ^]^ Despite the unusual success of (*S*)‐metolachlor, asymmetric catalysis is much less used for designing new bioactive reagents for plant protection.^[^ ^4^ ^]^ In particular, the prospects of newly emerging catalytic strategies are barely explored to construct potentially potent antiviral and antibacterial molecules for plants.

**Figure 1:**
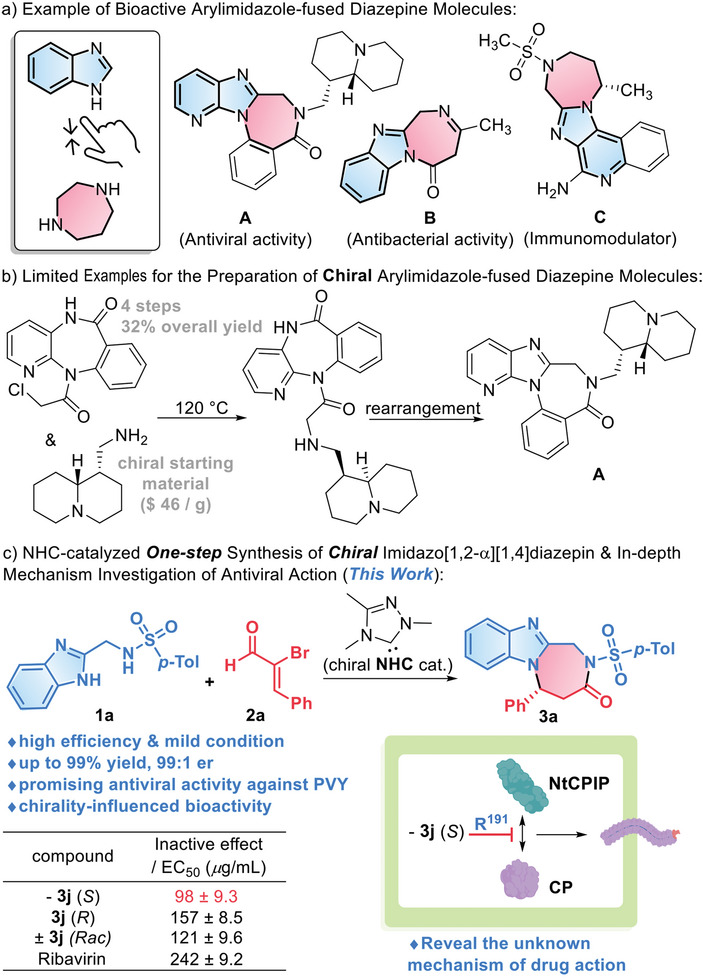
Biologically significant 1,4‐diazepin molecules and our catalytic approach for asymmetric construction of chiral diazepines.

Our long interests in plant protection ^[^ ^5^ ^]^ drive our attentions to molecules containing benzimidazoles and diazepine derivatives as key structural motifs (**Figure** **1**a).^[^ ^6^ ^]^ Indeed, arylimidazole‐fused diazepine derivatives possess versatile biological activities (Figure 1a).^[^ ^7^ ^]^ For example, the chiral pyridoimidazo[1,2‐α][1,4]diazepin **A** exhibits promising antiviral activities in the development of human medicines.^[^ ^8^ ^]^ The benzoimidazo[1,2‐α][1,4]diazepin **B** has been proven to be an efficient antifungal and antibacterial reagent.^[^ ^9^ ^]^ The chiral compound **C** containing an arylimidazo[1,2‐α][1,4]diazepin subunit has been used as an immunomodulator to treat viral and neoplastic diseases in various animals.^[^ ^10^ ^]^ These prior studies in the fields of human and animal medicines have encouraged our move to develop this kind of drug for plants. Unfortunately, there are few methods for asymmetric access to molecules that can properly fuse benzimidazoles and diazepine derivatives.^[^ ^11^ ^]^ The limited examples of starting with chiral substrates via a multiple‐step protocol (Figure 1b) cannot serve our purpose, especially when libraries of a relatively large number of molecules need to be prepared.

The coat proteins (CPs) of plant viruses are crucial for multiple stages of the viral life cycle, including virus particle assembly, protection of the viral genome, cell‐to‐cell movement in the host, and vector transmission.^[^ ^12^ ^]^ Consequently, strategically designing antiviral agents targeting CP has received widespread attention.^[^ ^13^ ^]^ However, the stories behind the excellent activity of many antiviral agents have rarely been explored, particularly how they act on CP and suppress viral pathogenic behavior. This may be attributed to the poor optical purity of these drugs leading to the identification of ineffective amino acid sites.

Herein, we present a carbene‐catalyzed [3 + 4] cycloaddition of aminomethylbenzimidazole **1a** to α‐bromoenal **2a**, which allows highly efficient access to a myriad of optically‐enriched arylimidazole‐fused diazepine derivatives (Figure 1c). Substrate **1a** contains three nitrogen atoms that are potentially reactive as nucleophiles. Under our catalytic conditions enabled by *N*‐heterocyclic carbene (NHC) organic catalysts,^[^ ^14^ ^]^ these nitrogen atoms undergo chemo‐selective reactions with the α‐bromoenal **2a** to eventually form arylimidazo[1,2‐α][1,4]diazepin **3a** (benzimidazole‐fused diazepine derivatives) in a nearly quantitative yield as essentially a single enantiomer (99:1 er). It is worth noting that *β*‐carbon amination of enals is very challenging in NHC catalysis, as documented by Chi and others.^[^ ^15^ ^]^ Our present study poses a significant advance in this direction. Additionally, our reaction is unusually chemo‐selective and places two nitrogen atoms on the enals selectively (diamination of enals). The exceptionally high yields and optical purities allow us to quickly prepare a sizable number of enantiomerically pure molecules for biostudies.

Several of our molecules showed excellent antiviral bioactivities that are better than commercial antiviral agent (Ribavirin). Moreover, chirality‐preferred performance in a few cases shows moderate while consistent and encouraging benefits, which motivates further investigation of the mechanistic reasons for this interesting phenomenon. Molecular docking combined with a suite of biological experiments revealed, for the first time, a brand‐new target site ARG191 (R^191^) of potato virus Y (PVY) CP with a unique mechanism of action. We provide evidence that our compound **−3j** (*S*) has the capacity to interact with the PVY CP protein by binding specifically to residue R^191^. This interaction effectively prevents virions from interacting with the host functional protein NtCPIP to cross plasmodesmata (PD), resulting in a failure to engage in intercellular movement. Furthermore, the disruption of viral movement has the potential to greatly diminish the occurrence of pathogenic behaviors, including systemic infection and particle assembly. This study deepens our understanding of the mechanism of action of drugs targeting CP, and provides available drugs and synthetic strategies for phytoviral control.

## 2 Results and Discussion

We chose 2‐aminomethylbenzimidazole **1a** and α‐bromoenal **2a** as the substrates since both of them are readily available and easily scalable when needed. The main results of the condition optimization for the reaction between **1a** and **2a** are summarized in **Table** **1**. To our delight, the indanol‐derived NHC catalyst **A** ^[^ ^16^ ^]^ bearing an *N*‐mesityl substituent gave target product **3a** an excellent yield and er value (Table 1, entry 1). Replacing the *N*‐mesityl group of the catalyst **A** with an *N*‐Ph group (to afford NHC catalyst **B** ^[^ ^17^ ^]^) resulted in drops on both the product yield and er value (entry 2). Further decreasing the electron density of the *N*‐substituent of the indanol‐derived NHC catalyst resulted in little formation of the desired product (e.g., entry 3, NHC catalyst **C** ^[^ ^18^ ^]^). The NHC catalyst **D** ^[^ ^19^ ^]^ also gave the diazepine product **3a** in a good yield, but the er value was only moderate (entry 4). Various organic or inorganic bases could also be used for this NHC‐catalyzed [3 + 4] annulation reaction, but either the product yields or er values were dropped in these cases (entries 5 to 7). Solvents other than THF gave the target product **3a** in relatively lower yields (entries 8 to 9). Finally, chiral diazepine **3a** was obtained in an almost quantitative yield with exceptional enantioselectivity when using a less amount of NHC catalyst **A** (entry 10). Further decreasing the NHC catalyst loading resulted in drops of the reaction yield (for details, see Table S1, Supporting Information).

**Table 1:** Optimization of reaction conditions.

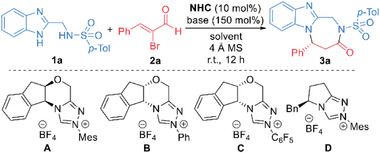
Entry^a)^	NHC	Base	Solvent	Yield [%]^b)^	er^c)^
1	**A**	K_2_CO_3_	THF	96	98:2
2	**B**	K_2_CO_3_	THF	69	81:19
3	**C**	K_2_CO_3_	THF	Trace	–
4	**D**	K_2_CO_3_	THF	76	85:15
5	**A**	Cs_2_CO_3_	THF	95	96:4
6	**A**	DBU	THF	54	98:2
7	**A**	Et_3_N	THF	86	99:1
8	**A**	K_2_CO_3_	acetone	78	99:1
9	**A**	K_2_CO_3_	CH_2_Cl_2_	82	99:1
10^d)^	**A**	K_2_CO_3_	THF	99	99:1

^a)^ Unless otherwise specified, the reactions were carried out with **2a** (0.1 mmol), **1a** (0.05 mmol), NHC (20 mol %), base (150 mol %), 4Å MS (80 mg), solvent (1 mL), at rt for 12 h;

^b)^ Isolated yield;

^c)^ Determined by chiral HPLC analysis (IA column, 1.0 mL mi^−1^n, hexans/iPrOH = 70/30);

^d)^ **2a** (0.2 mmol), **1a** (0.1 mmol), NHC (10 mol %), K_2_CO_3_ (200 mol %), 4Å MS (150 mg), THF (2 mL), rt, 12 h.

**Scheme 1:**
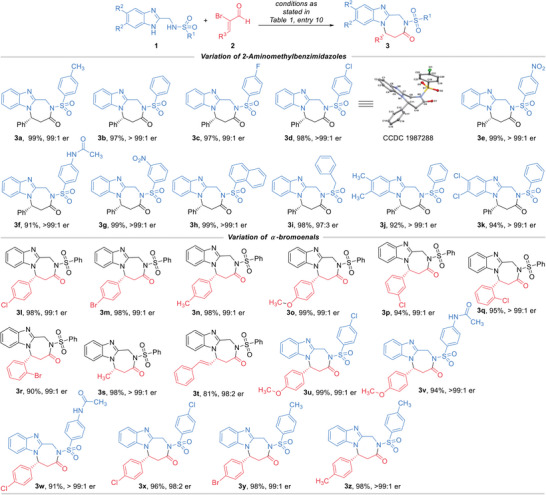
Scope of 2‐Aminomethyl‐benzimidazoles **1** and Enal Substrates **2**. Reaction conditions as stated in Table 1, entry 10. Yields are isolated yields after purification by column chromatography. Er values were determined via HPLC or UPLC on chiral stationary phase.

With the optimized reaction condition in hand, we next examined the reaction scope using both 2‐aminomethyl‐benzimidazoles **1** and enal substrates **2** containing different substituents and substitution patterns (**Scheme** **1**). Both electron‐donating and ‐withdrawing groups were well tolerated on each position of the phenyl rings in the sulphonamide moieties, with the corresponding diazepine products **3** affording in excellent yields and enantioselectivities (**3a** to **3h**). Moreover, the phenyl group of the sulphonamide fragment could be switched to a benzyl group without much erosion on the product yield or optical purity (**3i**). Noteworthily, it is possible to install substituents onto the phenyl rings of the benzoimidazole group, resulting in the formation of the desired products with high yields and enantiomeric purities (**3j** & **3k**). Enal substrates also exhibit tolerance toward diverse substitution patterns. For instance, substituents installed on each position of the phenyl ring of **2** could give the title products in excellent yields and enantioselectivities regardless of their electronic properties (**3l** to **3r**). Aliphatic enal substrates were also suitable substrates for this NHC‐catalyzed [3 + 4] reaction, with the corresponding diazepine products afforded in almost quantitative yields as single enantiomers (e.g., **3s**). The phenyl group on enal **2a** could also be replaced with a conjugated styrenyl group without erosion of product er value, although the yield was slightly dropped (**3t**). Note that, the reactions also worked extremely well when using substrates bearing different substituents on both 2‐aminomethyl‐benzimidazoles **1** and enals **2**, the title diazepine compounds were afforded in excellent yields and enantioselectivities (**3u** to **3z**).

**Scheme 2:**
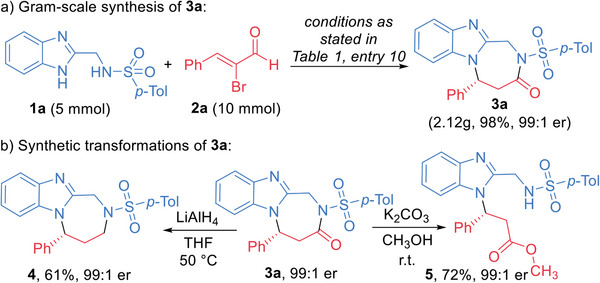
Gram‐scale synthesis and synthetic applications of **3a**.

This NHC‐catalyzed asymmetric [3 + 4] cycloaddition reaction can be executed at gram scale without obvious erosion on the compound yield and optical purity (**Scheme** **2**a). Chiral diazepine product **3a** can also be transformed into various functional molecules with retention of the optical purities (Scheme 2b). For example, the carbonyl group of the diazepine ring of 3a can be efficiently reduced by LiAlH_4_ to give the chiral product **4** in a 61% yield. The diazepine ring of **3a** can also be broken under weakly basic conditions through a trans‐esterification reaction and giving multi‐functional chiral product **5** in a good isolated yield. All the evidence unequivocally points that our catalytic tactic is highly effective, exhibiting selectivity and scalability, while our products demonstrate a high degree of convertibility. Therefore, it can be considered a powerful tool for accessing a diverse range of valuable molecules.

In order to assess the application of our methodology in the field of agrochemistry, the antiviral activities of the chiral diazepine products against PVY were systematically examined (**Tables** **2** and 3). The commercially available antiviral agent Ribavirin was used as the positive control. Several of our diazepine products exhibited encouraging antiviral activities against PVY. For instance, −**3j** (*S*) obtained from our approach showed better curative, protective and inactive effects than Ribavirin. The preliminary structure‐activity relationships based on the anti‐PVY activities proclaimed that the compound with high anti‐PVY activity occurred when one side of the sulfonamide sulfur atom was replaced by a phenyl group (**3b**>**3a**, **3c**–**3i**). Based on the antiviral activities of **3b**, the investigation of the substituents of benzimidazole revealed that it is beneficial for antiviral activity when the benzene ring is methyl‐substituted (**3j**>**3b**). In addition, the examination of the substituents of enal substrates **2** based on the antiviral activities of **3b** revealed that the type of α‐bromoenal had a significant impact on antiviral activities, but their anti‐PVY activities did not exceed that of compound **3j**. It is also worth noting that the (*S*)‐enantiomers of the chiral products have generally displayed superior biological activities against PVY compared to their corresponding (*R*)‐enantiomers and racemic mixtures (Table 2). Meanwhile, the EC_50_ values of the chiral antiviral compounds −**3j** (*S*) and others against PVY also showed better efficacies than the commercial Ribavirin (Table **3**) (for details, see Table S2, Supporting Information). The bio‐evaluations yielded exciting findings that demonstrate the potential of our strategy for pesticide research and development. These results serve as motivation to further investigate the underlying mechanism reaction of −**3j** (*S*) against PVY.

**Table 2:** The chiral products anti‐PVY in vivo at 500 µg mL^−1^.

Compound^a^ ^)^	Curative Effect [%]	Protective Effect [%]	Inactive Effect [%]
**3j** (*R*)	55.3 ± 4.5	57.3 ± 4.6	80.5 ± 4.2
− **3j** (*S*)	61.0 ± 3.8	63.2 ± 4.0	85.4 ± 4.4
± **3j** (*rac*)	54.0 ± 2.1	53.4 ± 3.2	79.9 ± 3.7
**3l** (*R*)	52.2 ± 1.9	55.4 ± 4.1	71.5 ± 4.4
− **3l** (*S*)	55.4 ± 3.7	58.2 ± 1.7	78.4 ± 1.8
± **3l** (*rac*)	52.6 ± 3.4	53.6 ± 4.3	75.4 ± 2.2
**3n** (*R*)	51.3 ± 3.3	59.6 ± 3.5	78.5 ± 3.7
− **3n** (*S*)	60.2 ± 4.3	63.9 ± 4.1	83.4 ± 4.6
± **3n** (*rac*)	53.0 ± 4.6	53.2 ± 2.1	76.2 ± 3.9
**3r** (*R*)	53.5 ± 2.4	57.7 ± 4.2	70.3 ± 4.1
− **3r** (*S*)	57.1 ± 3.2	55.1 ± 3.4	79.7 ± 1.1
± **3r** (*rac*)	51.2 ± 1.6	54.2 ± 4.2	78.8 ± 3.9
**3z** (*R*)	53.3 ± 2.2	57.2 ± 3.3	71.4 ± 4.8
− **3z** (*S*)	58.4 ± 2.9	59.5 ± 3.0	73.0 ± 2.9
± **3z** (*rac*)	53.8 ± 3.5	56.7 ± 3.0	74.8 ± 3.2
Ribavirin	45.5 ± 2.4	48.2 ± 1.9	63.5 ± 2.1

^a)^All data were average data of three replicates. Ribavirin was used as the positive control.

**Table 3:** EC_50_ values of the chiral products anti‐PVY in vivo (µg mL^−1^).

Compound^a)^	Curative Effect	Protective Effect	Inactive Effect
− **3j** (*S*)	239 ± 7.9	198 ± 8.9	98 ± 9.3
**3j** (*R*)	425 ± 8.4	386 ± 9.1	157 ± 8.5
± **3j** (*rac*)	461 ± 7.3	472 ± 8.6	121 ± 9.6
−**3n** (*S*)	262 ± 8.1	205 ± 9.7	128 ± 7.2
Ribavirin	650 ± 8.3	627 ± 7.8	242 ± 9.2

^a)^ All data were average data of three replicates. Ribavirin was used as the positive control.

At present, two main strategies exist for addressing plant viral infections. One primary approach involves the stimulation of the host immune system to combat viral invasion, a process that can be promoted by specific substances referred to as plant immune inducers.^[^ ^20^ ^]^ The implementation of antiviral drugs represents another method for combating viral infections. These drugs exert their effects by targeting either the genetic material or essential proteins of the virus, thereby impeding its pathogenic behavior. This approach is widely regarded as being relatively uncomplicated and exhibiting a more rapid onset of action.^[^ ^21^ ^]^

CP is a multifunctional protein in PVY that contributes significantly to various aspects of viral infection, such as cell‐to‐cell movement, virus replication, and other critical processes.^[^ ^22^ ^]^ PVY CP has thus been considered as a prospective target for the development of antiviral strategies. Unfortunately, this remains stagnant as the exact target sites of action of antiviral drugs have not yet been elucidated at present. Given the outstanding inactivated performance of our compound −**3j** (*S*) on PVY, a hypothesis was proposed that our products may affect virus infection by inhibiting the normal functions of PVY CP.

**Figure 2:**
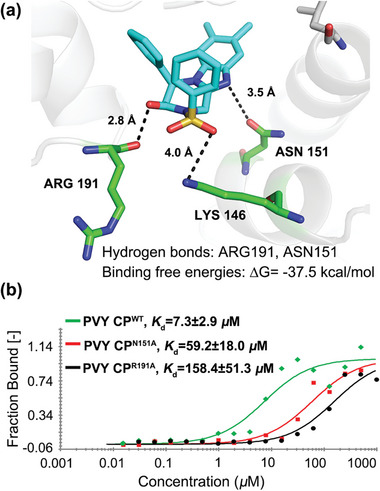
a) Molecule docking results of compound −**3j** (*S*). b) Microscale thermophoresis assay was used to detect the binding affinity of compound −**3j** (*S*) to wild‐type and mutated PVY CP.

To verify our hypothesis, molecular docking was employed to understand the potential binding sites of our products with CP. The docking pattern of compound −**3j** (*S*) with the crystal structure of PVY CP (PDB ID: 6HXZ) is disclosed in **Figure** 2a. The results showed that compound −**3j** (*S*) formed strong hydrogen bonds with R^191^ and ASN151 (N^151^). In addition, the binding stability of compound −**3j** (*S*) to the receptor protein was evaluated by surveying the root mean square deviation values of the initial positions of the atoms via molecular dynamics simulation (MDS) (Figure S1, Supporting Information). The binding free energy of compound −**3j** (*S*) and PVY CP molecular dynamics orbital was calculated by the molecular mechanics Poisson–Boltzmann surface area method to be −37.55 kcal mol^−1^. These experiments initially confirmed that our compound −**3j** (*S*) binds to CP via hydrogen bonding and identified two potential binding sites (R^191^ or N^151)^.

Microscale thermophoresis (MST) is a powerful tool for studying the interaction intensity between small molecules and ligands.^[^ ^23^ ^]^ To further verify that R^191^ or N^151^ is the key amino acid site for compound −**3j** (*S*) to act on PVY CP, we constructed wild‐type PVY CP (PVY CP^WT^) and mutated PVY CP (PVY CP^N151A^, and PVY CP^R191A^) prokaryotic expression vectors and expressed them in *Escherichia coli* to obtain the corresponding proteins that were subsequently implemented to MST analysis. The results showed that compound −**3j** (*S*) had a powerful binding affinity to PVY CP^WT^ with a dissociation constant (*K*_d_) of 7.3 µm, which is stronger than that of the mutated PVY CP^N151A^ (59.2 µm) and PVY CP^R191A^ (158.4 µm) (Figure 2b). These findings corroborated the results obtained from molecular docking (Figure 2a) and MDS (Figure S1, Supporting Information) in an in vitro setting.

**Figure 3:**
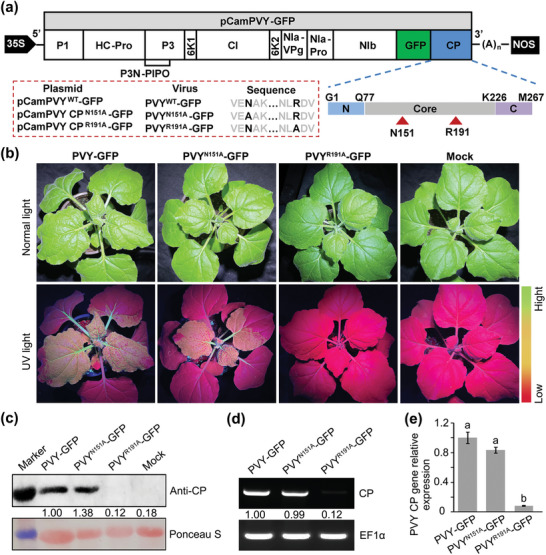
Residue R^191^, where compound −**3j** (*S*) binds to PVY CP, is critical for PVY systemic infection. a) Schematic diagram of the pCamPVY‐GFP genomic structure. N^151^ and R^191^ indicated by red arrowheads are located in the core region of PVY CP. The site‐directed mutagenized and wild‐type plasmids, viruses, and sequences are displayed in the red‐lined box. b) Symptoms (upper panel) and green fluorescence (lower panel) under UV light of *N. benthamiana* infiltrated by wild‐type and mutated PVY. c–e) The accumulation levels of PVY CP in the systemically infected leaves of the wild‐type and mutated PVY‐infected *N. benthamiana* plants were analyzed at 7 days postagroinfiltration (dpai) by Western blot, reverse transcription polymerase chain reaction (RT‐PCR), and quantitative RT‐PCR (RT‐qPCR), respectively. EF1*α* of the internal control gene was used during RT‐qPCR and RT‐PCR. Staining of RuBisCO with Ponceau S was used as a sample loading control. The data are shown as means ± *SD* from three biological replicates per treatment. Different letters mean statistically significant differences (*p* <0.05, one‐way analysis of variance).

To further verify in vivo that R^191^ or N^151^ in the PVY CP is possible binding target site for compound −**3j** (*S*) and its mediated functions, we mutagenized the R^191^ and N^151^ residues in the CP of pCamPVY‐GFP, a PVY infectious clone carrying the GFP gene ^[^ ^24^ ^]^, with an alanine (A) residue to acquire the plasmids pCamPVY CP^N151A^‐GFP and pCamPVY CP^R191A^‐GFP, respectively (**Figure** **3**a). Agrobacterium cultures carrying the plasmids pCamPVY‐GFP, pCamPVY CP^N151A^‐GFP, or pCamPVY CP^R191A^‐GFP were individually infiltrated into *N. benthamiana*. PVY‐GFP‐ and PVY^N151A^‐GFP‐infected *N. benthamiana* plants displayed crinkling and mosaic symptoms on the systemically infected leaves. In contrast, the mutant PVY^R191A^‐GFP showed barely noticeable foliar symptoms at 7 days postagroinfiltration (dpai) (Figure 3b, upper panel). Meanwhile, the plants infected with PVY‐GFP and PVY^N151A^‐GFP displayed strong green fluorescence in young leaves under UV light (Figure 3b, lower panel). The results of the Western blot, reverse transcription polymerase chain reaction (RT‐PCR), quantitative RT‐PCR (RT‐qPCR), and enzyme‐linked immunosorbent assay exhibited that PVY CP and GFP accumulated in the systemic leaves of PVY‐GFP‐ and PVY^N151A^‐GFP‐infected *N. benthamiana*, but not in the systemic leaves of PVY^R191A^‐GFP‐infected *N. benthamiana* plants (Figure 3c–e; Figure S2a,b, Supporting Information). Taken together, these findings suggest that R^191^ rather than N^151^ in the CP is essential for PVY systemic infection in *N. benthamiana* plants. In the context of potyvirus infection, it is important to consider the replication and movement of viruses as the primary processes involved. Consequently, it is plausible that the presence of R^191^ or N^151^ in PVY CP is directly associated with one or both of these processes.

**Figure 4:**
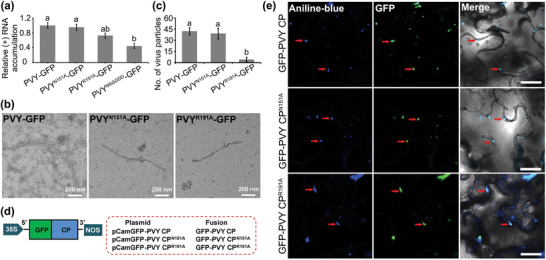
a) The accumulation of PVY (+) RNA in various infiltrated leaf samples was determined through RT‐qPCR using specific primers at 60 hours postagroinfiltration (hpai). The expression of EF1α was used as an internal control. b) Particles of PVY‐GFP, PVY^N151A^‐GFP, and PVY^R191A^‐GFP under transmission electron microscope. c) The numbers of PVY particles in fields of 70 µm^2^. The data are shown as means ± *SD* from five fields per treatment. Different letters mean statistically significant differences (*p* <0.05, one‐way analysis of variance). d) A schematic diagram showing plasmids expressing a GFP‐PVY CP, GFP‐PVY CP^N151A^, or GFP‐PVY CP^R191A^ fusion. e) Effects of R^191^ and N^151^ on PVY CP subcellular localization. Pictures were photographed at 72 hpai under confocal microscope.

The process of potyvirus replication holds significant importance in the context of plant infection.^[^ ^25^ ^]^ In order to ascertain the potential involvement of N^151^ or R^191^ of CP in PVY replication, we conducted an analysis of the accumulation levels of PVY plus‐strand (+) RNA in the infiltrated leaf tissues using RT‐qPCR. The findings showed that the PVY CP^R191A^‐GFP‐inoculated leaves exhibited slightly lower levels of (+) RNA accumulation compared to the PVY‐GFP and PVY CP^N151A^‐GFP‐inoculated leaf tissues, while higher levels than those in the PVY^NIbΔGDD^‐GFP‐inoculated (produce replication‐deficient mutant as control) ^[^ ^26^ ^]^ leaf tissues (**Figure** **4**a), indicating that R^191^ has moderate effect on PVY replication.

The assembly of potyviral particles plays a critical role in facilitating viral infection.^[^ ^27^ ^]^ To examine the impact of the R^191^ or N^151^ sites of CP on this process, we purified PVY‐GFP, PVY^N151A^‐GFP, and PVY^R191A^‐GFP‐infected *N. benthamiana* leaves at 5 dpai, respectively. Transmission electron microscopy was used to visualize viral particles in the PVY^N151A^‐GFP, PVY^R191A^‐GFP, and PVY‐GFP groups. No significant differences were found in the morphology of virions among the three groups. This similarity suggests that mutations in PVY did not alter the ability of CP to encapsidate PVY genome (Figure 4b). However, the number of viral particles was significantly reduced in PVY^R191A^‐GFP compared to PVY‐GFP and PVY^N151A^‐GFP‐infected samples (Figure 4c). On this basis, the mutant R^191^ did not directly mediate virion formation by blocking CP encapsulation. On the other hand, it is well known that the intercellular movement of viruses plays a crucial role in facilitating virus system infection and particle assembly. Hence, it is reasonable to speculate that limited virion accumulation may be primarily attributed to restricted intercellular mobility ^[^ ^28^ ^]^ caused by amino acid mutations.

It is important to note that a comprehensive classification system for PVY was developed based on the biological, serological, and molecular characteristics of PVY isolates. Within this classification, certain strains have been identified that possess the ability to induce vein necrosis in *N. tabacum* cv. K326 plants.^[^ ^29^ ^]^ This adverse symptom is largely due to intercellular transmission of the virus within the host.^[^ ^30^ ^]^ To investigate whether the sites N^151^ or R^191^ of CP can affect the symptom of vein necrosis, we inoculated the *N. tabacum* cv. K326 plants with PVY‐GFP, PVY^N151A^‐GFP or PVY^R191A^‐GFP, respectively. The systemic leaves of PVY‐GFP and PVY^N151A^‐GFP‐infected *N. tabacum* cv. K326 plants showed obvious symptoms of vein necrosis; however, no vein necrosis was observed in the PVY^R191A^‐GFP‐infected plants at 21 dpai (Figure S3, Supporting Information). The result of trypan blue staining confirmed the presence of vein necrosis in systemic leaves of *N. tabacum* cv. K326 plants of PVY‐GFP and PVY^N151A^‐GFP groups, whereas vein necrosis symptoms were absent in PVY^R191A^‐GFP‐infected plants. These results indicated that R^191^ of CP is crucial for the symptom of vein necrosis induced by PVY‐GFP in *N. tabacum* cv. K326 plants.

Potyviral cell‐to‐cell movement through PD is a key process for systemic infection within plants.^[^ ^28c^ ^]^ The localization of virions in close proximity to PD is essential for facilitating their transmembrane transport. Thus, to examine the effects of R^191^ and N^151^ on PVY CP subcellular localization, we individually infiltrated the leaves of *N. benthamiana* with agrobacterium cultures carrying the plasmids pCamGFP‐PVY CP, pCamGFP‐PVY CP^N151A^, and pCamGFP‐PVY CP^R191A^ (Figure 4d). Confocal microscopy analysis of infiltrated leaves treated with aniline blue (callose staining, showing PD localization) ^[^ ^31^ ^]^ for 15 min at 72 h postagroinfiltration (hpai) showed that GFP‐PVY CP, GFP‐PVY CP^N151A^, and GFP‐PVY CP^R191A^ displayed aniline blue‐stained callose present co‐localized with GFP‐PVY in the edge of the cell (Figure 4e). These observed phenomena provide evidence that GFP‐PVY CP, GFP‐PVY CP^N151A^, and GFP‐PVY CP^R191A^ retain their normal functions in PD.

**Figure 5:**
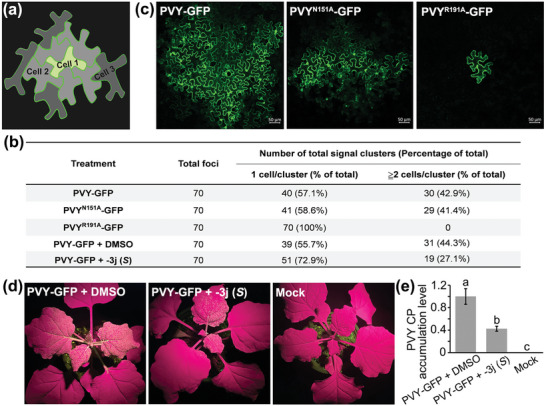
a) Scheme of the cell‐to‐cell movement. b) Efficiency of intercellular movement of wild‐type and mutated PVY‐infected *N. benthamiana* plants and compound −**3j** (*S*) inhibiting cell‐to‐cell movement of PVY‐GFP in *N. benthamiana* at 48 hpai. −**3j** (*S*) was used at 25 µg mL^−1^. c) Cell‐to‐cell movement of wild‐type and mutated PVY‐infected *N. benthamiana* plants at 72 hpai. d) Effects of compound −**3j** (*S*) on the systemic infection of PVY‐GFP in *N. benthamiana*. The *N. benthamiana* infiltrated with PVY‐GFP treated by control, 500 µg mL^−1^ −**3j** (*S*) at 6 dpai. e) The accumulation levels of PVY CP in the systemically infected leaves of PVY‐GFP‐infected *N. benthamiana* plants were analyzed at 6 dpai by RT‐qPCR. EF1*α* of the internal control gene was used. The data are shown as means ± *SD* from three biological replicates per treatment. Different letters mean statistically significant differences (*p* <0.05, one‐way analysis of variance).

The role of N^151^ or R^191^ in PVY intercellular movement was further explored by infiltration of agrobacterium cultures harboring the plasmids pCamPVY‐GFP, pCamPVY CP^N151A^‐GFP, or pCamPVY CP^R191A^‐GFP into *N. benthamiana* leaves. Approximately 42.9 and 41.4% of the foci showed multicellular fluorescence from PVY‐GFP and PVY^N151A^‐GFP leaf tissues, respectively, in contrast to the single cells (0%) of such foci observed in PVY^R191A^‐GFP‐treated leaves at 48 hpai (**Figure** **5**b). When extended to 72 hpai, GFP green fluorescence from PVY‐GFP or PVY^N151A^‐GFP group appeared in clusters of multiple cells, whereas green fluorescence produced by PVY^R191A^‐GFP group was also confined in single cells (Figure 5c), Therefore, it can be concluded that R^191^ is crucial for PVY to carry out cell‐to‐cell movement in *N. benthamiana* plants.

To conduct a more comprehensive investigation of the impact of compound −**3j** (*S*) on the intracellular transportation of PVY‐GFP, negative control experiments were performed using DMSO. As shown in Figure 5b, ≈44.3% of the foci showed multicellular fluorescence in PVY‐GFP + DMSO‐treated control leaf tissues, in contrast to the much lower percentage (≈27.1%) of such foci observed in the −**3j** (*S*)‐treated leaves. In addition, Agrobacterium cultures expressing the pCamPVY‐GFP plasmid were infiltrated into the leaves of *N. benthamiana* after treatment with DMSO or compound −**3j** (*S*) for 24 h. The PVY‐GFP + DMSO‐treated *N. benthamiana* filled with green fluorescence in the upper stems and systemic leaves at 6 dpai. Conversely, green fluorescence was significantly reduced in the PVY‐GFP + compound −**3j** (*S*) group (Figure 5d). RT‐qPCR results (Figure 5e) exhibited that the PVY CP gene accumulated significantly in the systemic leaves of PVY‐GFP + DMSO‐treated *N. benthamiana*, while it was obviously reduced in the PVY‐GFP + compound −**3j** (*S*) group, which is consistent with the results observed in Figure 5d. These results further demonstrated that compound −**3j** (*S*) can inhibit the cell‐to‐cell movement and systemic infection with PVY‐GFP in *N. benthamiana*.

**Figure 6:**
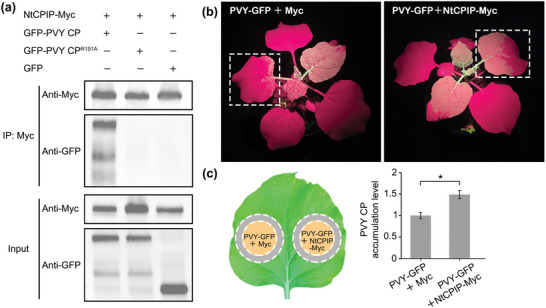
a) Interaction between NtCPIP and PVY CP or PVY CP^R191A^. GFP was used as a negative control. Immunoprecipitation was performed with an anti‐Myc antibody and immunoblots were probed with anti‐GFP or anti‐Myc antibodies. b) GFP fluorescence in PVY‐GFP‐infected *N. benthamiana* plants transiently expressing NtCPIP‐Myc or Myc. The plants were photographed at 7 days post PVY‐GFP inoculation. c) RT‐qPCR analysis of viral intercellular movement in *N. benthamiana* leaf tissues surrounding the areas co‐inoculated with PVY‐GFP and NtCPIP‐Myc or Myc at 72 hpai. Grey zones shown in the leaf diagram were harvested for assays. The data are shown as means ± *SD* from three biological replicates per treatment. Statistical significance was tested with Student's *t*‐test. (^*^ *p* <0.05).

The effective infection of viruses on plants is inseparable from the recruitment of host factors.^[^ ^32^ ^]^ The properties of host factors involved in the cell‐to‐cell trafficking of potyviruses and their interaction with CP are gradually being revealed.^[^ ^33^ ^]^ Wherein DnaJ‐like proteins from tobacco, named NtCPIPs, are mainly involved in the intercellular movement of viruses and lead to the effective diffusion of PVY in host plants after interacting with CP. Meanwhile, mutations in the core region of CP abolish its interactions with NtCPIPs.^[^ ^34^ ^]^ Taking inspiration from these references, we speculated that the blocked intercellular movement of PVY^R191A^‐GFP may be related to the host chaperones NtCPIP. To verify our hypothesis, we performed co‐immunoprecipitation (CO‐IP) assays on *N. benthamiana* using a Myc‐tagged NtCPIP construct and GFP‐PVY CP or GFP‐PVY CP^R191A^. Fortunately, GFP‐PVY CP was associated with NtCPIP *in planta*, whereas no association between the mutant GFP‐PVY CP^R191A^ and NtCPIP was detected (**Figure** **6**a). Biological functional studies showed that the transient overexpression of NtCPIP promoted PVY‐GFP infection (Figure 6b). Furthermore, RT‐qPCR analysis of viral intercellular movement in *N. benthamiana* leaf tissues surrounding areas co‐inoculated with PVY‐GFP and NtCPIP‐Myc or Myc indicated that the presence of NtCPIP promoted PVY‐GFP movement and accumulation (Figure 6c). These results support the conjecture that the obstruction of cell‐to‐cell movement of PVY^R191A^‐GFP may be due to the interruption of the interaction between PVY CP^R191A^ and NtCPIP, and that NtCPIP is required for the effective intercellular trafficking of PVY.

## 3 Conclusion

**Figure 7:**
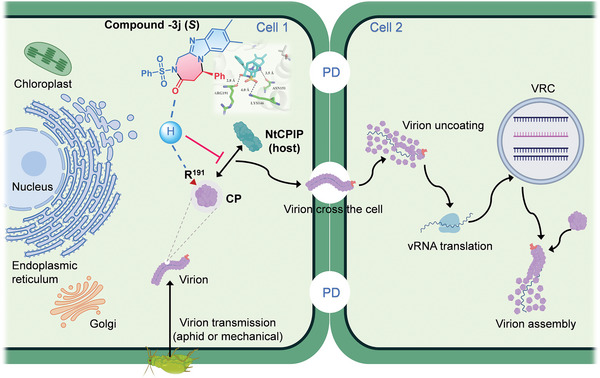
Proposed model of the mechanism of action for compound −**3j** (*S*) inhibiting PVY infection according to our observations and previous studies. Compound −**3j** (*S*) acts on the R^191^ of PVY CP site through hydrogen bonding, resulting in the loss of the function of R^191^ responsible for PVY intercellular movement. And the phenomenon of blocked cell‐to‐cell movement may be caused by disruption of the interaction between PVY CP and NtCPIP.

In summary, we developed an NHC‐catalyzed enantioselective [3+4] reaction to fuse benzimidazoles with diazepine derivatives in exceptionally high yields and optical purities. 2‐Aminomethylbenzimidazoles and α‐bromoenals are involved as readily available substrates in this protocol. Our products can tolerate various substitution patterns and show promising antiviral bioactivities against PVY that are better than commercial antiviral agent. Compound −**3j** (*S*), screened from 75 products, showed promise as a candidate phytovirucide. Molecular docking combined with MDS and MST cooperatively identified and validated the site R^191^, where the drug acts on CP in vitro. A series of in vivo experiments suggested that R^191^ is of critical responsibility for the interaction between PVY CP and the functional protein NtCPIP to perform the cell‐to‐cell movement of PVY virions. Inhibition of this process by our drug **−3j** (*S*) significantly weakened the viral pathogenicity (**Figure** **7**).

Overall, this study enhances our comprehension of the mechanism by which drugs that target CP, offering insights into novel anti‐viral agents and synthetic approaches for the management of phytoviral infections.

## 4 Experimental Section

Preparation of substrates (see Page no. SP4, Supporting Information), optimization of reaction conditions (see Page no. SP6, Supporting Information), gram‐scale synthesis of product **3a** (see Page no. SP8, Supporting Information), synthetic applications of product **3a** (see Page no. SP8, Supporting Information), antiviral bioassay (see Page no. SP11, Supporting Information), molecular docking (see Page no. SP13, Supporting Information), MST assay (see Page no. SP13, Supporting Information), plant growth (see Page no. SP13, Supporting Information), virus inoculation (see Page no. SP13, Supporting Information), protein transient expression (see Page no. SP13, Supporting Information), RNA extraction (see Page no. SP13, Supporting Information), RT‐PCR (see Page no. SP13, Supporting Information), RT‐qPCR (see Page no. SP13, Supporting Information), Western blot assay (see Page no. SP14, Supporting Information), trypan blue staining (see Page no. SP14, Supporting Information), virus particle purification (see Pahe no. SP14, Supporting Information), confocal microscopy and GFP imaging (see Page no. SP14, Supporting Information), molecular dynamics simulation (see Page no. SP15, Supporting Information), and CO‐IP (see Page no. SP15, Supporting Information) see Supporting Information.

### General Synthesis Procedure for Product 3

In a 10 mL Schlenk tube with a stir bar, 2‐aminomethyl‐benzimidazoles **1** (0.1 mmol, 1.0 equiv), 2‐bromoenals **2** (0.2 mmol, 2.0 equiv), K_2_CO_3_ (0.2 mmol, 200 mol %, 28 mg), chiral NHC pre‐catalyst A (0.01 mmol, 10 mol %, 4.2 mg) and 4Å molecular sieve (150 mg) were added. Freshly distilled dry THF (2 mL) was added (in case of liquid 2‐bromoenal, it was added in this step) using a syringe. The mixture was monitored using TLC plates at room temperature until the reaction was complete (generally 12–24 h). The mixture was concentrated and purified by silica gel column chromatography using hexanes/EtOAc (1:1) as the eluent to obtain product **3**.

### Plasmid Construction and Site‐Directed Mutagenesis

The GFP gene‐containing pCamPVY‐GFP (GenBank: MN381731) plasmid was constructed.^[^ ^24^ ^]^ The coding sequence of PVY CP was PCR‐amplified from pCamPVY‐GFP and inserted into a vector (pCambia0390 containing a 35S promoter and the GFP gene) to produce pCamGFP‐PVY CP. To express PVY CP in *Escherichia coli*, the PVY CP sequence was cloned into the pET‐28a (+) vector to generate pET‐PVY CP. Substitution of the codons for R^191^ or N^151^ in pCamPVY‐GFP or pET‐PVY CP was conducted using site‐directed mutagenesis, as previously described ^[^ ^35^ ^]^, to individually acquire pCamPVY^R191A^‐GFP, pCamPVY^N151A^‐GFP, pET‐PVY CP^R191A^, and pET‐PVY CP^N151A^. All the constructed plasmids were sequenced. Primers used in this study are listed in Table S3 (Supporting Information).

### Statistical Analysis

All experiments were conducted with three independent biological replicates per treatment. The sample size (*n*) for each measurement was determined in triplicate. The data are shown as means ± SD. Data were statistically analyzed by one‐way determine (ANOVA) or Student's *t*‐test using SPSS. Asterisks or different letters indicate statistically significant differences (*p* <0.05).

## Conflict of Interest

The authors declare no conflict of interest.

## Supporting information

Supporting Information

## Data Availability

The data that support the findings of this study are available in the supplementary material of this article.
