# Supplementary material for: Innovative Arylimidazole‐Fused Phytovirucides via Carbene‐Catalyzed [3+4] Cycloaddition: Locking Viral Cell‐To‐Cell Movement by Out‐Competing Virus Capsid‐Host Interactions
Source: Adv Sci (Weinh). 2024 Mar 13;11(19):2309343. doi: 10.1002/advs.202309343 (PMC11109656; doi:10.1002/advs.202309343)
Supplement: Supplementary file 1 — Supporting Information [file ADVS-11-2309343-s001.pdf]

ADVANCED SCIENCE
Open Access

Supporting Information

for *Adv. Sci.*, DOI 10.1002/advs.202309343

Innovative Arylimidazole-Fused Phytovirucides via Carbene-Catalyzed [3+4] Cycloaddition: Locking Viral Cell-To-Cell Movement by Out-Competing Virus Capsid-Host Interactions

*Chunle Wei, Chunni Zhao, Jiao Li, Chunyi Li, Baoan Song and Runjiang Song**

Supporting Information

Innovative Arylimidazole-Fused Phytovirucides via Carbene-catalyzed [3+4] Cycloaddition: Locking Viral Cell-to-Cell Movement by Out-competing Virus Capsid-Host Interactions

*Chunle Wei, Chunni Zhao, Jiao Li, Chunyi Li, Baoan Song, and Runjiang Song**

National Key Laboratory of Green Pesticide, Key Laboratory of Green Pesticide and Agricultural Bioengineering, Ministry of Education, Center for R & D of Fine Chemicals of Guizhou University, Guiyang 550025, China.

Contents

I. General information... 3

II. Preparation of substrates... 4

III. Reaction condition optimization.. 6

IV. General procedure for the catalytic reactions.. 8

V. X-ray crystallography of product **3d**.. 10

VI. Antiviral Bioassay... 11

VII. Mechanism of action... 13

VIII. Characterization of products...19

IX. NMR spectra of products 34

X. HPLC spectra of products 63

I. General information

Commercially available materials purchased from Aladdin or J&K were used as received. THF was distilled over sodium. Unless otherwise specified, all reactions were carried out under an atmosphere of nitrogen in 10 mL dry Schlenk tube. Proton nuclear magnetic resonance (^{1}H NMR) spectra were recorded on a Bruker (400 MHz) spectrometer or on a JEOL-ECX-500 (500 MHz) spectrometer. Chemical shifts were recorded in parts per million (ppm, δ) relative to tetramethylsilane (δ 0.00) or the corresponding deuterium solvent. ^{1}H NMR splitting patterns are designated as singlet (s), doublet (d), triplet (t), quartet (q), dd (doublet of doublets); m (multiplets), and etc. All first-order splitting patterns were assigned on the basis of the appearance of the multiplet. Splitting patterns that could not be easily interpreted are designated as multiplet (m) or broad (br). Carbon nuclear magnetic resonance (^{13}C NMR) spectra were recorded on a Bruker (101 MHz) spectrometer or on a JEOL-ECX-500 (126 MHz) spectrometer. Fluorine (^{19}F) nuclear magnetic resonance (^{19}F NMR) spectra were recorded on a Bruker (376 MHz) spectrometer or on a JEOL-ECX-500 (471 MHz) spectrometer. The melting points (m.p.) of the title compounds were determined when left untouched on an XT-4-MP apparatus from Beijing Tech. Instrument Co. (Beijing, China). High resolution mass spectral analysis (HRMS) was performed on a quadrupole/electrostatic field orbitrap mass spectrometer. Absolute configuration of the products was determined by X-ray crystallography. The determination of enantiomeric excess was performed via chiral HPLC analysis using Shimadzu LC-20AD HPLC workstation. Optical rotations were measured using a 1 mL cell with a 1 dm path length on a Jasco P-1030 polarimeter and are reported as follows: $[\alpha]^{rt}_{D}$ (c in g per 100 mL solvent). Analytical thin-layer chromatography (TLC) was carried out on Merck 60 F254 pre-coated silica gel plate (0.2 mm thickness). Visualization was performed using a UV lamp.

II. Preparation of substrates

2-aminomethylbenzimidazole were synthesized from 1, 2-diaminobenzene by the combination of slightly modified literature procedures.

Method 1 [1]

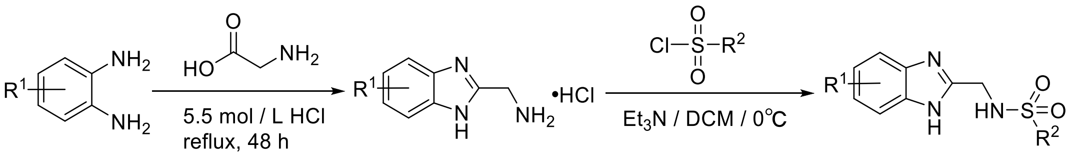

1, 2-diaminobenzene (55 mmol) was added to a 100 mL reaction flask containing 60 mL of 5.5 mol/L HCl, and refluxed at 100 ℃ for 1 h. Glycine (110mmol) was added and the mixture was refluxed for 48 hours. After standing overnight, a large amount of light green solid was precipitated, filtered, and the filter cake was washed with a small amount of ice ethanol, and dried to obtain white solid.

A solution of (1*H*-Benzoimidazol-2-yl) methylamine hydrochloride (25 mmol) in dichloromethane (30 mL) was treated with Tosyl chloride (25 mmol) and triethylamine (27.5 mmol). The resultant mixture was stirred at ice bath for 1 h. A saturated aqueous solution of sodium bicarbonate (60 mL) was added. The organic layers dried over sodium sulfate, and concentrated in vacuo. Recrystallize with dichloromethane to get white solid.

Method 2 [2]

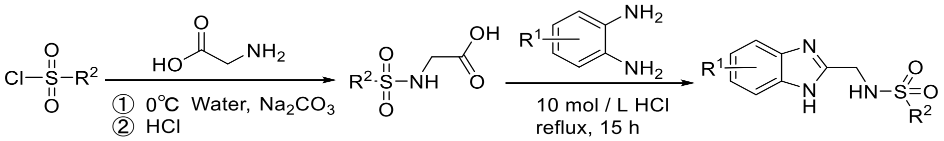

Sodium carbonate (24 mmol) was added to a solution of glycine (20 mmol) in water (25 mL) with continuous stirring until all the solutes dissolved at ice bath. Tosyl chloride (24 mmol) was added in four portions over a period of 1 h. The mixture was further stirred at room temperature for 5 h. The mixture was acidified using 20% aqueous hydrochloric acid to pH 2. The crystals was filtered and washed with pH 2.2 buffer, and dried to obtain white solid.

1, 2-diaminobenzene (5 mmol) was added to 25 mL of 10 mol/L HCl, and refluxed at 100 ℃ for 1 h. A solution of tosylglycine (5 mmol) in Methanol (5 mL) was added to the resultant mixture and stirred for 15 h. A saturated aqueous solution of sodium bicarbonate (60 mL) was added. The organic layers dried over sodium sulfate, and concentrated in vacuo. Recrystallize with dichloromethane to get white solid.

α-Bromoenals were prepared according to reported procedures. [3]

III. Reaction condition optimization

Table S1. Screening of different catalysts, bases, solvents and temperatures[a].

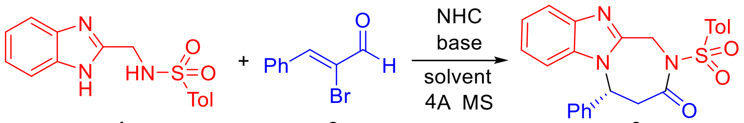

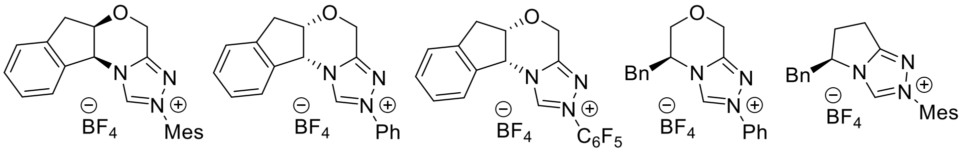

entry	NHC	base	solvent	temp.	time	yield (%)[b]	er[c]
1	A	K_2CO_3	THF	r.t.	12 h	96	1.8：98.2
2	B	K_2CO_3	THF	r.t.	12 h	69	18.6：81.4
3	C	K_2CO_3	THF	r.t.	12 h	trace	-
4	D	K_2CO_3	THF	r.t.	12 h	93	26.2：73.8
5	E	K_2CO_3	THF	r.t.	12 h	76	15.3：84.7
6	A	Cs_2CO_3	THF	r.t.	12 h	95	4.4：95.6
7	A	DBU	THF	50 °C	12 h	54	1.8：98.2
8	A	DMAP	THF	50 °C	12 h	47	1.4：98.6
9	A	t-BuOK	THF	50 °C	12 h	45	1.5：98.5
10	A	Et_3N	THF	50 °C	12 h	64	0.7：99.3
11	A	Et_3N	THF	r.t.	24 h	86	0.7：99.3
12	A	Et_3N	THF	50 °C	24 h	88	1.1：98.9
13	A	K_2CO_3	acetone	r.t.	12 h	78	0.8：99.2
14	A	K_2CO_3	CH_2Cl_2	r.t.	12 h	82	0.9：99.1
15[d]	A	K_2CO_3	THF	r.t.	12 h	48	1.1：98.9
16[d]	A	Et_3N	THF	r.t.	12 h	35	1.1：98.9
17[e]	A	K_2CO_3	THF	r.t.	12 h	99	0.7：99.3
18[f]	A	K_2CO_3	THF	r.t.	12 h	90	0.9：99.1
19[g]	A	K_2CO_3	THF	r.t.	12 h	98	1.2：98.8

[a] General reaction conditions: **2a** (0.1 mmol), **1a** (0.05 mmol), NHC (20 mol %), base (150 mol %), 4Å MS (80 mg), THF (1 mL), 12 h; [b] Isolated yield; [c] Determined by chiral HPLC analysis (IA column, 1.0 mL/min, hexans / iPrOH = 70/30); [d]**2a** (0.05 mmol), **1a** (0.1 mmol), NHC (20 mol %), base (200 mol %), 4Å MS (80 mg), solvent (1 mL), 12 h; [e] **2a** (0.2 mmol), **1a** (0.1 mmol), NHC (10 mol %), base (200 mol %), 4Å MS (150 mg), solvent (2 mL), 12 h. [f] With 5 mol % catalyst A, other conditions are the same as **e**. [g] under atmosphere, other conditions are the same as **e**. Mes = 2,4,6-Trimethylphenyl. THF =

Tetrahydrofuran. DBU = 1,8-Dizabicyclo [5.4.0] undec-7-ene. DMAP = 4-Dimethylaminopyridine. t-BuOK = Potassium tert-butoxide. Et_3N = Triethylamine. CH_2Cl_2 = Dichloromethane. r.t. = room temperature.

IV. General procedure for the catalytic reactions

Procedure for synthesis of chiral product 3a with gram-scale:

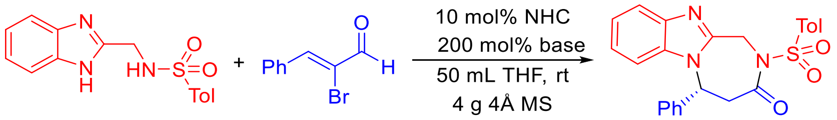

To a 100 mL Schlenk tube with a stir bar, 2-Methylaminobenzimidazole **1a** (5 mmol), 2-bromoenals **2a** (10 mmol), K_2CO_3 (10 mmol, 1.4 g), chiral NHC pre-catalyst A (0.5 mmol, 0.21 g) and 4Å molecular sieve (4 g) were added. Freshly distilled dry THF (50 mL) was added via syringe. The mixture was monitored by TLC plate until the reaction was completed (15 h) at room temperature. The mixture was concentrated, purified via column chromatography on silica gel using hexanes / EtOAc (1:1) as eluent to afford the products **3a** (2.12 g, 98% yield, 99:1).

Synthetic transformations of product 3a:

Preparation of **4**, and **5** from product **3a**:

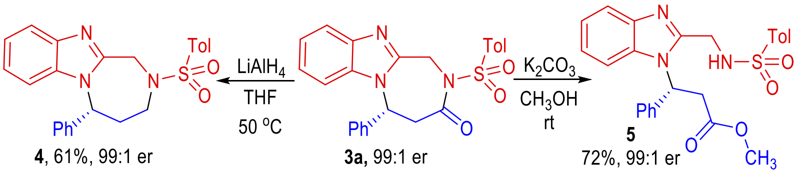

General Procedure for the Synthesis of **4**:

A 10 mL dried Schlenk tube was charged with compound **3a** (98:2 er, 0.2 mmol, 1.0 equiv) in 2 mL anhydrous THF. Subsequently, $LiAlH_4$ 2.4 mol/L in THF (2.1mL, 25.0 equiv.) was added. The mixture was stirred at 50 ℃ for 30 min until the consumption of the product **3a** as monitored by TLC. After purification by chromatography (Hexanes / EtOAc = 1:1), the desired product **4** was obtained as white solid.

General Procedure for the Synthesis of **5**:

A 10 mL Schlenk tube was added product **3a** (98:2 er, 0.2 mmol, 1.0 equiv) and potassium carbonate (0.4 mmol, 2.0 equiv) in anhydrous methanol (3 mL) at rt, the reaction system was stirred for 10 min. Then solvent was concentrated and purified by column chromatography on silica gel with hexanes / EtOAc (1:1) to give the product **5** as white solid.

V. X-ray crystallography of product 3d.

The colorless crystal of product **3d** was obtained by vaporization of a CH_2Cl_2 / n-hexane solution, and its absolute configuration was determined via X-ray structure analysis. CCDC **1987288** contains the supplementary crystallographic data that can be obtained free of charge from The Cambridge Crystallographic Data Centre via www.ccdc.cam.ac.uk/ data_request/cif.

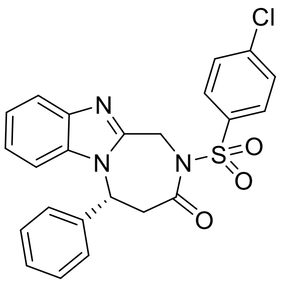

3d

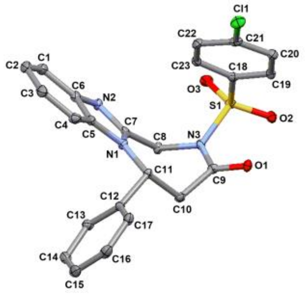

X-ray structure of **3d**

VI. Antiviral Bioassay

Extraction of potato virus Y (PVY). Viruses were propagated in *Nicotiana. tabacum* cv. K326, ground in phosphate buffer and filtered with a double-layer pledget. The extract was centrifuged for 5 min at 10000×g, and the supernatant was used as the crude extract of the virus. The extraction process was carried out at 4 ℃.

Curative activities of target compounds against PVY *in vivo*. *Chenopodium amaranticolor* plants were used to evaluate the anti-PVY activities. The crude extracts of PVY were dipped and inoculated on the whole leaves, which were scattered with silicon carbide beforehand. The leaves were washed with water after inoculation for 30 minutes and then dried. The target compound solution was smeared on the right side of *Chenopodium amaranticolor* leaves, and solvent was smeared on the left side, which served as the control. All the plants were cultivated in an incubator under an illumination of 10 000 lx at 28 ± 2 ℃. The number of local lesions appearing 5 to 6 days after inoculation was counted. Measurements were performed in triplicate.

Protective activities of target compounds against PVY *in vivo*. The target compound solution was smeared on the right side of *Chenopodium amaranticolor* leaves, and solvent was smeared on the left side, which served as the control. *Chenopodium amaranticolor* plants were inoculated with PVY after 24 hours, which were scattered with silicon carbide beforehand. The leaves were washed with water after inoculation for 30 minutes. All the plants were cultivated in an incubator under an illumination of 10 000 lx at 28 ± 2 ℃. The number of local lesions appearing 5 to 6 days after inoculation was counted. Measurements were performed in triplicate. Measurements were performed in triplicate.

Inactive activities of target compounds against PVY *in vivo*. The virus was inhibited by mixing it with compound solution with the same volume for 30min. The right side of *Chenopodium amaranticolor* leaves with silicon carbide was inoculated with the mixture, whereas the left side of each leaf, which served as the control, was inoculated with the mixture containing the solvent and the virus. All the plants were cultivated in an incubator

under an illumination of 10 000 lx at 28 ± 2 ℃. The number of local lesions appearing 5 to 6 days after inoculation was counted. Measurements were performed in triplicate.

Table S2. The Chiral Products Anti-PVY *in Vivo* at 500 μg/mL [a]

Compd.	Curative Effect (%)	Protective Effect (%)	Inactive Effect (%)	Compd.	Curative Effect (%)	Protective Effect (%)	Inactive Effect (%)
**3a** (*R*)	48.6±3.9	47.1±4.5	54.1±2.6	**3n** (*R*)	51.3±3.3	59.6±3.5	78.5±3.7
- **3a** (*S*)	54.4±3.9	56.2±3.4	61.4±3.9	- **3n** (*S*)	60.2±4.3	63.9±4.1	83.4±4.6
± **3a** (*rac*)	50.0±3.5	51.3±3.1	57.6±4.3	± **3n** (*rac*)	53.0±4.6	53.2±2.1	76.2±3.9
**3b** (*R*)	47.5±2.9	51.2±3.3	78.4±2.8	**3o** (*R*)	29.3±2.8	34.3±3.1	44.1±3.4
- **3b** (*S*)	51.4±1.8	53.5±3.7	66.5±3.3	- **3o** (*S*)	32.2±3.5	38.4±4.7	47.3±3.8
± **3b** (*rac*)	48.5±3.1	50.3±3.4	68.7±2.9	± **3o** (*rac*)	29.2±3.4	32.1±3.2	42.9±4.7
**3c** (*R*)	44.6±4.4	42.1±2.9	55.3±3.6	**3p** (*R*)	50.8±2.7	53.1±3.4	76.5±4.2
- **3c** (*S*)	49.5±4.3	49.2±3.2	63.5±3.3	- **3p** (*S*)	52.9±3.4	56.0±3.9	74.5±4.2
± **3c** (*rac*)	43.2±4.2	42.5±4.6	60.6±2.5	± **3p** (*rac*)	49.6±2.1	52.8±4.5	76.0±2.0
**3d** (*R*)	39.7±2.6	42.6±2.5	52.2±2.6	**3q** (*R*)	46.5±4.6	51.1±3.3	69.4±3.8
- **3d** (*S*)	43.7±2.2	45.6±3.4	64.4±1.9	- **3q** (*S*)	43.7±4.0	45.1±3.7	74.8±3.8
± **3d** (*rac*)	38.3±1.7	42.7±3.1	54.5±4.2	± **3q** (*rac*)	43.3±3.7	47.7±4.4	71.5±2.1
**3e** (*R*)	43.5±2.8	52.4±1.5	62.3±3.7	**3r** (*R*)	53.5±2.4	57.7±4.2	70.3±4.1
- **3e** (*S*)	51.3±1.8	55.7±3.6	64.6±4.5	- **3r** (*S*)	57.1±3.2	55.1±3.4	79.7±1.1
± **3e** (*rac*)	41.4±1.5	50.7±3.6	65.9±3.9	± **3r** (*rac*)	51.2±1.6	54.2±4.2	78.8±3.9
**3f** (*R*)	41.3±4.3	48.5±3.4	54.5±3.1	**3s** (*R*)	47.2±4.2	49.6±3.3	51.7±3.2
- **3f** (*S*)	47.2±4.3	49.9±1.5	59.6±3.5	- **3s** (*S*)	50.7±3.4	53.5±1.5	58.5±3.6
± **3f** (*rac*)	42.6±3.5	50.5±2.5	59.8±4.8	± **3s** (*rac*)	48.1±2.2	50.4±2.4	54.7±3.9
**3g** (*R*)	39.6±2.7	44.1±4.2	53.8±3.1	**3u** (*R*)	38.4±2.7	42.6±3.7	49.6±4.3
- **3g** (*S*)	46.4±2.6	47.3±2.7	59.8±3.6	- **3u** (*S*)	47.8±2.6	48.6±2.3	55.9±3.9
± **3g** (*rac*)	40.8±3.4	44.3±2.4	58.9±4.0	± **3u** (*rac*)	40.7±2.8	40.8±1.8	52.4±2.0
**3h** (*R*)	43.8±4.5	45.7±4.4	54.2±4.7	**3v** (*R*)	44.6±4.3	47.2±4.1	47.3±4.2
- **3h** (*S*)	48.6±1.5	49.3±3.1	58.9±3.9	- **3v** (*S*)	51.7±3.7	53.2±2.7	63.4±2.0
± **3h** (*rac*)	45.6±4.1	44.4±2.4	56.1±4.9	± **3v** (*rac*)	45.7±3.7	48.9±4.2	55.6±4.7
**3i** (*R*)	43.3±3.9	40.5±3.4	50.7±4.4	**3w** (*R*)	44.7±3.2	51.7±4.6	62.7±3.8
- **3i** (*S*)	50.4±3.1	46.2±3.3	52.3±1.2	- **3w** (*S*)	46.2±3.6	53.2±1.9	74.1±4.4
± **3i** (*rac*)	44.3±2.2	42.3±2.9	54.5±4.2	± **3w** (*rac*)	43.6±2.8	50.5±4.0	70.1±4.8
**3j** (*R*)	55.3±4.5	57.3±4.6	80.5±4.2	**3x** (*R*)	55.2±4.3	54.7±3.3	64.2±4.6
- **3j** (*S*)	61.0±3.8	63.2±4.0	85.4±4.4	- **3x** (*S*)	51.4±4.0	57.8±3.4	73.8±4.0
± **3j** (*rac*)	54.0±2.1	53.4±3.2	79.9±3.7	± **3x** (*rac*)	52.7±3.6	54.4±2.2	71.9±4.4
**3k** (*R*)	48.3±4.5	49.0±4.7	60.7±4.7	**3y** (*R*)	45.6±3.3	44.5±4.7	52.0±3.9
- **3k** (*S*)	53.1±2.6	51.5±3.2	68.1±3.7	- **3y** (*S*)	50.4±2.1	47.3±3.8	57.0±3.8
± **3k** (*rac*)	49.5±1.7	48.7±2.2	66.9±3.4	± **3y** (*rac*)	46.7±2.0	44.9±4.0	55.9±4.5
**3l** (*R*)	52.2±1.9	55.4±4.1	71.5±4.4	**3z** (*R*)	53.3±2.2	57.2±3.3	71.4±4.8
- **3l** (*S*)	55.4±3.7	58.2±1.7	78.4±1.8	- **3z** (*S*)	58.4±2.9	59.5±3.0	73.0±2.9
± **3l** (*rac*)	52.6±3.4	53.6±4.3	75.4±2.2	± **3z** (*rac*)	53.8±3.5	56.7±3.0	74.8±3.2
**3m** (*R*)	51.1±3.7	54.3±2.3	64.2±4.2	Ribavirin[b]	45.5±2.4	48.2±1.9	63.5±2.1
- **3m** (*S*)	46.8±4.4	50.1±4.0	63.3±2.4				
± **3m** (*rac*)	47.4±4.0	51.0±3.8	63.4±4.0				

[a]Average of three replicates. [b]Ribavirin were used as control.

VII. Mechanism of Action

Experimental Section

Molecular Docking. The drug discovery modeling software (Autodock 4.0) was used for molecular docking. The crystal structures of PVY CP (PDB ID: 6HXZ) downloaded from http://www.rcsb.org was used as templates for molecular docking. The compound **-3j** (*S*) was built and optimized, and their lowest energy conformations were performed as the initial docking conformation. Molecular dynamics simulation was performed by AMBER software.

Microscale thermophoresis (MST) assay. The binding affinity between compound **-3j** (*S*) and wild-type or mutanted PVY CP were determined by Monolith NT.115 software (NanoTemper Technologies, Munich, Germany). Briefly, A range of ligands compound **-3j** (*S*) from 0 μM to 5 μM were incubated with 0.5 μM of purified wild-type or mutanted PVY CP for 5 min with a NT-647 dye (Nano Temper Technologies). After incubation, the mixed samples were loaded into the capillaries and analyzed by the NanoTemper software to assess the equilibrium dissociation constant.

Plant growth, virus inoculation, and protein transient expression. A greenhouse was used to cultivate *N. tabacum* cv. K326, *N. benthamiana*, and *Chenopodium Amaranticolor* plants maintained at a 6/18-hour (dark/light) photoperiod and 25˚C. Individual plasmids were transformed into the GV3101 strain of agrobacterium tumefaciens. Cultivation of agrobacterium cultures containing different plasmids, dilution of agrobacterium cultures, and transient expression analysis refer to previous reports.[4]

RNA extraction, RT-PCR, and RT-qPCR. The leaves of the assayed *N. benthamiana* were used to extract total RNA by TransZol reagent (TransGen Biotech, Beijing, China), and DNA contamination was eliminated by a gDNA wipe enzyme (Vazyme, Nanjing, China). Reverse transcription was carried out using gene-specific primers or random primers and a reverse transcriptase kit (Vazyme, Nanjing, China) according to the instructions. The taq DNA polymerase (Vazyme, Nanjing, China) was used to execute PCR. RT-qPCR was

conducted using the SYBR Green qPCR mix (Vazyme, Nanjing, China). The primers mentioned in this study are described in Table S3.

Western blot assay. The leaves of the assayed *N. benthamiana* were used to extract total protein according to previously described.[5] An anti-GFP (Proteintech, Wuhan, China) or anti-PVY CP (Youke, Shanghai, China) antibody and horseradish peroxidase-conjugated goat anti-rabbit IgG (Proteintech, Wuhan, China) were individually used as the primary and secondary antibody. The signal of target protein was visualized by a ChemiDoc MP Imaging System (Bio-Rad).

Trypan blue staining. The systematic leaves of *N. tabacum* cv. K326 were harvested, photographed, and stained for the veinal necrosis through trypan blue staining. The leaves were immersed in a trypan blue solution (0.15 g of Trypan blue, 400 mL absolute ethanol, 100 ml glycerol, 100 ml sterile water, 100 ml lactic acid, and 100 ml water phenol) were boiled for 3–5 min. The stained leaves were de-stained in a chloral hydrate solution (2.5 g/mL) through three rinsesand photographed.

Virus particle purification. *N. benthamiana* systematic leaves after 7 days of infection with wild-type or mutanted PVY were collected for virion particle extraction. The purification method of virions particle was based on our previously described. The PVY-containing supernatant was adsorbed by the copper mesh carbon support film (Zhongjingkeyi, Beijing, China) and counterstained with 1% phosphotungstic acid buffer. The morphology of PVY particles was observed by transmission electron microscopy (TEM, Talos F200C, FEI, USA).

Confocal microscopy and GFP imaging. The agrobacterium-infiltrated leaf patches were monitored under the confocal microscope (Carl Zeiss, Germany) to examine subcellular localization and intercellular movement in *N. benthamiana* plants. The emission wavelengths and excitation for GFP fluorescence observations were individually set at 520 to 540 and 488 nm. The fluorescence pictures were processed by ZEN 2.1. The PVY-

infiltrated *N. benthamiana* plants were photographed under a hand-held UV lamp (365 nm, LUYOR).

Enzyme-linked immunosorbent assay (ELISA). The leaves of the assayed *N. benthamiana* were individually collected for ELISA. anti-PVY CP (Youke, Shanghai, China) or anti-GFP (Proteintech, Wuhan, China) antibody and horseradish peroxidase-conjugated goat anti-rabbit IgG (Proteintech, Wuhan, China) were used as the primary and secondary antibody, respectively. *p*-Nitrophenyl phosphate (Sigma-Aldrich) was the substrate as detection reagent. The optical density at 405 nm of the assayed sample was detected by a Multifunction Microplate Reader (BioTek, U.S.A.).

Molecular dynamics simulation. After molecular docking, the system was simulated by molecular dynamics. Molecular dynamics simulation of compound -**3j** (*S*) was carried out using AMBER software. It was performed according to the standardized procedures. Then, the binding free energy (ΔG) of compound -**3j** (*S*) with PVY CP was computed using the molecular mechanics Poisson–Boltzmann surface area (MMPBSA) method, and the contributions of residues to the ligand were determined using the deconstruct module.

Co-immunoprecipitation. The desired proteins were co-expressed in *N. benthamiana* leaf tissue using Agrobacterium-mediated transient expression. The tissue was frozen in liquid nitrogen and ground using a mortar and pestle. The samples were suspended in immunoprecipitation buffer and centrifuged (10 min). Proteins were immunoprecipitated for 1 h using Myc Trap Beads (Beyotime, Shanghai, China). The samples were washed five times with immunoprecipitation buffer, before elution by boiling with loading buffer. The Anti-Myc primary antibody (Proteintech, Wuhan, China) was used for the anti-Myc Western blot followed by secondary anti-mouse alkaline phosphatase-conjugated antibody (Proteintech, Wuhan, China). The anti-GFP Western blot were performed using anti-GFP antibody (Proteintech, Wuhan, China) as the primary antibody and anti-mouse alkaline phosphatase-conjugated secondary antibodies (Proteintech, Wuhan, China). Sequence data of *NtCPIP* from the GenBank data libraries under accession numbers AY319648.

Results

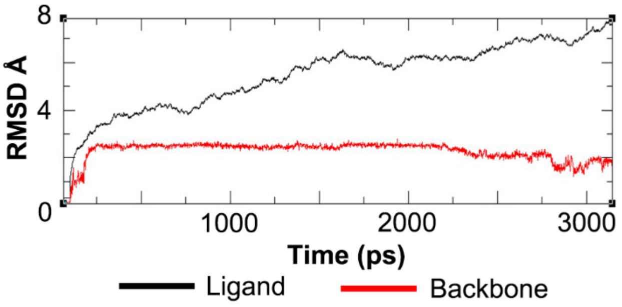

Figure S1. Molecular dynamics simulation studies of compound **-3j** (*S*).

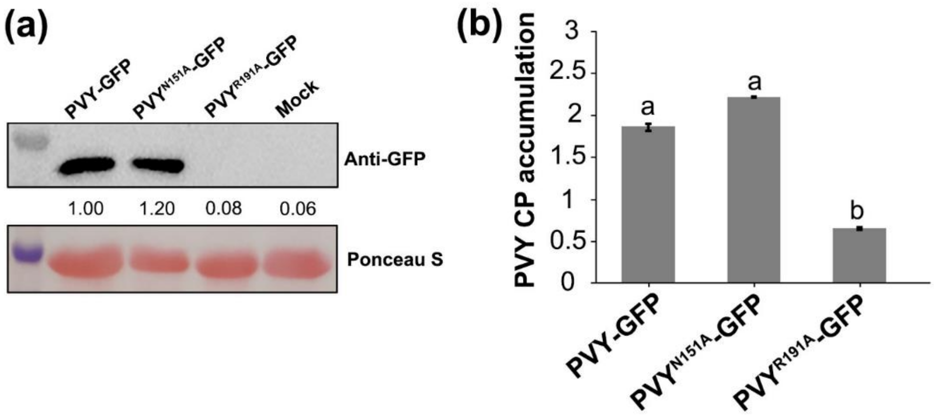

Figure S2. (a) and (b) The accumulation levels of green fluorescent protein (GFP) and PVY CP in the systemically infected leaves of the wild-type and mutated PVY-infected *N. benthamiana* plants were analyzed at 7 dpai by Western blot and enzyme-linked immunosorbent assay, respectively. Staining of RuBisCO with Ponceau S was used as a sample loading control.

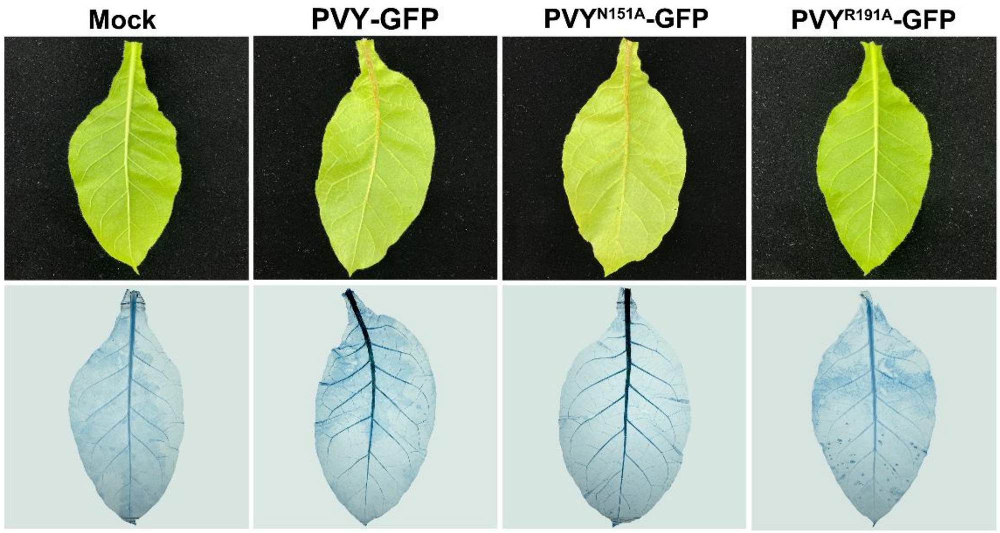

Figure S3. The symptoms of *N. tabacum* cv. K326 induced by wild-type or mutated PVY-GFP at 21 dpai. The systematic leaves were harvested (upper panel), and then stained with trypan blue solution (lower panel).

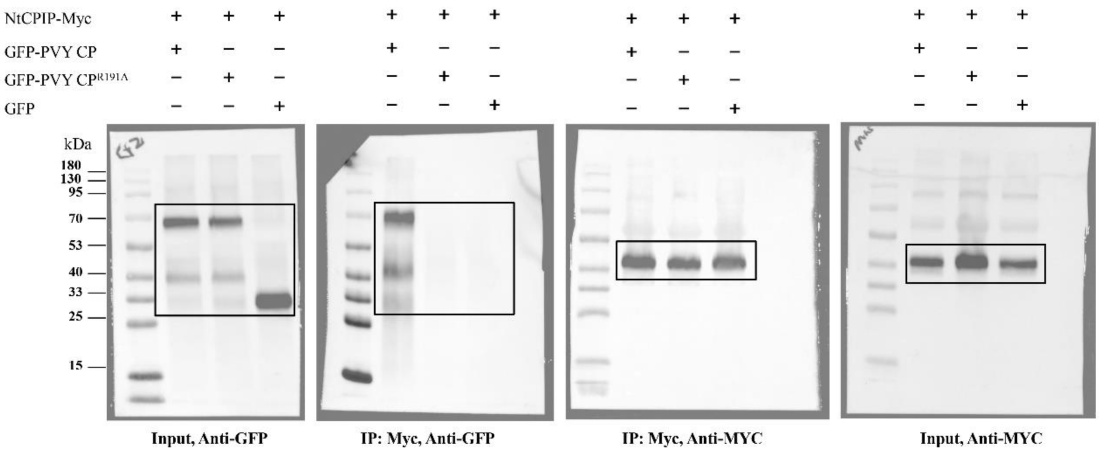

Figure S4. The raw images of Figure 6a. Co-immunoprecipitation assays on *N. benthamiana* using a Myc-tagged NtCPIP construct and GFP-PVY CP or GFP-PVY CP^{R191A} at 4 days postagroinfiltration.

Primers used in this study

Table S3. Primers used in this study

Primes	Sequences 5'→3'
PVY-N151A-F	GTTGAGGCTGCAAAACCAACCCTTAGGCAAATCAT
PVY-N151A-R	TTTTGCAGCCTCAACGATTGGTTTCAACGGGTACTC
PVY-R191A-F	AATCTGGCGGATGTGGGTTTAGCGCGTTATGC

PVY-R191A-R	ACATCCGCCAGATTTCGAATTAAACCATATCGTGG
GFP-CDS-F	GAATTCTAAGAGGAGTCCACC
GFP-CDS-R	ACCGCCAGAACCTCCTTTGTAGAGCTCATCCATGCC
PD-GFP-PVY CP-F	GGAGGTTCTGGCGGTatgGCAAATGACACAATCGATGC
PD-GFP-PVY CP-R	CTCCTCTTAGAATTCttaCATGTTCTTAACTCCAAGTAGAG
qPCR-PVY CP-F	TGGCGAGGTTCCATTTTCA
qPCR-PVY CP-R	CATAGGAGAAACTGAGATGCCAACT
PVY-NIB-ΔGDD-F	TTTGTTAATTTATTGATTGCTGTGAATCCGGAGAAAGAG
PVY-NIB-ΔGDD-R	GCAATCAATAAATTAACAAAGAATACACACGTGCTGTCG
qPCR-Actin-F	CACACTGGAGTGATGGTTGG
qPCR-Actin-R	GGTGTGGTGCCAAATCTTCT
qPCR-EF1α-F	GACAAGCGTGTTATTGAGAGG
qPCR-EF1α-R	CACAGTGCAGTAGTACTTAGTG
PVY-F	GTACCGGATTATGCCGGAAATGACACAATCGATGC
PVY-R	CTCTTAGAATTCTCACATGTTCTTCACTCCAAGTAGA
PD-NtCPIP-F	tcaaaccacgttcataATGGGCGTTGATTACTACAA
PD-NtCPIP-R	CTATAAGTCTTCTTCGGATATCAGCTTCTGTTCGTCAACAGT CCTGCCCAGCACTCTT

References:

[1]. H. Xu, H. Tian, L. Zheng, Q. Liu, L. Wang, S. Zhang, *J. Heterocyclic Chem.* **2012**, *49*, 1108.

[2]. D. I. Ugwu, U. C. Okoro, N. K. Mishra, *PLoS One* **2018**, *13*, e0191234.

[3]. a) F. G. Sun, L. H. Sun, S. Ye, *Adv. Synth. Catal.* **2011**, *353*, 3134; b) Y. Liu, J. Chen, Z. Zhang, J. Qin, M. Zhao, W. Zhang, *Org. Biomol. Chem.* **2016**, *14*, 7099.

[4] Z. Y. Yan, D. J. Cheng, L. Z. Liu, C. Geng, Y. P. Tian, X. D. Li, J. P. T. Valkonen, *Mol. Plant Pathol.* **2021**, *22*, 189-203.

[5] J. Li, H. Feng, S. Liu, P. Liu, X. Chen, J. Yang, L. He, J. Yang, J. Chen, *PLOS Pathog.* **2022**, *18*, e1010412.

VIII. Characterization of products

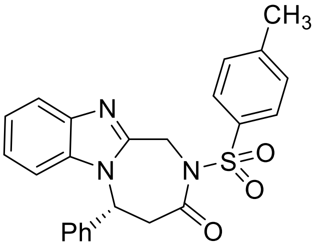

3a

(*R*)-5-phenyl-2-tosyl-1,2,4,5-tetrahydro-3*H*-benzo[4,5]imidazo[1,2-a][1,4]diazepin-3-one (3a)

White solid, m.p. 193-195 ℃, 99% yield.

[α] $^{28}_{D}$ = -141.1 (c 1.0 $CHCl_3$).

^{1}H NMR (300 MHz, $CDCl_3$) δ 7.79 (d, *J* = 8.4 Hz, 3H), 7.22 (d, *J* = 8.1 Hz, 2H), 7.15 (d, *J* = 7.6 Hz, 4H), 7.10 – 7.02 (m, 1H), 6.78 (d, *J* = 7.3 Hz, 2H), 6.71 (d, *J* = 8.2 Hz, 1H), 5.74 (d, *J* = 17.5 Hz, 1H), 5.63 (dd, *J* = 8.4, 4.6 Hz, 1H), 5.56 (d, *J* = 17.5 Hz, 1H), 3.51 (dd, *J* = 14.2, 4.6 Hz, 1H), 3.32 (dd, *J* = 14.2, 8.4 Hz, 1H), 2.35 (s, 3H).

^{13}C NMR (75 MHz, $CDCl_3$) δ 167.6, 147.4, 145.1, 141.8, 138.1, 135.3, 135.1, 129.3, 129.2, 128.8, 128.6, 125.2, 123.6, 123.0, 120.2, 111.2, 57.4, 44.7, 44.1, 21.5.

HRMS (ESI, m/z): calculated for $C_{24}H_{22}N_3O_3S$ [M+H]$^+$: 432.1376, found: 432.1368.

HPLC analysis: 99.2:0.8 e.r. (Chiralpak IA, Hexane/2-PrOH = 70/30, 1.0 mL/min), Rt (major) = 19.2 min, Rt (minor) = 13.5 min.

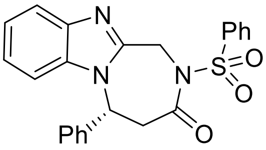

3b

(*R*)-5-phenyl-2-(phenylsulfonyl)-1,2,4,5-tetrahydro-3*H*-benzo[4,5] imidazo[1,2-a][1,4] diazepin-3-one (3b)

White solid, m.p. 190-191 ℃, 97% yield.

[α] $^{28}_{D}$ = -500.9 (c 1.0 $CHCl_3$).

^{1}H NMR (500 MHz, $CDCl_3$) δ 7.90 (d, *J* = 8.0 Hz, 2H), 7.80 (d, 1H), 7.53 (t, *J* = 7.2 Hz, 1H), 7.40 – 7.34 (m, 2H), 7.26 – 7.18 (m, 2H), 7.13 (t, *J* = 7.6 Hz, 2H), 7.07 (t, *J* = 7.7 Hz, 1H), 6.76 (d, *J* = 7.8 Hz, 2H), 6.71 (d, *J* = 8.2 Hz, 1H), 5.78 (d, *J* = 17.1 Hz, 1H), 5.63 (dd, *J* = 8.1, 4.5 Hz, 1H), 5.54 (d, *J* = 17.4 Hz, 1H), 3.54 (dd, *J* = 14.2, 4.6 Hz, 1H), 3.30 (dd, *J* = 14.3, 8.1 Hz, 1H).

^{13}C NMR (126 MHz, $CDCl_3$) δ 167.7, 147.4, 141.7, 138.2, 137.9, 135.0, 133.9, 129.2, 128.8, 128.7, 128.6, 125.2, 123.7, 123.1, 120.2, 111.2, 57.3, 44.7, 43.9.

HRMS (ESI, m/z): Mass calcd for $C_{23}H_{20}O_3N_3S$ [M+H]$^+$: 418.1219; found 418.1207.

HPLC analysis: 99.6:0.4 e.r. (Chiralpak IA, Hexane/2-PrOH = 70/30, 1.0 mL/min), Rt (major) = 18.7 min, Rt (minor) = 14.5 min.

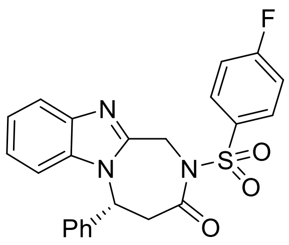

3c

(*R*)-2-((4-fluorophenyl)sulfonyl)-5-phenyl-1,2,4,5-tetrahydro-3*H*-benzo[4,5]imidazo [1,2-a][1,4]diazepin-3-one (3c)

White solid, m.p. 197-198 ℃, 97% yield.

[α] $^{28}_{D}$ = -1674.4 (c 1.0 $CHCl_3$).

^{1}H NMR (500 MHz, $CDCl_3$) δ 7.93 (dd, *J* = 8.7, 5.0 Hz, 2H), 7.79 (d, *J* = 8.1 Hz, 1H), 7.23 (d, *J* = 7.6 Hz, 2H), 7.16 (t, *J* = 7.5 Hz, 2H), 7.08 (t, *J* = 7.7 Hz, 1H), 7.02 (t, *J* = 8.6 Hz, 2H), 6.77 (d, *J* = 7.6 Hz, 2H), 6.72 (d, *J* = 8.2 Hz, 1H), 5.75 (d, *J* = 17.5 Hz, 1H), 5.64 (dd, *J* = 8.4, 4.5 Hz, 1H), 5.55 (d, *J* = 17.5 Hz, 1H), 3.54 (dd, *J* = 14.3, 4.5 Hz, 1H), 3.33 (dd, *J* = 14.3, 8.3 Hz, 1H).

^{13}C NMR (126 MHz, $CDCl_3$) δ 167.7, 166.8, 164.8, 147.1, 141.7, 137.9, 135.0, 134.0, 134.0, 131.9, 131.8, 129.2, 128.7, 125.1, 123.8, 123.1, 120.2, 116.1, 115.9, 111.2, 57.2, 44.7, 43.9.

^{19}F NMR (282 MHz, $CDCl_3$) δ -98.5.

HRMS (ESI, m/z): calculated for $C_{23}H_{19}O_3N_3FS$ $[M+H]^+$: 436.1125, found: 436.1112.

HPLC analysis: 98.7:1.3 e.r. (Chiralpak IA, Hexane/2-PrOH = 70/30, 1.0 mL/min), Rt (major) = 22.2 min, Rt (minor) = 13.9 min.

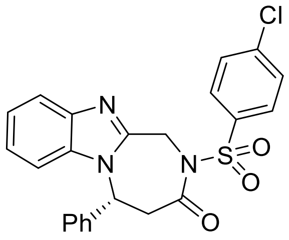

3d

(*R*)-2-((4-chlorophenyl)sulfonyl)-5-phenyl-1,2,4,5-tetrahydro-3*H*-benzo[4,5]imidazo [1,2-a][1,4]diazepin-3-one (3d)

White solid, m.p. 198-200 ℃, 98% yield.

[α] $^{28}_{D}$ = -655.8 (c 1.0 $CHCl_3$).

^{1}H NMR (500 MHz, $CDCl_3$) δ 7.83 (d, *J* = 8.4 Hz, 2H), 7.78 (d, *J* = 8.1 Hz, 1H), 7.31 (d, *J* = 8.4 Hz, 2H), 7.26 – 7.20 (m, 2H), 7.15 (t, *J* = 7.6 Hz, 2H), 7.08 (t, *J* = 7.7 Hz, 1H), 6.74 (d, *J* = 6.7 Hz, 3H), 5.76 (d, *J* = 17.5 Hz, 1H), 5.65 (dd, *J* = 8.2, 4.5 Hz, 1H), 5.52 (d, *J* = 17.5 Hz, 1H), 3.56 (dd, *J* = 14.2, 4.5 Hz, 1H), 3.31 (dd, *J* = 14.2, 8.2 Hz, 1H).

^{13}C NMR (101 MHz, $CDCl_3$) δ 167.7, 147.1, 141.7, 140.7, 137.8, 136.5, 135.0, 130.3, 129.2, 129.0, 128.7, 125.1, 123.8, 123.1, 120.2, 111.2, 57.2, 44.7, 43.8.

HRMS (ESI, m/z): calculated for $C_{23}H_{19}O_3N_3ClS$ $[M+H]^+$: 452.0830, found: 452.0811.

HPLC analysis: 99.6:0.4 e.r. (Chiralpak IA, Hexane/2-PrOH = 70/30, 1.0 mL/min), Rt (major) = 25.3 min, Rt (minor) = 13.6 min.

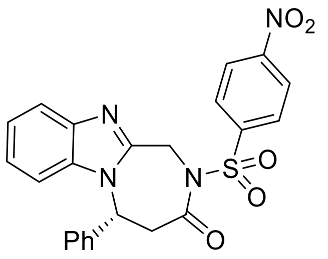

3e

(*R*)-2-((4-nitrophenyl)sulfonyl)-5-phenyl-1,2,4,5-tetrahydro-3*H*-benzo[4,5]imidazo [1,2-a][1,4]diazepin-3-one (3e)

White solid, m.p. 193-194 ℃, 99% yield.

[α] $^{28}{}_{D}$ = -358.5 (c 1.0 $CHCl_3$).

^{1}H NMR (500 MHz, DMSO-d_6) δ 8.36 (d, *J* = 8.5 Hz, 2H), 8.14 (d, *J* = 8.6 Hz, 2H), 7.71 (d, *J* = 8.0 Hz, 1H), 7.21 (t, *J* = 7.7 Hz, 1H), 7.18 (d, *J* = 7.3 Hz, 1H), 7.16 (d, *J* = 7.5 Hz, 2H), 7.10 (t, *J* = 7.6 Hz, 1H), 6.89 (t, *J* = 7.8 Hz, 3H), 6.10 – 6.05 (m, 1H), 5.82 (d, *J* = 17.4 Hz, 1H), 5.67 (d, *J* = 17.3 Hz, 1H), 4.03 (dd, *J* = 14.7, 4.1 Hz, 1H), 3.26 (dd, *J* = 14.9, 6.5 Hz, 1H).

^{13}C NMR (75 MHz, DMSO) δ 169.7, 150.9, 148.5, 143.7, 141.9, 139.0, 135.4, 130.4, 129.0, 128.4, 125.9, 124.7, 123.4, 122.8, 119.9, 111.9, 56.2, 44.7, 43.1.

HRMS (ESI, m/z): calculated for $C_{23}H_{19}O_5N_4S$ $[M+H]^+$: calcd 463.1070, found: 463.1052.

HPLC analysis: 99.6:0.4 e.r. (Chiralpak IA, Hexane/2-PrOH = 70/30, 1.0 mL/min), Rt (major) = 37.3 min, Rt (minor) = 23.7 min.

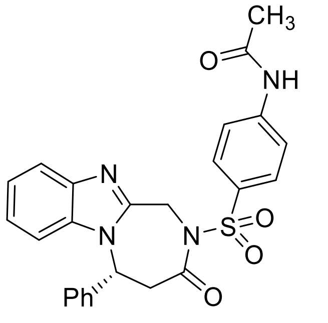

3f

(*R*)-N-(4-((3-oxo-5-phenyl-4,5-dihydro-1*H*-benzo[4,5]imidazo[1,2-a][1,4]diazepin-2 (3H)-yl)sulfonyl)phenyl)acetamide (3f)

White solid, m.p. 203-204 ℃, 91% yield.

[α] $^{28}{}_{D}$ = -2081.1 (c 1.0 $CHCl_3$).

^{1}H NMR (400 MHz, $CDCl_3$) δ 8.05 (s, 1H), 7.79 (t, *J* = 7.9 Hz, 3H), 7.47 (d, *J* = 8.6 Hz, 2H), 7.26 (d, *J* = 7.7 Hz, 1H), 7.22 (d, *J* = 7.3 Hz, 1H), 7.17 (t, *J* = 7.1 Hz, 2H), 7.12 – 7.05 (m, 1H), 6.81 (d, *J* = 7.0 Hz, 2H), 6.74 (d, *J* = 8.2 Hz, 1H), 5.78 (d, *J* = 17.5 Hz, 1H), 5.68 (dd, *J* = 7.9, 4.4 Hz, 1H), 5.52 (d, *J* = 17.5 Hz, 1H), 3.58 (dd, *J* = 14.3, 4.5 Hz, 1H), 3.31 (dd, *J* = 14.3, 8.0 Hz, 1H), 2.16 (s, 3H).

^{13}C NMR (101 MHz, $CDCl_3$) δ 168.7, 167.9, 147.3, 143.3, 141.6, 137.8, 135.0, 132.3, 130.1, 129.3, 128.7, 125.2, 123.8, 123.1, 120.1, 118.7, 111.3, 57.3, 44.7, 44.0, 24.6.
HRMS (ESI, m/z): calculated for $C_{25}H_{21}O_4N_4S$ $[M+H]^+$: 473.1278, found: 473.1287.
HPLC analysis: 99.9:0.1 e.r. (Chiralpak IA, Hexane/2-PrOH = 70/30, 1.0 mL/min), Rt (major) = 17.7 min, Rt (minor) = 11.6 min.

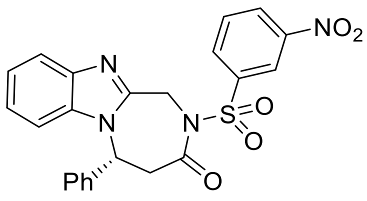

3g

(*R*)-2-((3-nitrophenyl)sulfonyl)-5-phenyl-1,2,4,5-tetrahydro-3*H*-benzo[4,5]imidazo [1,2-a][1,4]diazepin-3-one (3g)

White solid, m.p. 189-190 ℃, 99% yield.
$[\alpha]^{28}_D$ = -1176.5 (c 1.0 $CHCl_3$).
^1H NMR (500 MHz, $CDCl_3$) δ 8.75 (s, 1H), 8.33 (d, *J* = 8.3 Hz, 1H), 8.21 (d, *J* = 7.9 Hz, 1H), 7.78 (d, *J* = 8.2 Hz, 1H), 7.55 (t, *J* = 8.1 Hz, 1H), 7.24 (t, *J* = 7.9 Hz, 2H), 7.21 – 7.16 (m, 2H), 7.08 (t, *J* = 7.7 Hz, 1H), 6.84 (d, *J* = 7.0 Hz, 2H), 6.73 (d, *J* = 8.2 Hz, 1H), 5.73 (d, *J* = 17.5 Hz, 1H), 5.68 – 5.59 (m, 2H), 3.52 (dd, *J* = 14.3, 4.6 Hz, 1H), 3.41 (dd, *J* = 14.2, 8.9 Hz, 1H).
^{13}C NMR (126 MHz, $CDCl_3$) δ 168.1, 147.8, 146.7, 141.7, 140.1, 137.8, 135.0, 134.2, 130.0, 129.3, 128.8, 128.3, 125.1, 124.3, 124.0, 123.3, 120.4, 111.1, 57.1, 44.9, 43.9.
HRMS (ESI, m/z): calculated for $C_{23}H_{19}O_5N_4S$ $[M+H]^+$: 463.1070, found: 463.1055.
HPLC analysis: 99.7:0.3 e.r. (Chiralpak IA, Hexane/2-PrOH = 70/30, 1.0 mL/min), Rt (major) = 24.5 min, Rt (minor) = 21.8 min.

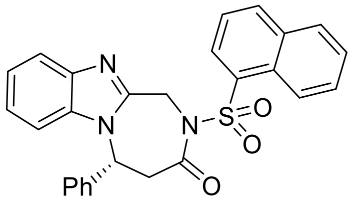

3h

(*R*)-2-(naphthalen-1-ylsulfonyl)-5-phenyl-1,2,4,5-tetrahydro-3*H*-benzo[4,5]imidazo [1,2-a][1,4]diazepin-3-one (3h)

White solid, m.p. 203-204 ℃, 99% yield.
$[\alpha]^{28}_D$ = 342.3 (c 1.0 $CHCl_3$).
^1H NMR (500 MHz, Chloroform-*d*) δ 8.44 (dd, *J* = 7.5, 1.3 Hz, 1H), 8.30 (d, *J* = 8.6 Hz, 1H), 8.04 (d, *J* = 8.1 Hz, 1H), 7.91 – 7.78 (m, 2H), 7.48 (t, *J* = 7.8 Hz, 1H), 7.38 – 7.29 (m, 1H), 7.25 (t, *J* = 7.7 Hz, 1H), 7.19 – 7.11 (m, 1H), 7.01 (td, *J* = 7.8, 3.0 Hz, 2H), 6.77 (t, *J* = 7.6 Hz, 2H), 6.58 (d, *J* = 8.1 Hz, 1H), 6.35 (d, *J* = 7.6 Hz, 2H), 6.07 (d, *J* = 17.5 Hz, 1H), 5.54 (d, *J* = 17.5 Hz, 1H), 5.48 (dd, *J* = 7.0, 4.3 Hz, 1H), 3.55 (dd, *J* = 14.3, 4.3 Hz, 1H), 3.12 (dd, *J* = 14.4, 7.0 Hz, 1H).

^{13}C NMR (101 MHz, $CDCl_3$) δ 167.5, 147.4, 141.9, 137.6, 135.4, 135.1, 133.8, 133.8, 133.0, 129.0, 128.9, 128.6, 128.3, 127.9, 126.8, 125.0, 124.0, 123.5, 123.4, 123.0, 120.0, 111.3, 57.2, 44.4, 43.9.
HRMS (ESI, m/z): calculated for $C_{27}H_{22}O_3N_3S$ $[M+H]^+$:468.1376, found: 468.1362.
HPLC analysis: 99.7:0.3 e.r. (Chiralpak IA, Hexane/2-PrOH = 70/30, 1.0 mL/min), Rt (major) = 30.8 min, Rt (minor) = 17.8 min.

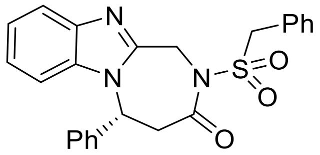

3i

(*R*)-2-(benzylsulfonyl)-5-phenyl-1,2,4,5-tetrahydro-3*H*-benzo[4,5]imidazo[1,2-a][1,4] diazepin-3-one (3i)

White solid, m.p. 198-199 ℃, 98% yield.
[α] $^{28}_{D}$ = -3946.5 (c 1.0 $CHCl_3$).
^1H NMR (400 MHz, $CDCl_3$) δ 7.62 (d, *J* = 8.1 Hz, 1H), 7.32 (d, *J* = 6.3 Hz, 3H), 7.20 (t, *J* = 7.7 Hz, 1H), 7.16 (d, *J* = 8.1 Hz, 2H), 7.10 – 6.98 (m, 3H), 6.88 (t, *J* = 7.6 Hz, 2H), 6.71 (t, *J* = 7.5 Hz, 1H), 6.66 (d, *J* = 8.2 Hz, 1H), 5.54 (dd, *J* = 10.9, 4.1 Hz, 1H), 5.16 (d, *J* = 17.4 Hz, 1H), 5.01 (d, *J* = 17.4 Hz, 1H), 4.90 (d, *J* = 14.1 Hz, 1H), 4.55 (d, *J* = 14.1 Hz, 1H), 3.47 (dd, *J* = 14.3, 11.0 Hz, 1H), 3.29 (dd, *J* = 14.3, 4.2 Hz, 1H).
^{13}C NMR (101 MHz, $CDCl_3$) δ 169.5, 146.2, 142.0, 138.7, 135.0, 129.8, 129.5, 129.0, 128.9, 128.4, 126.8, 125.4, 123.4, 122.8, 120.3, 111.3, 59.7, 57.5, 44.6, 44.3.
HRMS (ESI, m/z): calculated for $C_{24}H_{22}O_3N_3S$ $[M+H]^+$: 432.1376, found: 432.1363.
HPLC analysis: 96.8: 3.2 e.r. (Chiralpak IA, Hexane/2-PrOH = 70/30, 1.0 mL/min), Rt (major) = 20.3 min, Rt (minor) = 17.8 min.

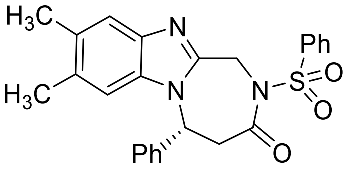

3j

(*R*)-8,9-dimethyl-5-phenyl-2-(phenylsulfonyl)-1,2,4,5-tetrahydro-3*H*-benzo[4,5]imidazo[1,2-a][1,4]diazepin-3-one (3j)

White solid, m.p. 204-206 ℃, 92% yield.
[α] $^{25}_{D}$ = 3.4 (c = 0.5 in $CHCl_3$).
^1H NMR (400 MHz, $CDCl_3$) δ 7.89 (d, J = 7.3 Hz, 2H), 7.52 (s, 2H), 7.36 (t, J = 7.9 Hz, 2H), 7.24 – 7.16 (m, 1H), 7.12 (t, J = 7.4 Hz, 2H), 6.73 (d, J = 7.3 Hz, 2H), 6.45 (s, 1H), 5.74 (d, J = 17.5 Hz, 1H), 5.56 (dd, J = 8.0, 4.5 Hz, 1H), 5.48 (d, J = 17.5 Hz, 1H), 3.52 (dd, J = 14.2, 4.5 Hz, 1H), 3.25 (dd, J = 14.2, 8.1 Hz, 1H), 2.30 (s, 3H), 2.14 (s, 3H).
^{13}C NMR (101 MHz, $CDCl_3$) δ 167.8, 146.3, 140.3, 138.2, 138.1, 133.9, 133.6, 133.0, 132.1, 129.2, 128.8, 128.7, 128.5, 125.1, 120.1, 111.2, 57.1, 44.7, 44.0, 20.5, 20.2.

HRMS (ESI, m/z): calculated for $C_{25}H_{24}O_3N_3S$ $[M+H]^+$: 446.15329, found: 446.15283.
HPLC analysis: 99.6:0.4 e.r. (Chiralpak IA, Hexane/2-PrOH = 70/30, 1.0 mL/min), Rt (major) = 20.3 min, Rt (minor) = 15.7 min.

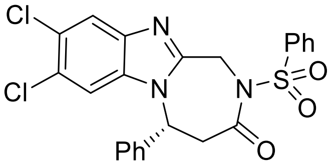

3k

(*R*)-8,9-dichloro-5-phenyl-2-(phenylsulfonyl)-1,2,4,5-tetrahydro-3*H*-benzo[4,5]imidazo[1,2-a][1,4]diazepin-3-one (3k)

White solid, m.p. 138-139 ℃, 94% yield.
$[\alpha]^{25}_{D}$ = 1.0 (c = 0.5 in $CHCl_3$).
^1H NMR (500 MHz, $CDCl_3$) δ 7.90 (d, *J* = 8.2 Hz, 2H), 7.86 (s, 1H), 7.58 (t, *J* = 7.7 Hz, 1H), 7.41 (t, *J* = 8.0 Hz, 2H), 7.27 – 7.23 (m, 2H), 7.16 (t, *J* = 7.6 Hz, 2H), 6.79 (s, 1H), 6.73 (d, *J* = 7.4 Hz, 2H), 5.76 (d, *J* = 17.6 Hz, 1H), 5.57 (dd, *J* = 8.0, 4.5 Hz, 1H), 5.50 (d, *J* = 17.4 Hz, 1H), 3.56 (dd, *J* = 14.4, 4.5 Hz, 1H), 3.28 (dd, *J* = 14.4, 7.9 Hz, 1H).
^{13}C NMR (126 MHz, $CDCl_3$) δ 167.33, 149.46, 141.16, 138.09, 137.12, 134.29, 129.65, 129.21, 128.95, 128.89, 128.01, 127.59, 125.15, 121.55, 112.70, 57.70, 44.60, 43.86.
HRMS (ESI, m/z): calculated for $C_{23}H_{16}O_3N_3Cl_2S$ $[M-H]^+$: 484.02839, found: 484.02936.
HPLC analysis: 99.6:0.4 e.r. (Chiralpak IA, Hexane/2-PrOH = 70/30, 1.0 mL/min), Rt (major) = 22.7 min, Rt (minor) = 16.5 min.

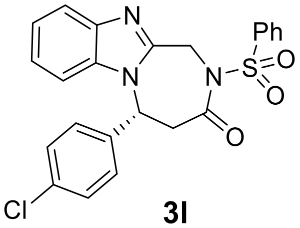

3l

(*R*)-5-(4-chlorophenyl)-2-(phenylsulfonyl)-1,2,4,5-tetrahydro-3*H*-benzo [4,5]imidazo[1,2-a][1,4]diazepin-3-one (3l)

White solid, m.p. 200-201 ℃, 98% yield.
$[\alpha]^{28}_{D}$ = -1559.1 (c 1.0 $CHCl_3$).
^1H NMR (400 MHz, $CDCl_3$) δ 7.93 (d, *J* = 47.4 Hz, 1H), 7.80 (d, *J* = 7.8 Hz, 2H), 7.56 (t, *J* = 7.5 Hz, 1H), 7.37 (t, *J* = 7.8 Hz, 2H), 7.32 – 7.26 (m, 1H), 7.13 (t, *J* = 7.7 Hz, 1H), 7.03 (d, *J* = 8.1 Hz, 2H), 6.76 (d, *J* = 8.1 Hz, 1H), 6.69 (d, *J* = 8.2 Hz, 2H), 5.94 (d, *J* = 17.9 Hz, 1H), 5.69 (s, 1H), 5.39 (d, *J* = 17.8 Hz, 1H), 3.68 (d, *J* = 13.5 Hz, 1H), 3.17 (dd, *J* = 14.5, 6.6 Hz, 1H).
^{13}C NMR (101 MHz, $CDCl_3$) δ 167.6, 147.9, 141.1, 138.0, 135.8, 134.6, 134.4, 134.1, 129.4, 128.8, 128.7, 126.7, 124.1, 123.6, 120.1, 111.3, 56.6, 44.5, 43.3.
HRMS (ESI, m/z): Mass calcd for $C_{23}H_{19}O_3N_3ClS$ $[M+H]^+$, 452.0830; found 452.0815.
HPLC analysis: 99.4:0.6 e.r. (Chiralpak IA, Hexane/2-PrOH = 70/30, 1.0 mL/min), Rt (major) = 25.3 min, Rt (minor) = 16.9 min.

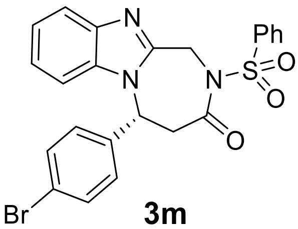

3m

(*R*)-5-(4-bromophenyl)-2-(phenylsulfonyl)-1,2,4,5-tetrahydro-3*H*-benzo[4,5]imidazo [1,2-a][1,4]diazepin-3-one (3m)

White solid, m.p. 170-172 ℃, 98% yield.

$[\alpha]^{28}_{D}$ = 67.87 (c 1.0 $CHCl_3$).

^1H NMR (500 MHz, $CDCl_3$) δ 7.88 (d, *J* = 7.8 Hz, 2H), 7.78 (d, *J* = 8.1 Hz, 1H), 7.56 (t, *J* = 7.5 Hz, 1H), 7.37 (t, *J* = 7.8 Hz, 2H), 7.27 – 7.23 (m, 1H), 7.19 (d, *J* = 8.1 Hz, 2H), 7.09 (t, *J* = 7.7 Hz, 1H), 6.69 (d, *J* = 8.1 Hz, 1H), 6.54 (d, *J* = 8.0 Hz, 2H), 5.82 (d, *J* = 17.6 Hz, 1H), 5.60 (dd, *J* = 7.2, 4.3 Hz, 1H), 5.38 (d, *J* = 17.5 Hz, 1H), 3.61 (dd, *J* = 14.4, 4.3 Hz, 1H), 3.18 (dd, *J* = 14.3, 7.2 Hz, 1H).

^{13}C NMR (101 MHz, $CDCl_3$) δ 167.4, 147.2, 141.7, 138.1, 136.7, 134.8, 134.0, 132.4, 128.8, 128.8, 126.8, 123.8, 123.2, 122.6, 120.3, 111.0, 56.5, 44.6, 43.4.

HRMS (ESI, m/z): calculated for $C_{23}H_{19}O_3N_3BrS$ $[M+H]^+$: 496.0325, found: 496.0307.

HPLC analysis: 99.1:0.9 e.r. (Chiralpak IA, Hexane/2-PrOH = 70/30, 1.0 mL/min), Rt (major) = 23.6 min, Rt (minor) = 16.8 min.

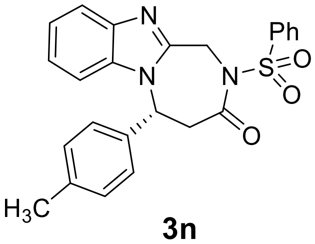

3n

(*R*)-2-(phenylsulfonyl)-5-(p-tolyl)-1,2,4,5-tetrahydro-3*H*-benzo[4,5]imidazo[1,2-a][1,4] diazepin-3-one (3n)

White solid, m.p. 208-209 ℃, 98% yield.

$[\alpha]^{28}_{D}$ = -650.4 (c 1.0 $CHCl_3$).

^1H NMR (500 MHz, $CDCl_3$) δ 7.87 (d, *J* = 6.4 Hz, 3H), 7.56 – 7.50 (m, 1H), 7.35 (t, *J* = 8.0 Hz, 2H), 7.28 – 7.21 (m, 1H), 7.08 (t, *J* = 7.7 Hz, 1H), 6.91 (d, *J* = 7.8 Hz, 2H), 6.75 (d, *J* = 8.2 Hz, 1H), 6.66 (d, *J* = 7.7 Hz, 2H), 5.80 (s, 1H), 5.60 (t, *J* = 6.0 Hz, 1H), 5.51 (d, *J* = 17.5 Hz, 1H), 3.61 – 3.44 (m, 1H), 3.26 (dd, *J* = 14.3, 7.9 Hz, 1H), 2.24 (s, 3H).

^{13}C NMR (101 MHz, $CDCl_3$) δ 167.8, 147.6, 141.6, 138.5, 138.3, 135.1, 134.9, 133.8, 129.9, 128.8, 128.7, 125.1, 123.7, 123.1, 120.1, 111.3, 57.2, 44.7, 44.0, 21.0.

HRMS (ESI, m/z): calculated for $C_{24}H_{22}O_3N_3S$ $[M+H]^+$: 432.1376, found: 432.1358.

HPLC analysis: 99.1:0.9 e.r. (Chiralpak IA, Hexane/2-PrOH = 70/30, 1.0 mL/min), Rt (major) = 19.6 min, Rt (minor) = 15.3 min.

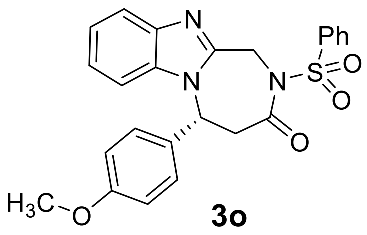

3o

(*R*)-5-(4-methoxyphenyl)-2-(phenylsulfonyl)-1,2,4,5-tetrahydro-3*H*-benzo[4,5]imidazo[1,2-a][1,4]diazepin-3-one (3o)

White solid, m.p. 174-175 ℃, 99% yield.

[α] $^{28}_{D}$ = -1097.3 (c 1.0 $CHCl_3$).

^{1}H NMR (500 MHz, $CDCl_3$) δ 7.91 (d, *J* = 7.3 Hz, 2H), 7.78 (d, *J* = 8.1 Hz, 1H), 7.54 (t, *J* = 7.8 Hz, 1H), 7.37 (t, *J* = 7.9 Hz, 2H), 7.26 – 7.19 (m, 1H), 7.10 – 7.04 (m, 1H), 6.74 (d, *J* = 8.2 Hz, 1H), 6.66 (d, *J* = 8.7 Hz, 2H), 6.62 (d, *J* = 8.9 Hz, 2H), 5.78 (d, *J* = 17.5 Hz, 1H), 5.58 (dd, *J* = 7.8, 4.4 Hz, 1H), 5.49 (d, *J* = 17.5 Hz, 1H), 3.71 (s, 3H), 3.53 (dd, *J* = 14.2, 4.4 Hz, 1H), 3.25 (dd, *J* = 14.2, 7.9 Hz, 1H).

^{13}C NMR (126 MHz, $CDCl_3$) δ 167.8, 159.5, 147.3, 141.7, 138.2, 135.0, 133.9, 129.9, 128.8, 128.7, 126.4, 123.6, 123.0, 120.1, 114.6, 111.4, 56.9, 55.2, 44.7, 44.1.

HRMS (ESI, m/z): calculated for $C_{24}H_{22}O_4N_3S$ [M+H]$^+$: 448.1325, found: 448.1309.

HPLC analysis: 99.4:0.6 e.r. (Chiralpak IA, Hexane/2-PrOH = 70/30, 1.0 mL/min), Rt (major) = 25.4 min, Rt (minor) = 19.6 min.

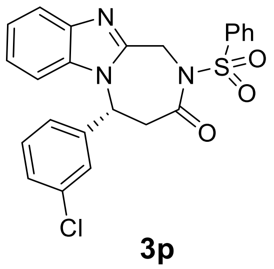

3p

(*R*)-5-(3-chlorophenyl)-2-(phenylsulfonyl)-1,2,4,5-tetrahydro-3*H*-benzo[4,5]imidazo [1,2-a][1,4]diazepin-3-one (3p)

White solid, m.p. 202-203 ℃, 94% yield.

[α] $^{28}_{D}$ = -305.7 (c 1.0 $CHCl_3$).

^{1}H NMR (500 MHz, $CDCl_3$) δ 7.90 (d, *J* = 7.8 Hz, 2H), 7.80 (d, *J* = 8.1 Hz, 1H), 7.53 (t, *J* = 7.4 Hz, 1H), 7.37 (t, *J* = 7.7 Hz, 2H), 7.28 – 7.24 (m, 1H), 7.21 (d, *J* = 8.0 Hz, 1H), 7.10 (t, *J* = 7.7 Hz, 1H), 7.00 (d, *J* = 12.1 Hz, 2H), 6.72 (d, *J* = 8.1 Hz, 1H), 6.47 (d, *J* = 7.8 Hz, 1H), 5.76 (d, *J* = 17.5 Hz, 1H), 5.60 (dd, *J* = 8.2, 4.5 Hz, 1H), 5.53 (d, *J* = 17.5 Hz, 1H), 3.54 (dd, *J* = 14.3, 4.5 Hz, 1H), 3.29 (dd, *J* = 14.3, 8.2 Hz, 1H).

^{13}C NMR (101 MHz, $CDCl_3$) δ 167.3, 147.2, 141.8, 140.0, 138.2, 135.3, 134.9, 134.0, 130.6, 129.0, 128.7, 128.7, 125.7, 123.9, 123.2, 120.4, 111.0, 56.8, 44.7, 43.7.

HRMS (ESI, m/z): calculated for $C_{23}H_{19}O_3N_3ClS$ [M+H]$^+$: 452.0830, found: 452.0815.

HPLC analysis: 98.5:1.5 e.r. (Chiralpak IA, Hexane/2-PrOH = 70/30, 1.0 mL/min), Rt (major) = 18.3 min, Rt (minor) = 15.0 min.

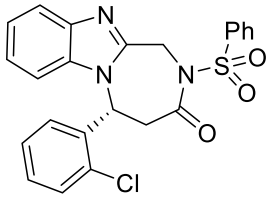

3q

(*R*)-5-(2-chlorophenyl)-2-(phenylsulfonyl)-1,2,4,5-tetrahydro-3*H*-benzo[4,5]imidazo [1,2-a][1,4]diazepin-3-one (3q)

White solid, m.p. 218-219 ℃, 95% yield.

[α] $^{28}{}_{D}$ = -420.4 (c 1.0 $CHCl_3$).

^{1}H NMR (500 MHz, CDCl3) δ 7.96 (d, *J* = 7.3 Hz, 2H), 7.81 (d, *J* = 8.1 Hz, 1H), 7.59 (t, *J* = 7.2 Hz, 1H), 7.43 (d, *J* = 4.0 Hz, 1H), 7.43 – 7.39 (m, 2H), 7.30 – 7.25 (m, 1H), 7.21 – 7.14 (m, 1H), 7.12 (t, *J* = 7.7 Hz, 1H), 6.69 (d, *J* = 8.1 Hz, 2H), 6.10 (s, 1H), 6.04 (s, 1H), 5.93 (d, *J* = 17.6 Hz, 1H), 5.42 (d, *J* = 17.5 Hz, 1H), 3.68 (d, *J* = 14.2 Hz, 1H), 3.33 (s, 1H).

^{13}C NMR (126 MHz, $CDCl_3$) *δ* 167.5, 147.5, 141.7, 138.3, 134.6, 134.0, 131.3, 130.1, 129.9, 128.8, 128.8, 127.7, 123.9, 123.2, 120.2, 110.7, 44.7, 40.5.

HRMS (ESI, m/z): calculated for $C_{23}H_{19}O_3N_3ClS$ $[M+H]^+$: 452.08302, found: 452.0810.

HPLC analysis: 99.8: 0.2 e.r. (Chiralpak IA, Hexane/2-PrOH = 70/30, 1.0 mL/min), Rt (major) = 29.4 min, Rt (minor) = 16.8 min.

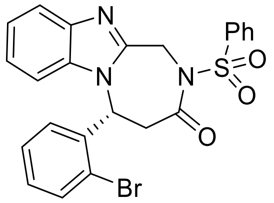

3r

(*R*)-5-(2-bromophenyl)-2-(phenylsulfonyl)-1,2,4,5-tetrahydro-3*H*-benzo[4,5]imidazo [1,2-a][1,4]diazepin-3-one (3r)

White solid, m.p. 226-227 ℃, 90% yield.

[α] $^{28}{}_{D}$ = 9161.5 (c 1.0 $CHCl_3$).

^{1}H NMR (400 MHz, $CDCl_3$) δ 8.13 (d, *J* = 8.2 Hz, 1H), 8.01 (d, *J* = 8.6 Hz, 2H), 7.83 (d, *J* = 8.1 Hz, 1H), 7.65 (t, *J* = 7.5 Hz, 1H), 7.49 (t, *J* = 7.9 Hz, 2H), 7.46 – 7.38 (m, 1H), 7.30 (t, *J* = 7.7 Hz, 1H), 7.17 – 7.11 (m, 1H), 7.06 (t, *J* = 7.7 Hz, 1H), 6.65 (d, *J* = 8.1 Hz, 1H), 6.51 (s, 1H), 6.22 (s, 1H), 6.09 (d, *J* = 17.6 Hz, 1H), 5.34 (d, *J* = 17.6 Hz, 1H), 3.91 (dd, *J* = 14.5, 4.5 Hz, 1H), 3.45 (d, *J* = 14.3 Hz, 1H).

^{13}C NMR (101 MHz, $CDCl_3$) δ 167.4, 147.6, 147.2, 141.8, 138.4, 134.5, 134.3, 134.0, 132.8, 129.7, 128.9, 128.8, 127.4, 125.6, 124.1, 123.5, 120.4, 110.2, 52.6, 44.8, 41.3.

HRMS (ESI, m/z): calculated for $C_{23}H_{19}O_3N_3BrS$ $[M+H]^+$: 496.03250, found: 496.03223.

HPLC analysis: 98.7: 1.3 e.r. (Chiralpak IA, Hexane/2-PrOH = 70/30, 1.0 mL/min), Rt (major) = 52.0 min, Rt (minor) = 26.6 min.

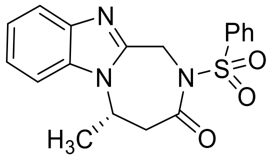

3s

(*R*)-5-methyl-2-(phenylsulfonyl)-1,2,4,5-tetrahydro-3*H*-benzo[4,5]imidazo[1,2-a][1,4] diazepin-3-one (3s)

White solid, m.p. 189-191 ℃, 98% yield.

[α] $^{28}{}_{D}$ = -835.4 (c 1.0 $CHCl_3$).

^{1}H NMR (500 MHz, $CDCl_3$) δ 7.90 (d, *J* = 7.2 Hz, 2H), 7.79 (d, *J* = 3.2 Hz, 1H), 7.53 (t, *J* = 7.5 Hz, 1H), 7.42 – 7.36 (m, 2H), 7.34 – 7.29 (m, 2H), 7.27 (d, *J* = 5.3 Hz, 1H), 5.81 (d, *J* = 17.5 Hz, 1H), 5.15 (d, *J* = 17.5 Hz, 1H), 4.73 (td, *J* = 6.5, 3.8 Hz, 1H), 3.50 (dd, *J* = 14.5, 3.7 Hz, 1H), 2.95 (dd, *J* = 14.5, 6.3 Hz, 1H), 1.27 (d, *J* = 6.6 Hz, 3H).

^{13}C NMR (101 MHz, $CDCl_3$) δ 168.9, 146.4, 141.9, 138.3, 134.6, 134.0, 128.7, 128.6, 123.5, 122.9, 120.4, 109.9, 48.2, 44.6, 41.9, 19.8.

HRMS (ESI, m/z): calculated for $C_{18}H_{18}O_3N_3S$ $[M+H]^+$: 356.1063, found: 356.1048.

HPLC analysis: 99.5: 0.4 e.r. (Chiralpak IA, Hexane/2-PrOH = 70/30, 1.0 mL/min), Rt (major) = 20.0 min, Rt (minor) = 16.4 min.

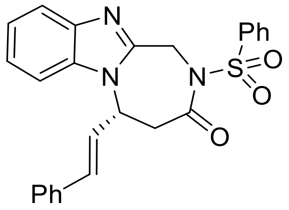

3t

(*R*, *E*)-2-(phenylsulfonyl)-5-styryl-1,2,4,5-tetrahydro-3*H*-benzo[4,5]imidazo[1,2-a][1,4]diazepin-3-one (3t)

White solid, m.p. 174-175 ℃, 81% yield.

[α] $^{28}{}_{D}$ = 4072.1 (c 1.0 $CHCl_3$).

^{1}H NMR (400 MHz, $CDCl_3$) δ 7.84 (d, *J* = 8.1 Hz, 3H), 7.36 (t, *J* = 7.1 Hz, 1H), 7.30 (t, *J* = 4.0 Hz, 1H), 7.26 (d, *J* = 2.2 Hz, 1H), 7.23 (t, *J* = 6.1 Hz, 6H), 7.03 (d, *J* = 2.6 Hz, 1H), 7.02 (d, *J* = 4.3 Hz, 1H), 6.21 (d, *J* = 15.8 Hz, 1H), 5.82 (d, *J* = 17.6 Hz, 1H), 5.75 (dd, *J* = 15.8, 6.8 Hz, 1H), 5.27 (d, *J* = 17.5 Hz, 2H), 3.54 (dd, *J* = 14.4, 3.9 Hz, 1H), 3.16 (dd, *J* = 14.4, 6.9 Hz, 1H).

^{13}C NMR (101 MHz, $CDCl_3$) δ 168.2, 146.7, 141.8, 138.2, 135.2, 134.9, 133.8, 133.3, 128.6, 128.6, 128.5, 128.4, 126.8, 124.8, 123.7, 123.1, 120.3, 111.0, 77.2, 54.8, 44.5, 41.8.

HRMS (ESI, m/z): calculated for $C_{25}H_{22}O_3N_3S$ $[M+H]^+$: 444.1376, found: 444.1358.

HPLC analysis: 98.4: 1.6 e.r. (Chiralcel AD-H, Hexane/2-PrOH = 80/20, 1 mL/min), Rt (major) = 25.0 min, Rt (minor) = 21.0 min.

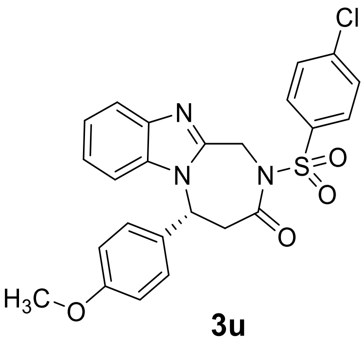

3u

(*R*)-2-((4-chlorophenyl)sulfonyl)-5-(4-methoxyphenyl)-1,2,4,5-tetrahydro-3*H*-benzo[4,5]imidazo[1,2-a][1,4]diazepin-3-one (3u)

White solid, m.p. 178-179 ℃, 99% yield.

[α] $^{28}{}_{D}$ = -130.9 (c 1.0 $CHCl_3$).

^{1}H NMR (400 MHz, $CDCl_3$) δ 7.85 (d, *J* = 8.7 Hz, 2H), 7.78 (d, *J* = 8.1 Hz, 1H), 7.34 (d, *J* = 8.8 Hz, 2H), 7.27 – 7.21 (m, 1H), 7.09 (t, *J* = 7.5 Hz, 1H), 6.75 (d, *J* = 8.1 Hz, 1H), 6.63 (s, 4H), 5.79 (d, *J* = 17.5 Hz, 1H), 5.61 (dd, *J* = 7.8, 4.4 Hz, 1H), 5.47 (d, *J* = 17.6 Hz, 1H), 3.73 (s, 3H), 3.57 (dd, *J* = 14.2, 4.4 Hz, 1H), 3.26 (dd, *J* = 14.2, 7.8 Hz, 1H).

^{13}C NMR (101 MHz, $CDCl_3$) δ 167.8, 159.6, 147.1, 141.8, 140.7, 136.7, 135.1, 130.4, 129.8, 129.0, 123.7, 123.1, 120.2, 114.6, 111.4, 56.9, 55.2, 44.8, 44.0.

HRMS (ESI, m/z): calculated for $C_{24}H_{21}O_4N_3ClS$ $[M+H]^+$: 482.0935, found: 482.0914.

HPLC analysis: 98.7:1.3 e.r. (Chiralpak IA, Hexane/2-PrOH = 70/30, 1.0 mL/min), Rt (major) = 35.0 min, Rt (minor) = 29.1 min.

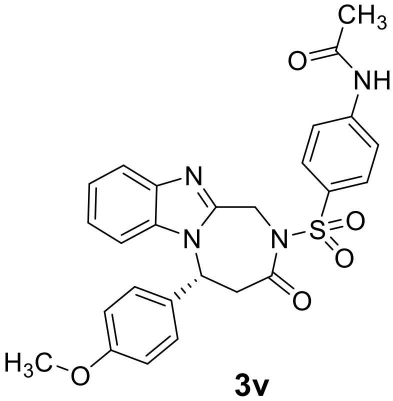

3v

(*R*)-N-(4-((5-(4-methoxyphenyl)-3-oxo-4,5-dihydro-1*H*-benzo[4,5]imidazo[1,2-a][1,4]diazepin-2(3*H*)-yl)sulfonyl)phenyl)acetamide (3v)

White solid, m.p. 208-209 ℃, 94% yield.

[α] $^{28}{}_{D}$ = 287.6 (c 1.0 $CHCl_3$).

^{1}H NMR (400 MHz, $CDCl_3$) δ 8.20 (s, 1H), 7.86 – 7.74 (m, 3H), 7.47 (d, *J* = 9.0 Hz, 2H), 7.27 (t, *J* = 7.7 Hz, 1H), 7.16 – 7.09 (m, 1H), 6.80 (d, *J* = 8.2 Hz, 1H), 6.72 (d, *J* = 8.8 Hz, 2H), 6.68 (d, *J* = 9.0 Hz, 2H), 5.86 (d, *J* = 17.5 Hz, 1H), 5.67 (dd, *J* = 7.4, 4.3 Hz, 1H), 5.48 (d, *J* = 17.5 Hz, 1H), 3.73 (s, 3H), 3.64 (dd, *J* = 14.3, 4.3 Hz, 1H), 3.27 (dd, *J* = 14.3, 7.5 Hz, 1H), 2.17 (s, 3H).

^{13}C NMR (101 MHz, $CDCl_3$) δ 168.7, 168.1, 159.6, 147.2, 143.3, 141.7, 135.1, 132.4, 130.1, 129.8, 126.5, 123.7, 123.1, 120.1, 118.7, 114.7, 111.4, 56.9, 55.1, 44.7, 44.2, 24.5.
HRMS (ESI, m/z): calculated for $C_{26}H_{25}O_5N_4S$ $[M+H]^+$: 505.1540, found: 505.1517.
HPLC analysis: 99.7:0.3 e.r. (Chiralpak IA, Hexane/2-PrOH = 70/30, 1.0 mL/min), Rt (major) = 20.7 min, Rt (minor) = 17.7 min.

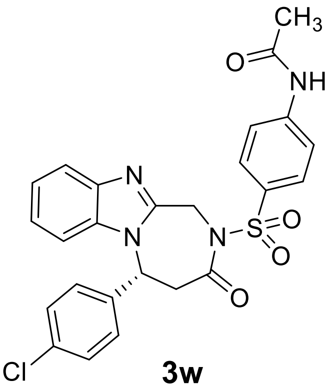

3w

(*R*)-N-(4-((5-(4-chlorophenyl)-3-oxo-4,5-dihydro-1*H*-benzo[4,5]imidazo[1,2-a][1,4] diazepin-2(3*H*)-yl)sulfonyl)phenyl)acetamide (3w)
White solid, m.p. 225-226 ℃, 91% yield.
$[\alpha]^{28}_D$ = 1616.8 (c 1.0 $CHCl_3$).
^1H NMR (500 MHz, $CDCl_3$) δ 8.71 (s, 1H), 7.72 (d, *J* = 8.6 Hz, 3H), 7.49 (d, *J* = 8.5 Hz, 2H), 7.25 (t, *J* = 7.7 Hz, 1H), 7.11 (d, *J* = 7.8 Hz, 1H), 7.07 (d, *J* = 8.3 Hz, 2H), 6.73 (d, *J* = 8.2 Hz, 1H), 6.67 (d, *J* = 8.1 Hz, 2H), 5.83 (d, *J* = 17.5 Hz, 1H), 5.67 (dd, *J* = 6.5, 4.2 Hz, 1H), 5.33 (d, *J* = 17.6 Hz, 1H), 3.67 (dd, *J* = 14.6, 4.1 Hz, 1H), 3.15 (dd, *J* = 14.5, 6.7 Hz, 1H), 2.14 (s, 3H).
^{13}C NMR (101 MHz, $CDCl_3$) δ 169.2, 167.9, 147.4, 143.6, 141.5, 136.1, 134.8, 134.4, 132.0, 130.1, 129.4, 126.7, 124.0, 123.4, 120.1, 118.8, 111.1, 56.4, 44.5, 43.4, 24.5.
HRMS (ESI, m/z): calculated for $C_{25}H_{22}O_4N_4ClS$ $[M+H]^+$: 509.1044, found: 509.1026.
HPLC analysis: 99.7: 0.3: e.r. (Chiralpak IA, Hexane/2-PrOH = 70/30, 1.0 mL/min), Rt (major) = 18.4 min, Rt (minor) = 13.7 min.

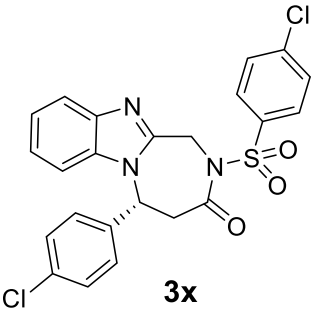

3x

(*R*)-5-(4-chlorophenyl)-2-((4-chlorophenyl)sulfonyl)-1,2,4,5-tetrahydro-3*H*-benzo [4,5]imidazo[1,2-a][1,4]diazepin-3-one (3x)
White solid, m.p. 161-163 ℃, 96% yield.
$[\alpha]^{28}_D$ = -587.3 (c 1.0 $CHCl_3$).

^{1}H NMR (500 MHz, DMSO-d_6) δ 7.85 (d, *J* = 8.7 Hz, 2H), 7.72 (d, *J* = 7.9 Hz, 2H), 7.65 (d, *J* = 8.7 Hz, 2H), 7.23 (t, *J* = 7.7 Hz, 1H), 7.19 (d, *J* = 7.4 Hz, 2H), 7.13 (t, *J* = 7.7 Hz, 1H), 6.90 (d, *J* = 8.1 Hz, 1H), 6.83 (d, *J* = 8.0 Hz, 2H), 6.11 (dd, *J* = 6.2, 4.0 Hz, 1H), 5.79 (d, *J* = 17.4 Hz, 1H), 5.62 (d, *J* = 17.4 Hz, 1H), 4.05 (d, *J* = 14.8 Hz, 1H), 3.21 (dd, *J* = 14.9, 6.2 Hz, 1H).
^{13}C NMR (101 MHz, DMSO-d_6) δ 169.4, 148.6, 141.8, 139.8, 137.9, 137.2, 135.2, 132.9, 130.6, 129.7, 129.0, 127.8, 123.6, 123.0, 119.9, 111.7, 55.5, 44.4, 42.8.
HRMS (ESI, m/z): calculated for $C_{23}H_{18}O_3N_3Cl_2S$ $[M+H]^+$: 486.0440, found: 486.0424.
HPLC analysis: 98.3: 1.7 e.r. (Chiralpak IA, Hexane/2-PrOH = 70/30, 1.0 mL/min), Rt (major) = 31.3 min, Rt (minor) = 15.0 min.

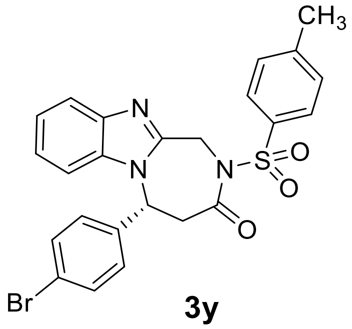

3y

(*R*)-5-(4-bromophenyl)-2-tosyl-1,2,4,5-tetrahydro-3*H*-benzo[4,5]imidazo[1,2-a][1,4] diazepin-3-one (3y)
White solid, m.p. 183-184 ℃, 98% yield.
$[\alpha]^{28}_D$ = 684.6 (c 1.0 $CHCl_3$).
^{1}H NMR (400 MHz, $CDCl_3$) δ 7.80 (d, *J* = 7.6 Hz, 3H), 7.28 (t, *J* = 7.7 Hz, 1H), 7.19 (d, *J* = 6.3 Hz, 4H), 7.12 (t, *J* = 7.7 Hz, 1H), 6.73 (d, *J* = 8.1 Hz, 1H), 6.57 (d, *J* = 8.1 Hz, 2H), 5.86 (d, *J* = 17.6 Hz, 1H), 5.64 (dd, *J* = 7.3, 4.2 Hz, 1H), 5.40 (d, *J* = 17.6 Hz, 1H), 3.63 (dd, *J* = 14.3, 4.3 Hz, 1H), 3.22 (dd, *J* = 14.3, 7.3 Hz, 1H), 2.41 (s, 3H).
^{13}C NMR (101 MHz, $CDCl_3$) δ 167.3, 147.3, 145.3, 141.8, 136.7, 135.1, 134.8, 132.3, 129.3, 128.9, 126.8, 123.8, 123.2, 122.5, 120.3, 110.9, 56.5, 44.5, 43.5, 21.6.
HRMS (ESI, m/z): calculated for $C_{24}H_{21}O_3N_3BrS$ $[M+H]^+$: 510.0481, found: 510.0467.
HPLC analysis: 99.2:0.8 e.r. (Chiralpak IA, Hexane/2-PrOH = 70/30, 1.0 mL/min), Rt (major) = 26.1 min, Rt (minor) = 16.2 min.

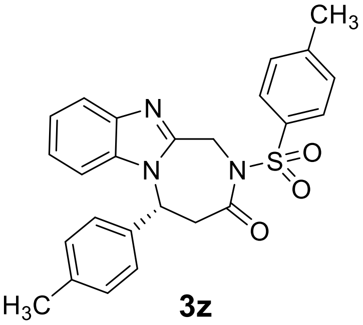

3z

(*R*)-5-(p-tolyl)-2-tosyl-1,2,4,5-tetrahydro-3*H*-benzo[4,5]imidazo[1,2-a][1,4]diazepin-3-one (3z)

White solid, m.p. 218-219 ℃, 98% yield.

[α] $^{28}{}_{D}$ = 107.5 (c 1.0 $CHCl_3$).

^{1}H NMR (500 MHz, Chloroform-*d*) δ 7.78 (d, *J* = 8.4 Hz, 3H), 7.23 (ddd, *J* = 8.2, 7.2, 1.1 Hz, 1H), 7.14 (d, *J* = 8.1 Hz, 2H), 7.06 (ddd, *J* = 8.3, 7.2, 1.1 Hz, 1H), 6.91 (d, *J* = 7.8 Hz, 2H), 6.77 – 6.71 (m, 1H), 6.65 (d, *J* = 7.8 Hz, 2H), 5.75 (d, *J* = 17.5 Hz, 1H), 5.59 (dd, *J* = 8.1, 4.4 Hz, 1H), 5.50 (d, *J* = 17.4 Hz, 1H), 3.50 (dd, *J* = 14.2, 4.4 Hz, 1H), 3.27 (dd, *J* = 14.2, 8.2 Hz, 1H), 2.35 (s, 3H), 2.25 (s, 3H).

^{13}C NMR (126 MHz, $CDCl_3$) δ 167.8, 147.4, 145.0, 141.8, 138.4, 135.3, 135.1, 135.0, 129.9, 129.3, 128.8, 125.1, 123.6, 122.9, 120.1, 111.3, 57.1, 44.7, 44.1, 21.6, 21.1.

HRMS (ESI, m/z): calculated for $C_{25}H_{24}O_3N_3S$ $[M+H]^+$: 446.1532, found: 446.1516.

HPLC analysis: 99.7:0.3 e.r. (Chiralpak IA, Hexane/2-PrOH = 70/30, 1.0 mL/min), Rt (major) = 19.6 min, Rt (minor) = 14.6 min.

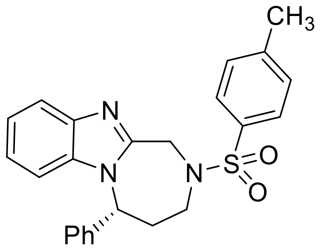

4

(*R*)-5-phenyl-2-tosyl-2,3,4,5-tetrahydro-1*H*-benzo[4,5]imidazo[1,2-a][1,4]diazepine (4)

White solid, m.p. 65-67 ℃, 61% yield.

[α] $^{25}{}_{D}$ = 12.3 (c = 0.5 in $CHCl_3$).

^{1}H NMR (500 MHz, $CDCl_3$) δ 7.70 (d, *J* = 7.8 Hz, 2H), 7.63 (d, *J* = 8.1 Hz, 1H), 7.22 (d, *J* = 5.5 Hz, 3H), 7.18 – 7.08 (m, 5H), 7.03 (d, *J* = 4.8 Hz, 2H), 5.93 (dd, *J* = 10.9, 4.4 Hz, 1H), 4.54 (dd, *J* = 14.6, 5.4 Hz, 1H), 4.29 (dd, *J* = 14.6, 2.9 Hz, 1H), 3.76 – 3.62 (m, 1H), 3.08 (t, *J* = 9.9 Hz, 1H), 2.66 (dd, *J* = 9.9, 4.8 Hz, 1H), 2.60 (dd, *J* = 14.9, 10.7 Hz, 1H), 2.31 (s, 3H).

^{13}C NMR (101 MHz, $CDCl_3$) δ 150.1, 143.5, 142.0, 138.3, 136.0, 133.5, 129.6, 128.7, 127.8, 127.2, 126.5, 123.0, 122.4, 119.7, 112.3, 57.7, 54.5, 40.2, 33.9, 21.4.

HRMS (ESI, m/z): calculated for $C_{24}H_{24}N_3O_3S$ $[M+OH]^+$: 434.15329, found: 434.15417.

HPLC analysis: 98.9: 1.1 e.r. (Chiralcel IA, Hexane/2-PrOH = 75/25, 1.5 mL/min), Rt (major) = 14.2 min, Rt (minor) = 6.7 min.

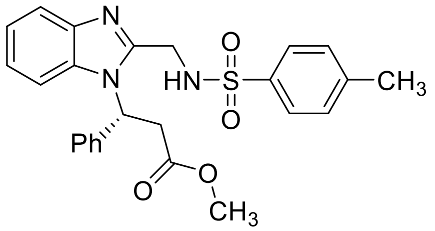

5

(*R*)-3-(2-(((4-methylphenyl)sulfonamido)methyl)-1*H*-benzo[d]imidazol-1-yl)-methyl-3-phenylpropanoate (5)

White solid, m.p. 68-70 ℃, 72% yield.

[α] $^{25}_{D}$ = 53.0 (c = 0.5 in $CHCl_3$).

^{1}H NMR (500 MHz, $CDCl_3$) δ 9.06 (d, *J* = 6.0 Hz, 1H), 7.77 (d, *J* = 8.1 Hz, 2H), 7.74 (d, *J* = 8.1 Hz, 1H), 7.21 (d, *J* = 5.7 Hz, 3H), 7.18 (d, *J* = 8.4 Hz, 4H), 7.13 (t, *J* = 7.7 Hz, 1H), 7.02 (t, *J* = 7.7 Hz, 1H), 6.95 (d, *J* = 8.2 Hz, 1H), 6.39 (dd, *J* = 10.4, 4.6 Hz, 1H), 4.92 (dd, *J* = 14.2, 9.0 Hz, 1H), 3.93 (d, *J* = 14.0 Hz, 1H), 3.54 (s, 3H), 3.53 – 3.44 (m, 2H), 2.38 (s, 3H).

^{13}C NMR (101 MHz, $CDCl_3$) δ 170.9, 150.3, 141.9, 136.9, 136.4, 133.2, 129.7, 129.0, 128.2, 127.4, 126.5, 123.3, 122.6, 120.1, 111.9, 54.2, 52.3, 40.7, 36.2, 21.7.

HRMS (ESI, m/z): calculated for $C_{25}H_{26}N_3O_4S$ $[M+H]^+$: 464.16385, found: 464.16293.

HPLC analysis: 99.1: 0.9 e.r. (Chiralcel IA, Hexane/2-PrOH = 75/25, 1.5 mL/min), Rt (major) = 23.0 min, Rt (minor) = 21.6 min.

IX. NMR spectra of products

3a: ^{1}H NMR

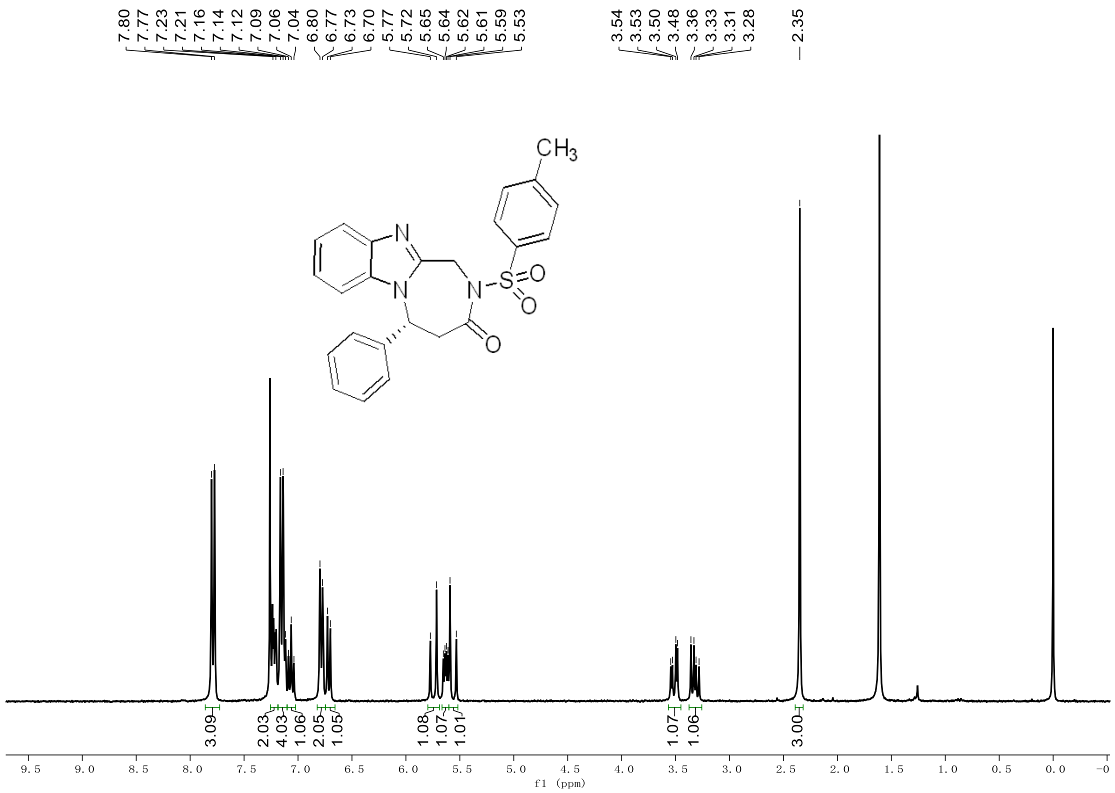

3a: ^{13}C NMR

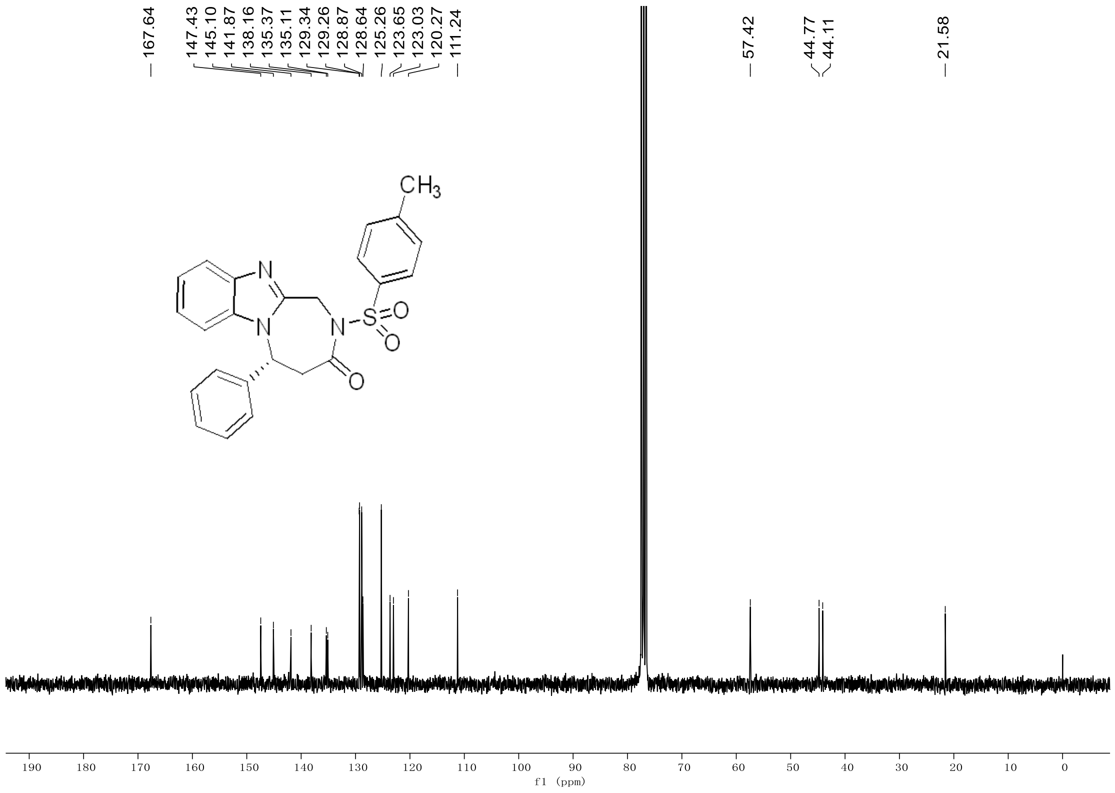

3b: ^{1}H NMR

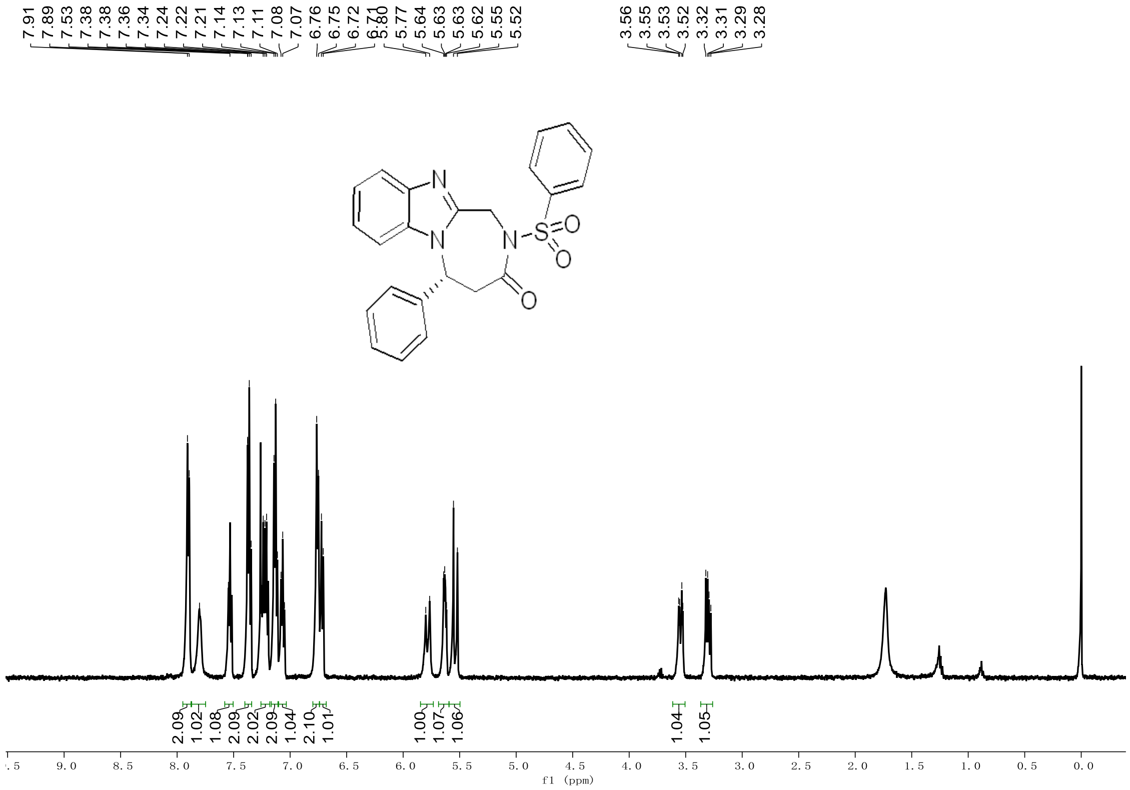

3b: ^{13}C NMR

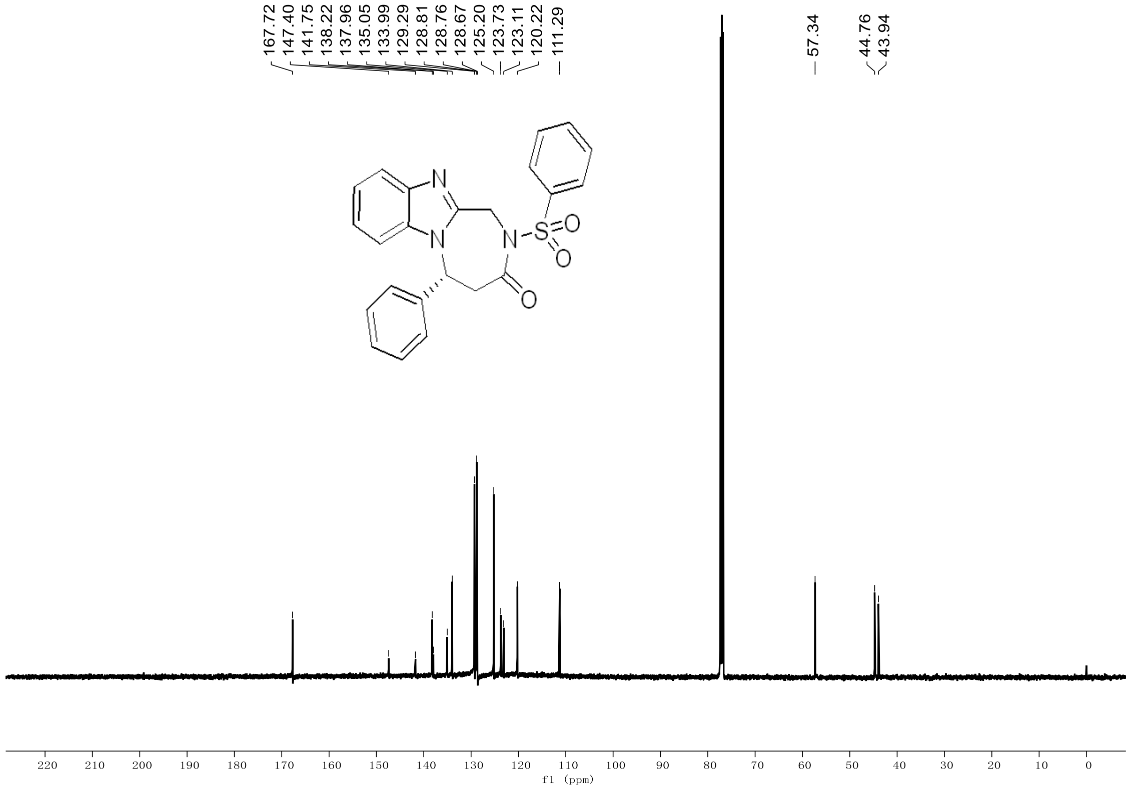

3c: ^{1}H NMR

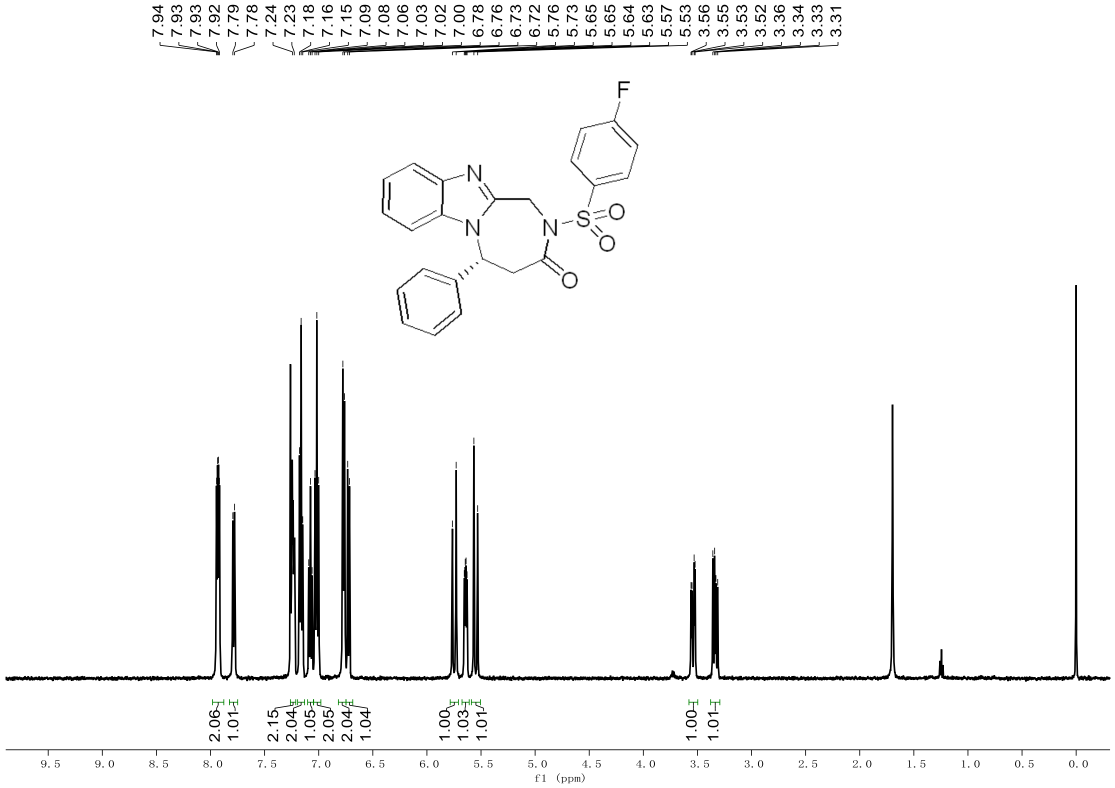

3c: ^{13}C NMR

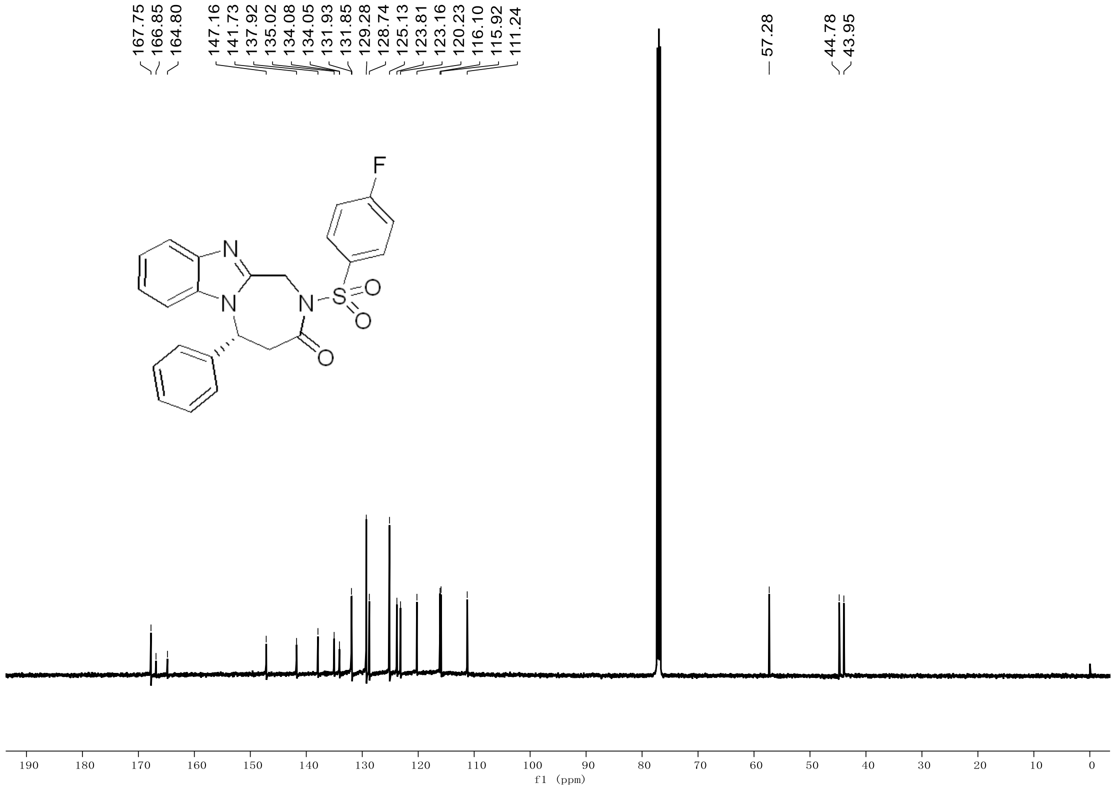

3c: ^{19}F NMR

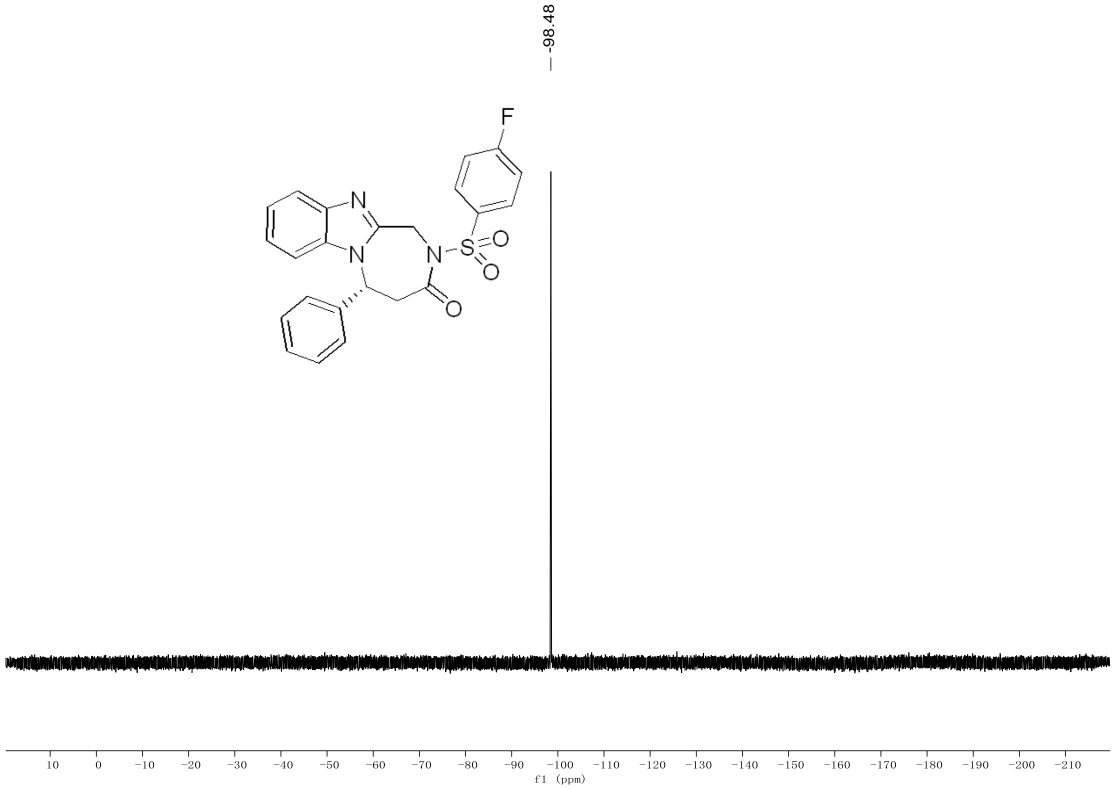

3d: ^{1}H NMR

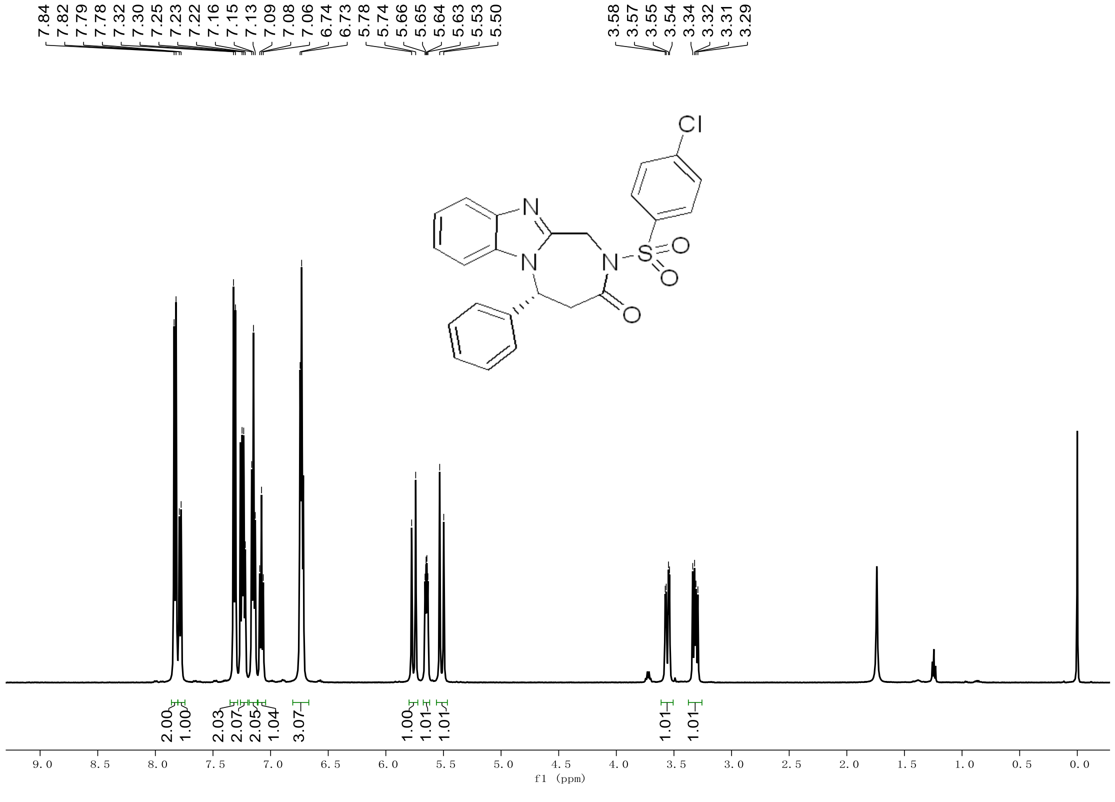

3d: ^{13}C NMR

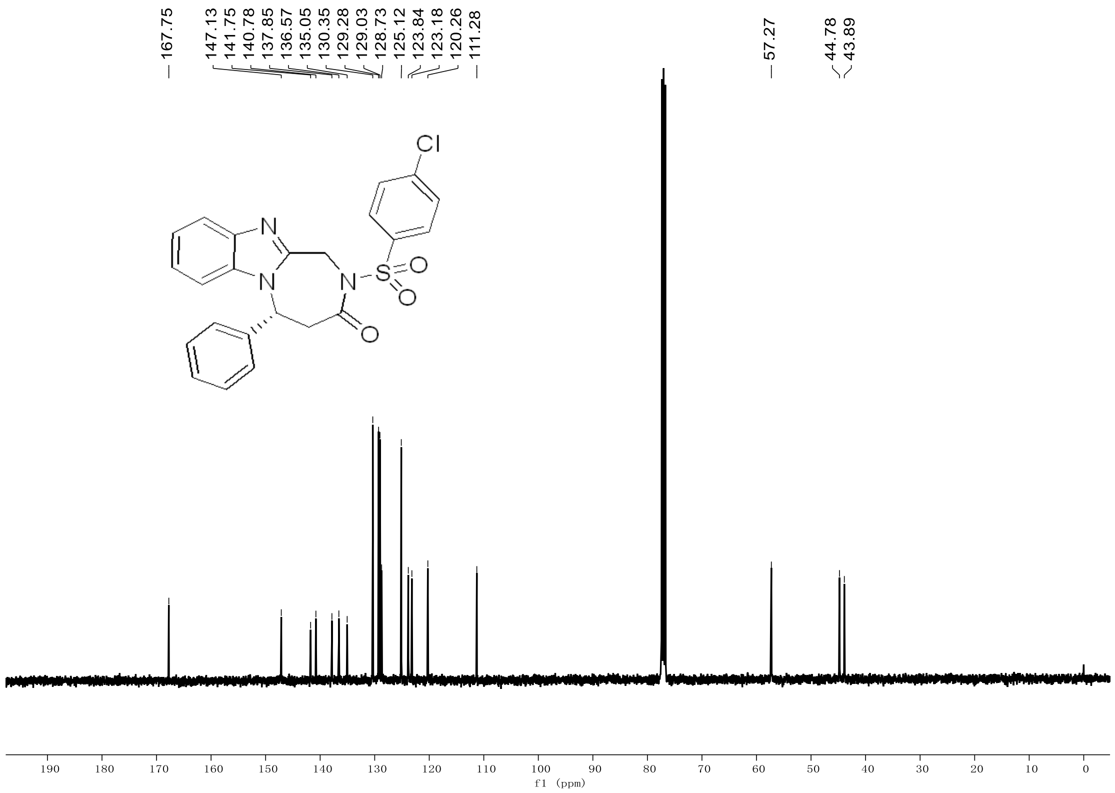

3e: ^{1}H NMR

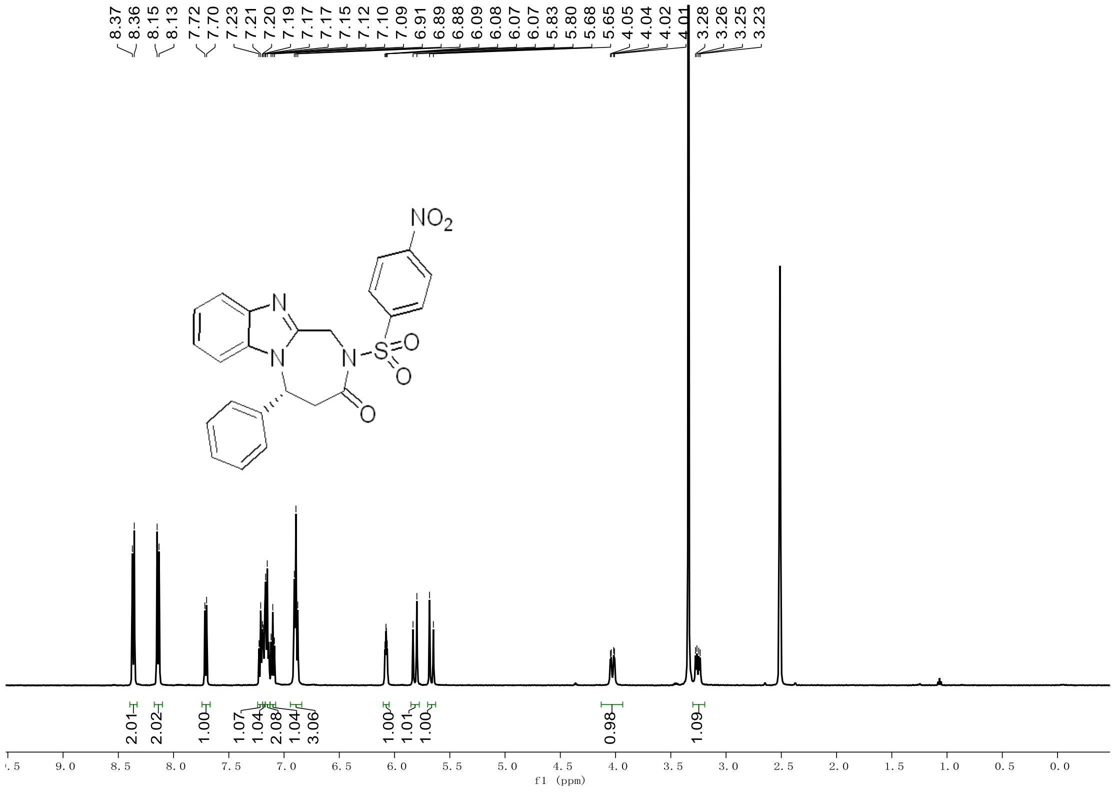

3e: ^{13}C NMR

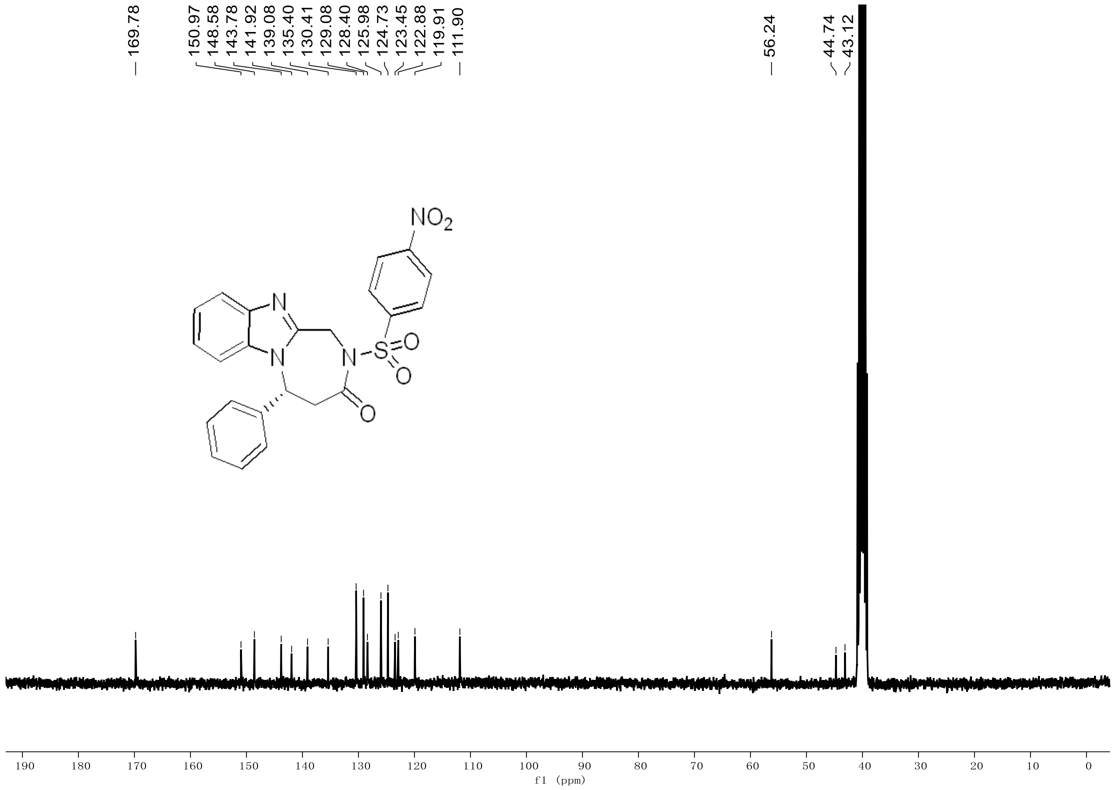

3f: ^{1}H NMR

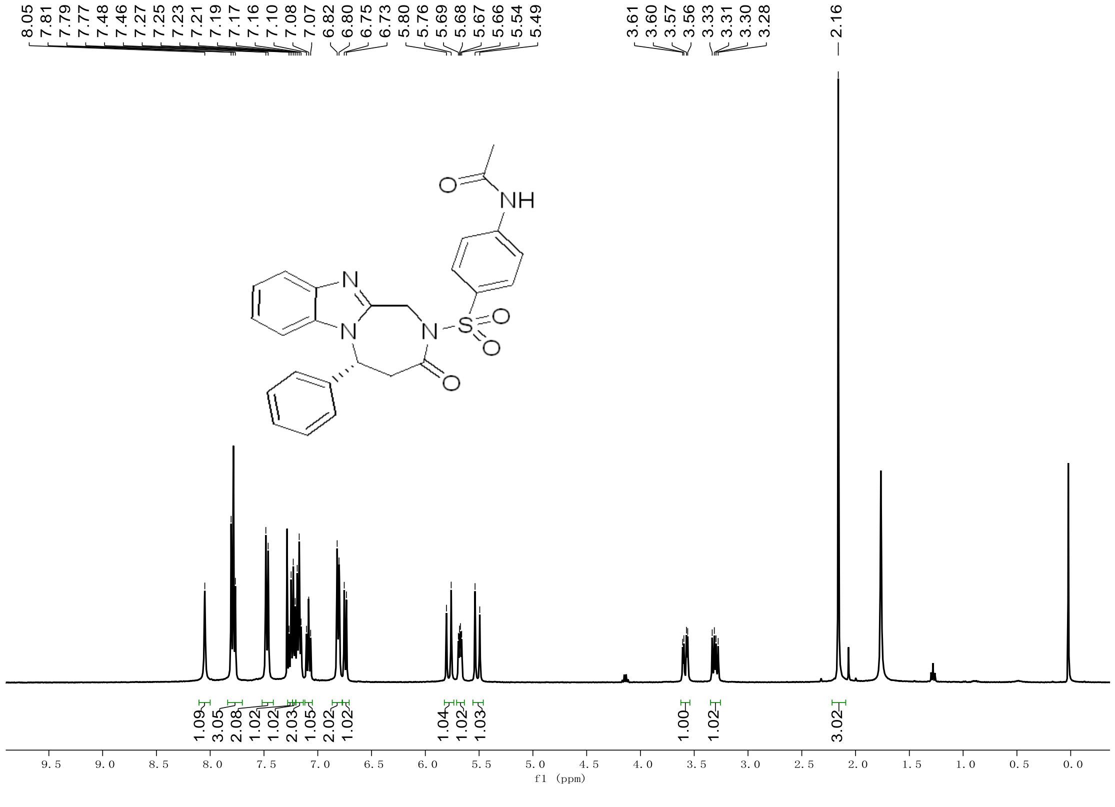

3f: ^{13}C NMR

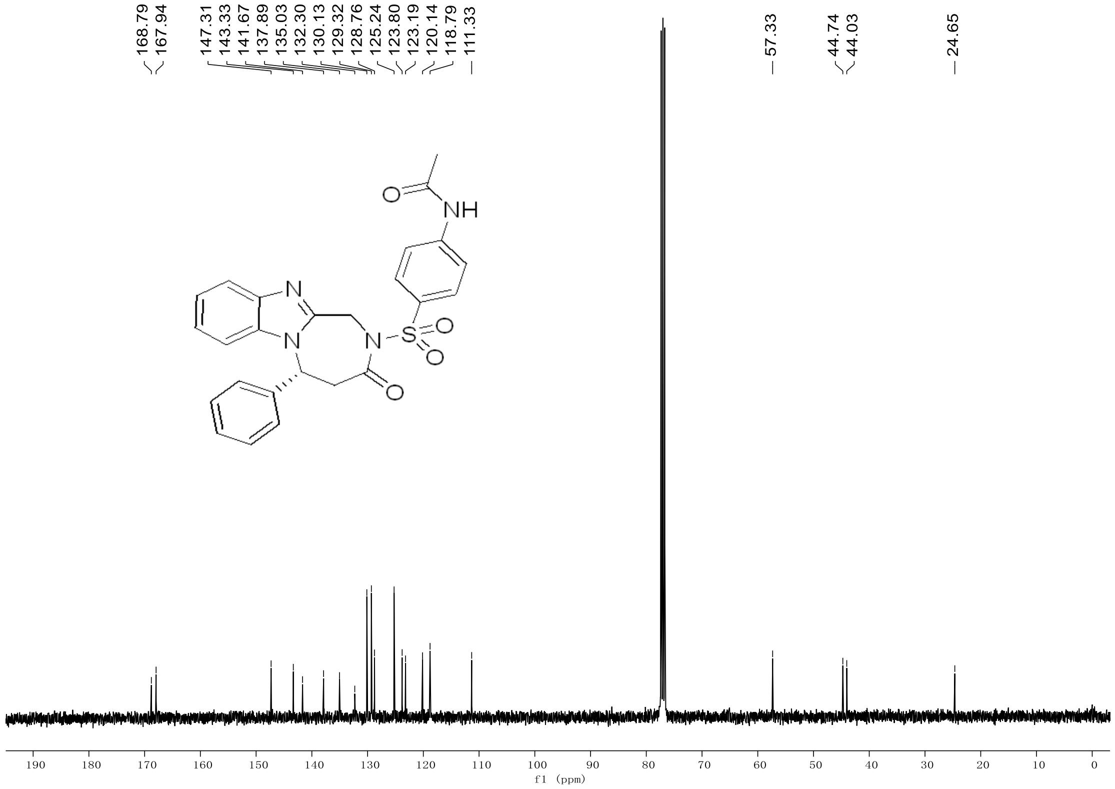

3g: ^{1}H NMR

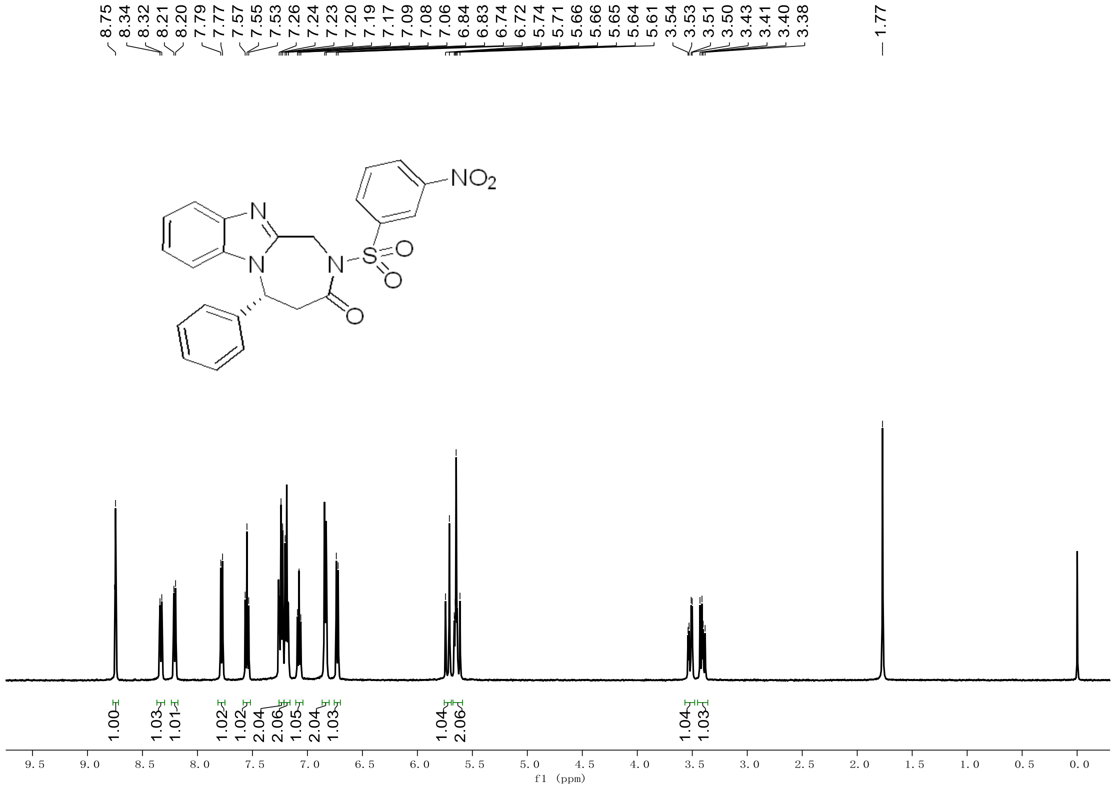

3g: ^{13}C NMR

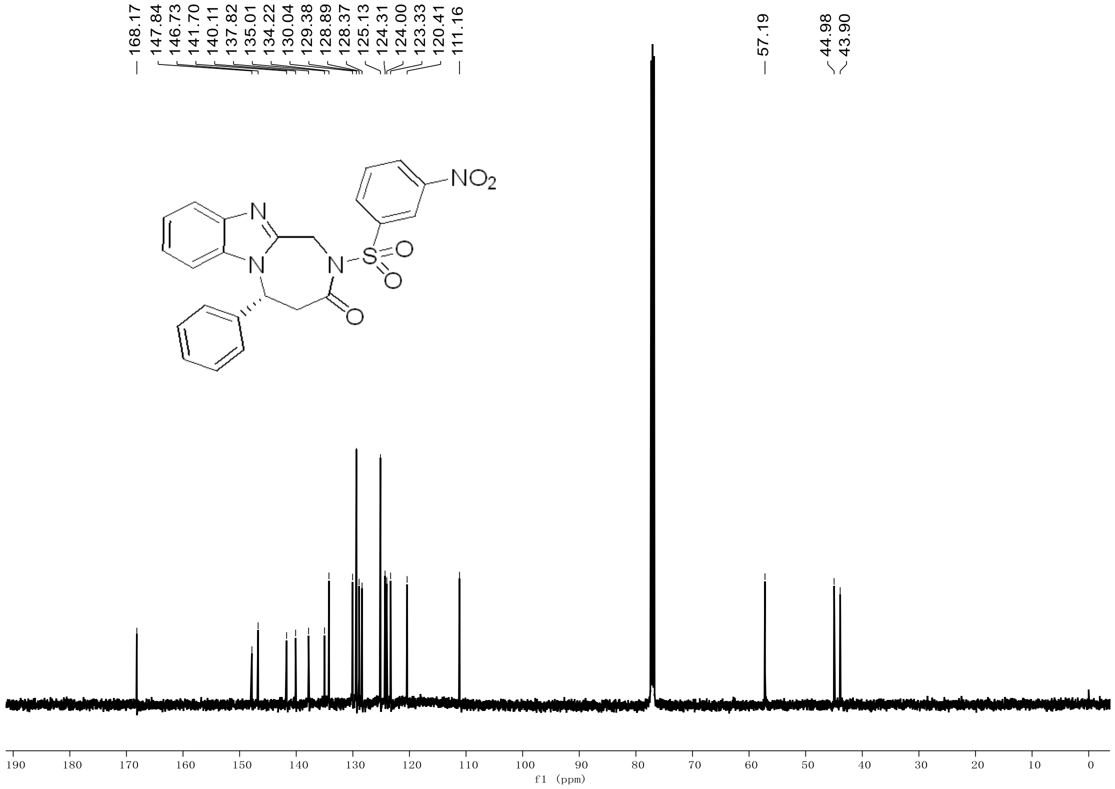

3h: ^{1}H NMR

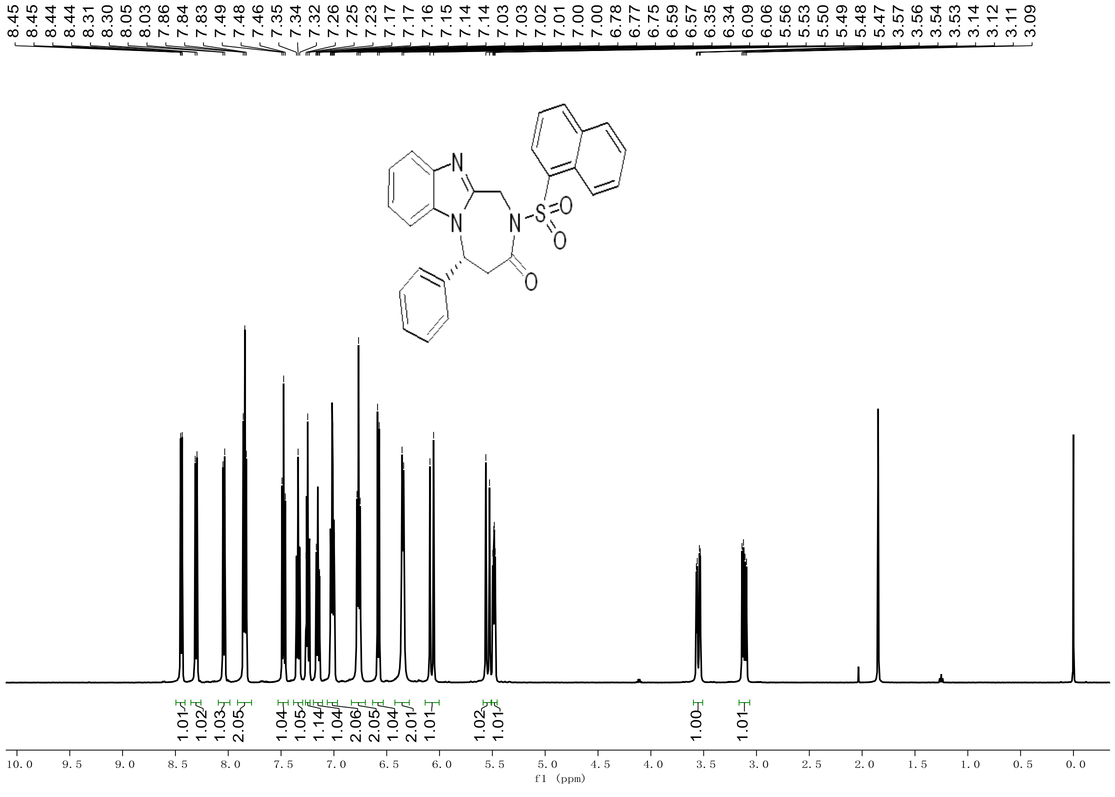

3h: ^{13}C NMR

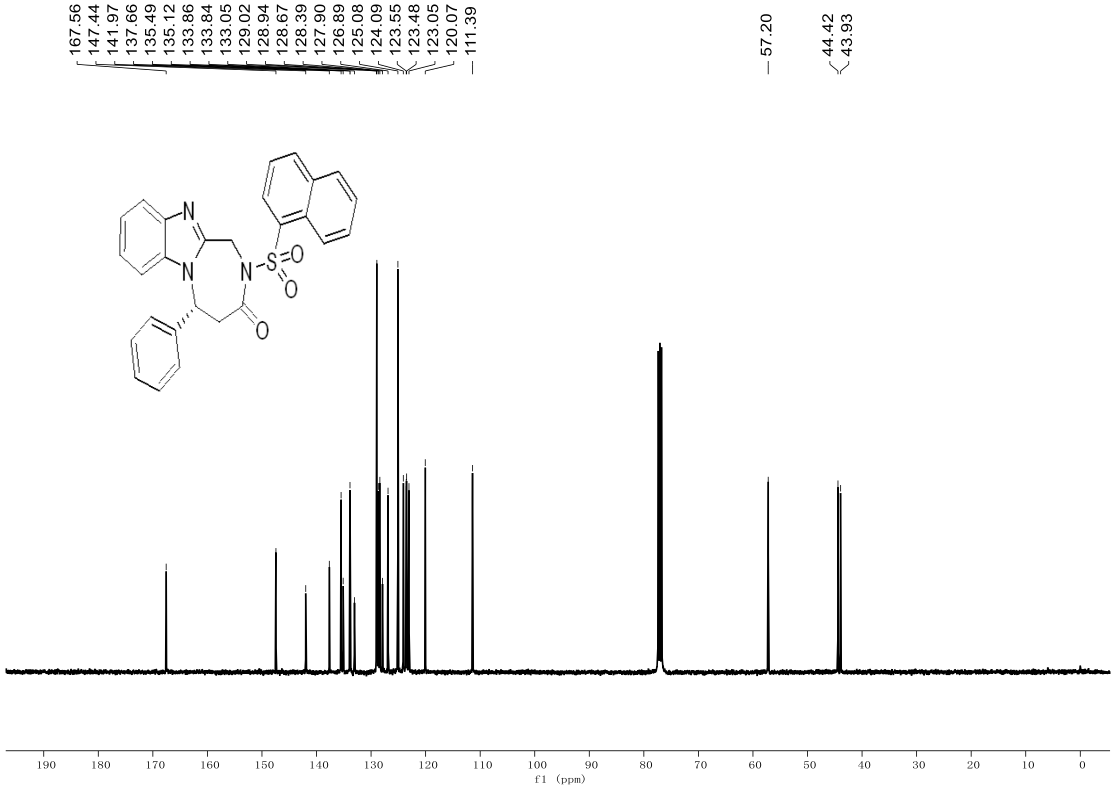

3i: ^{1}H NMR

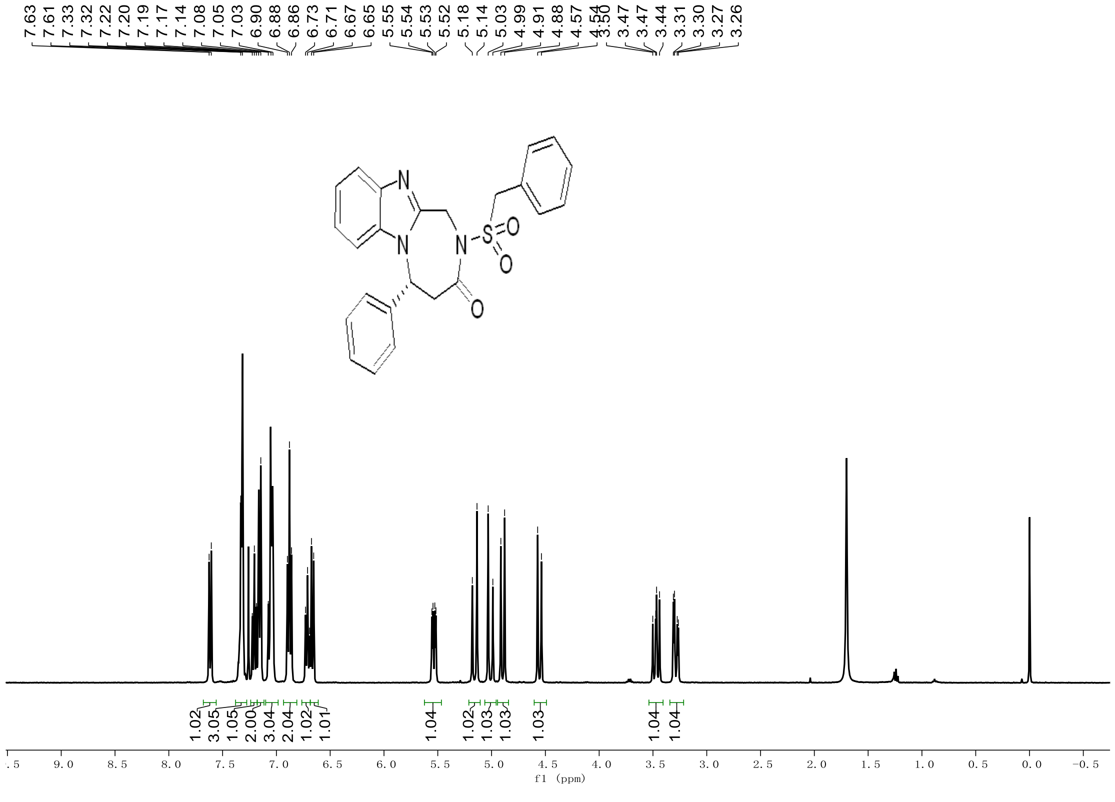

3i: ^{13}C NMR

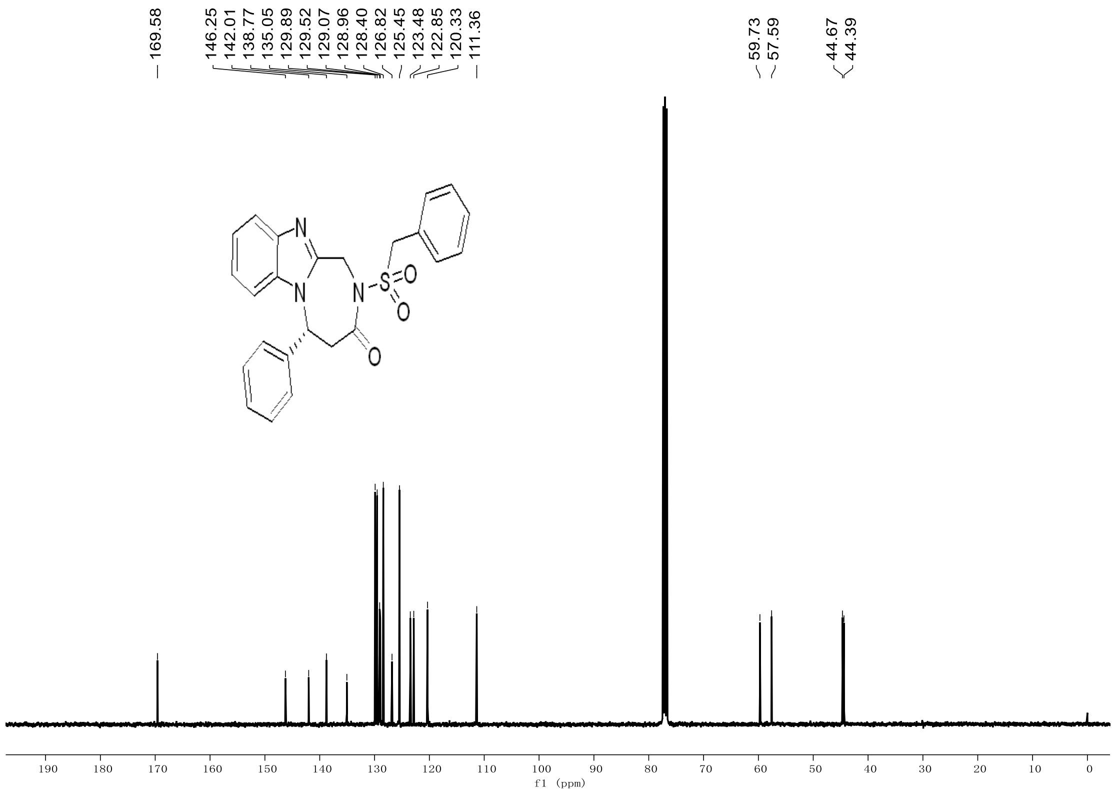

3j: ^{1}H NMR

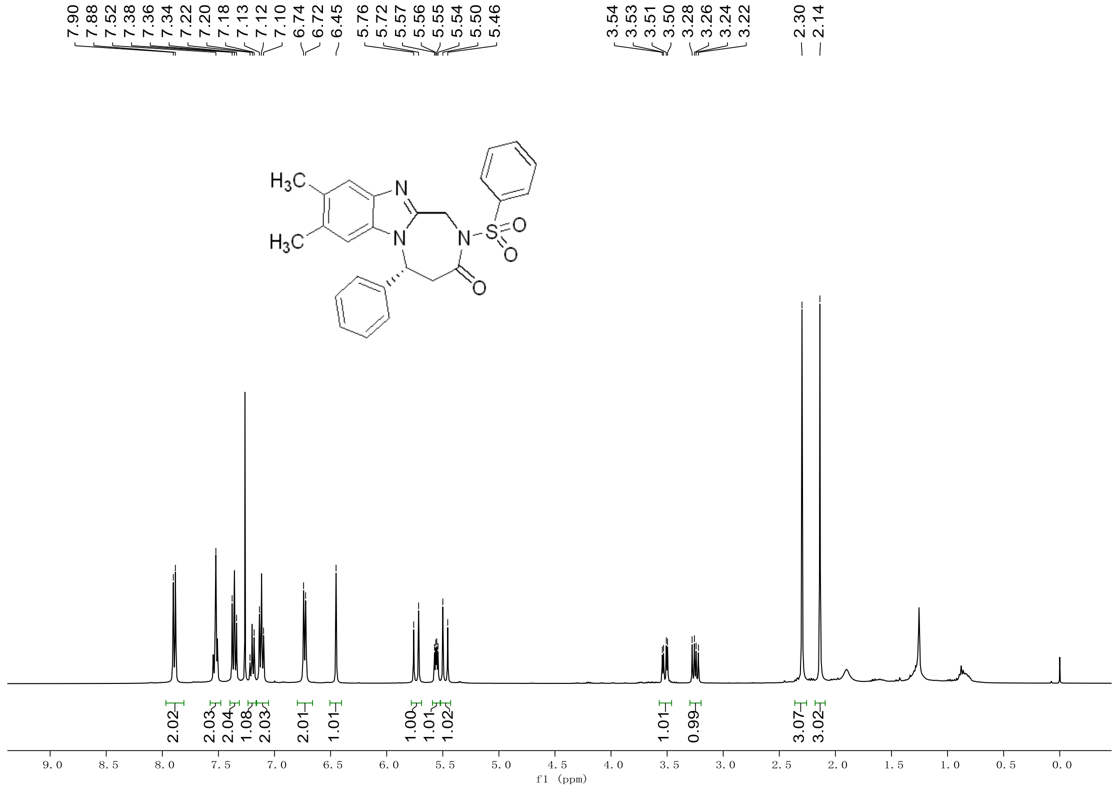

3j: ^{13}C NMR

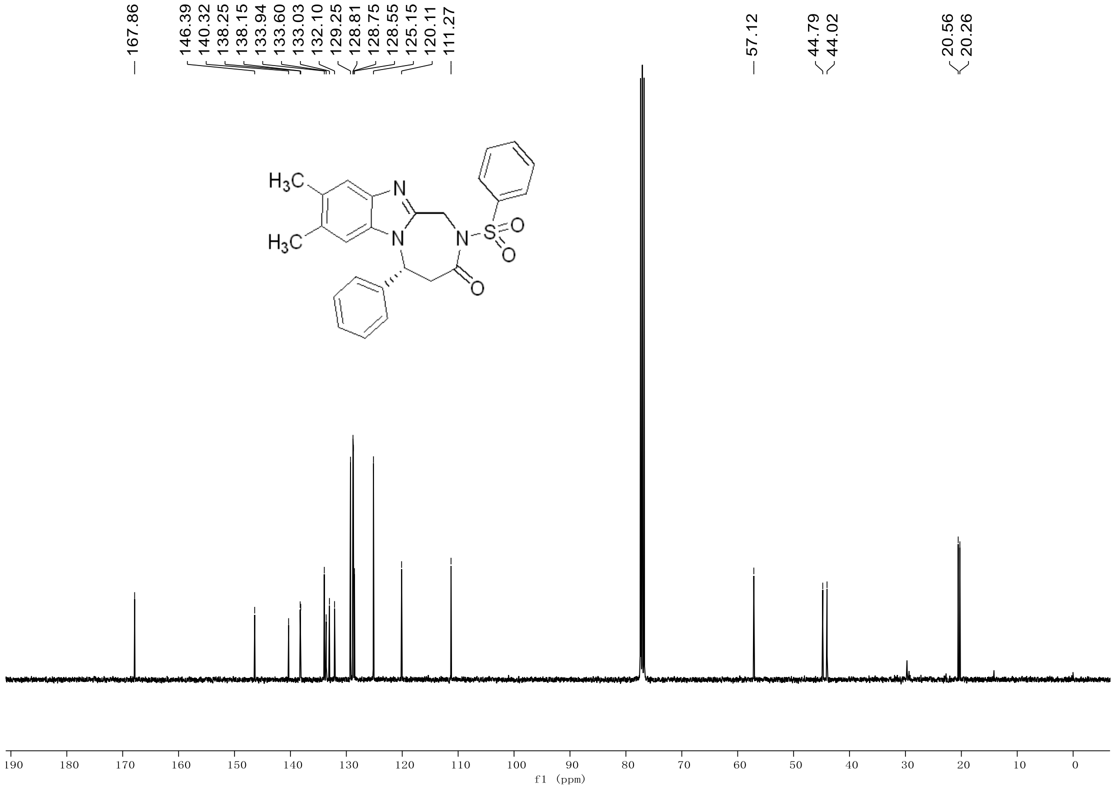

3k: ^1H NMR

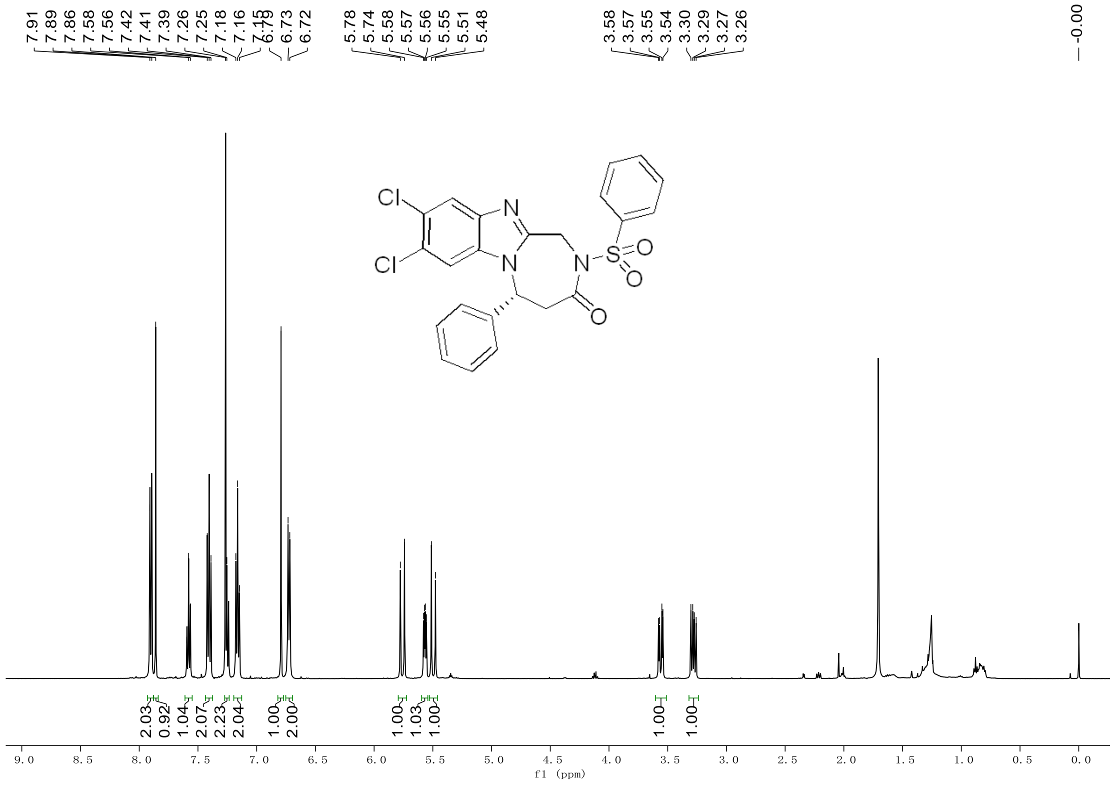

3k: ^{13}C NMR

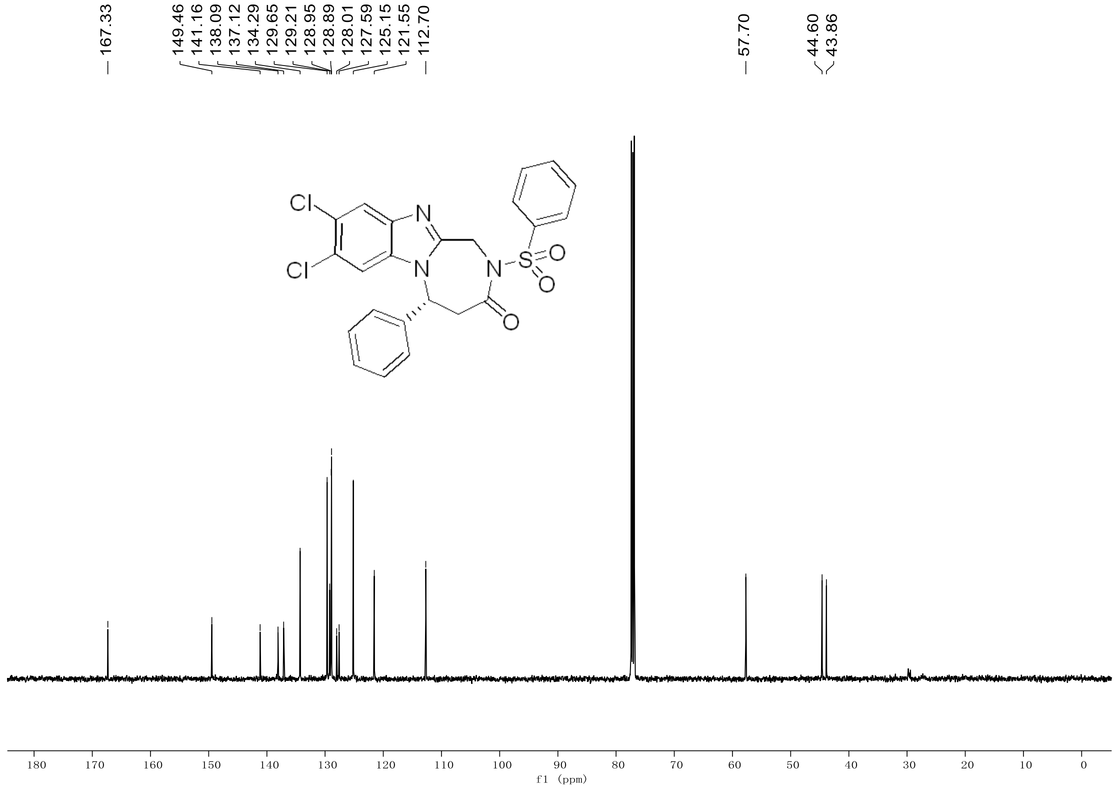

3l: ^{1}H NMR

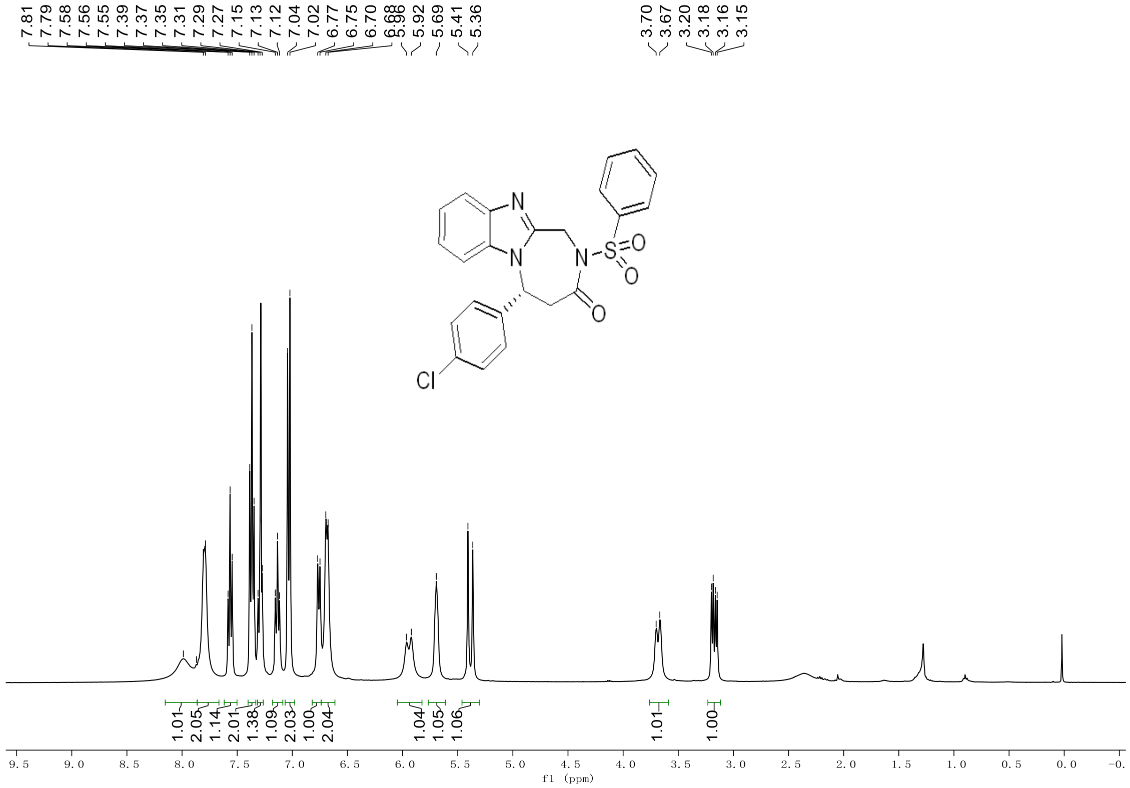

3l: ^{13}C NMR

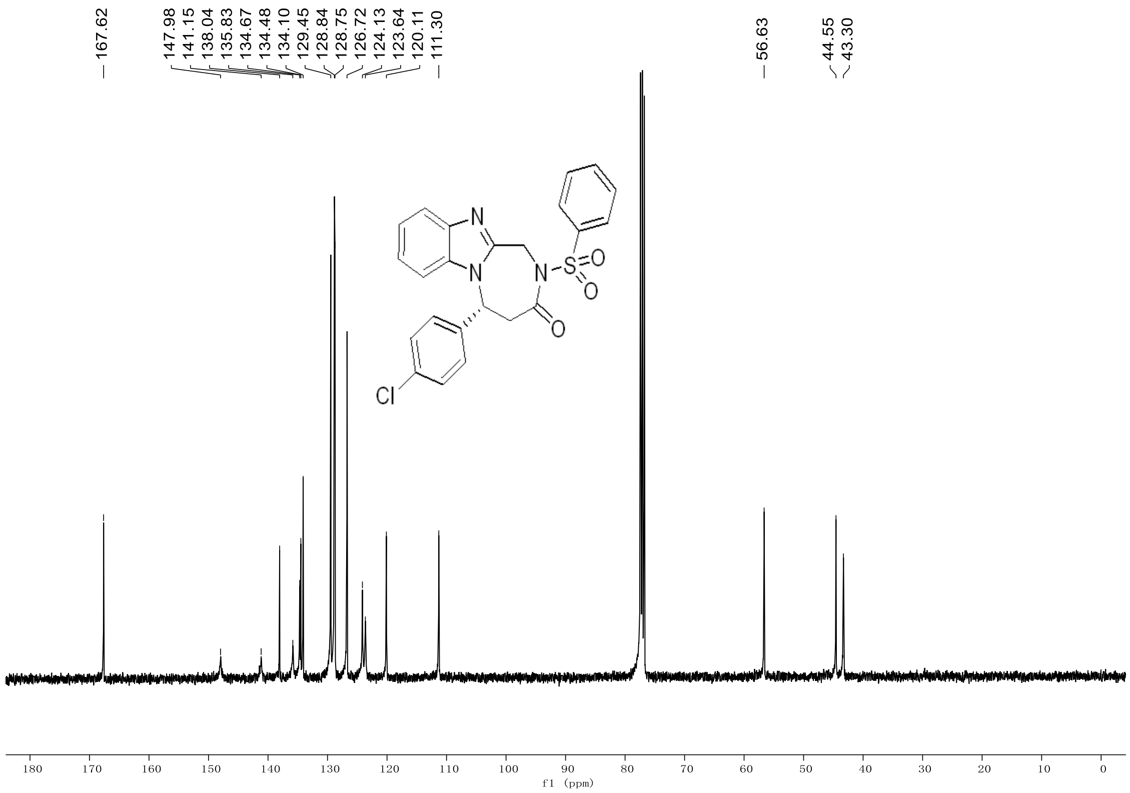

3m: ^{1}H NMR

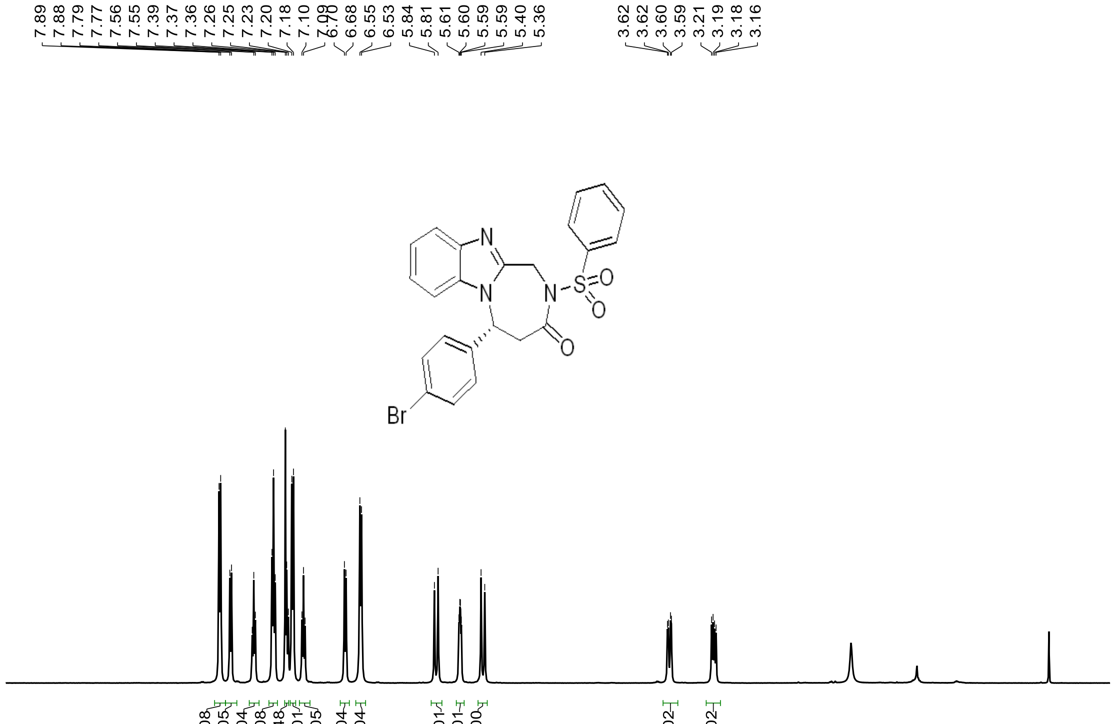

3m: ^{13}C NMR

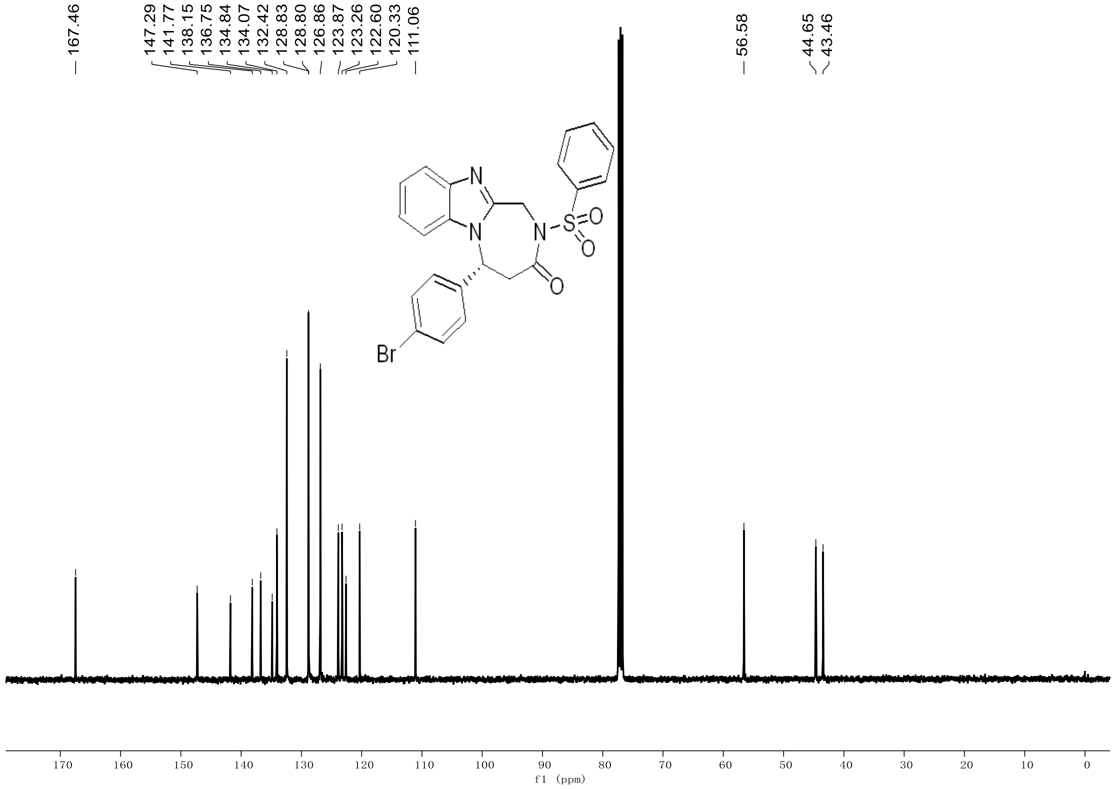

3n: ^{1}H NMR

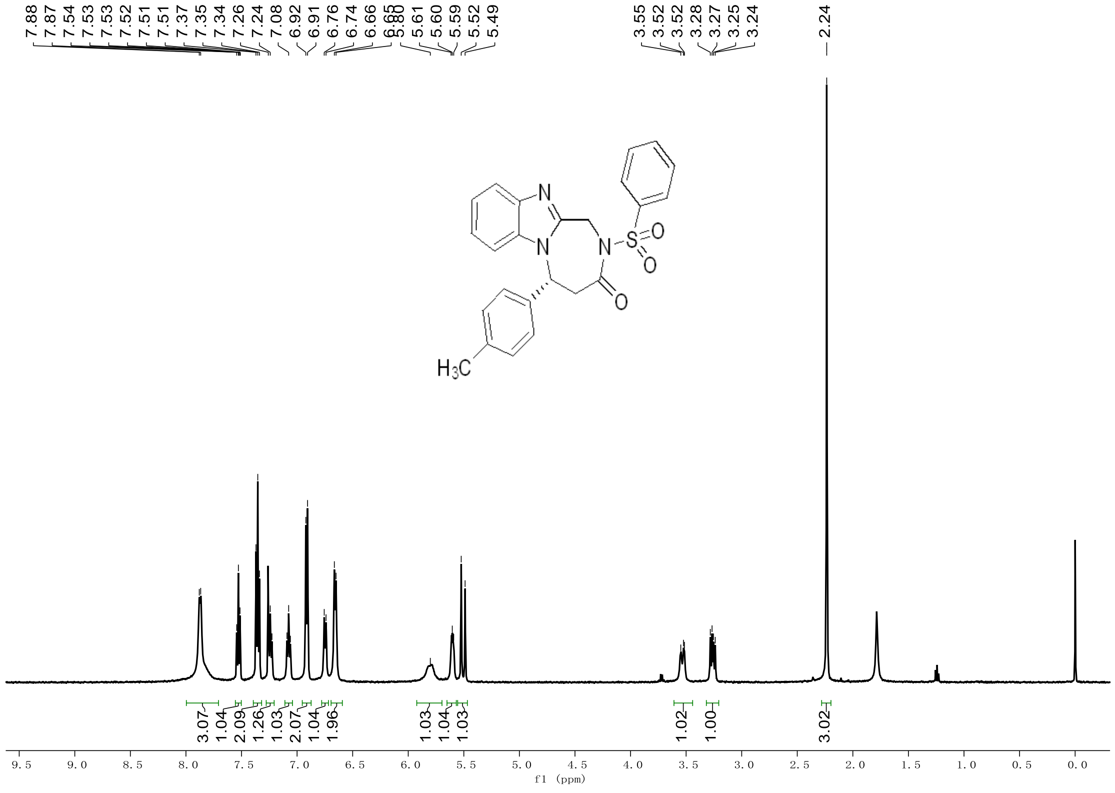

3n: ^{13}C NMR

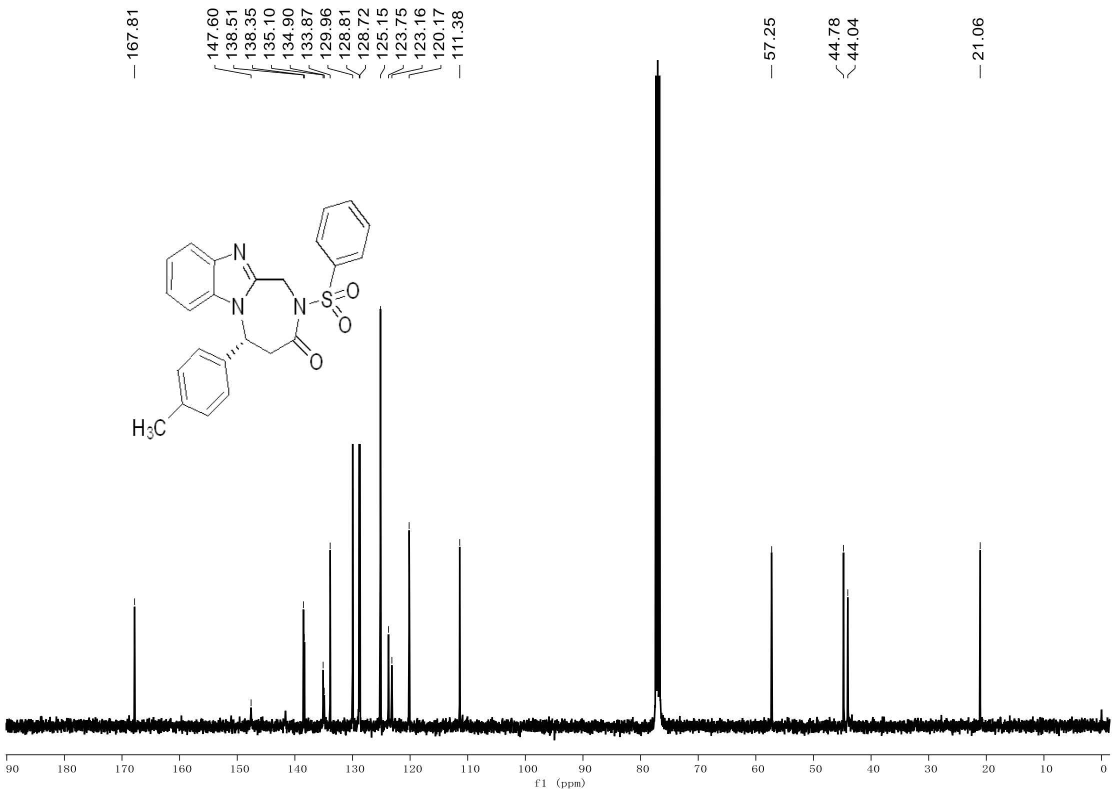

3o: ^{1}H NMR

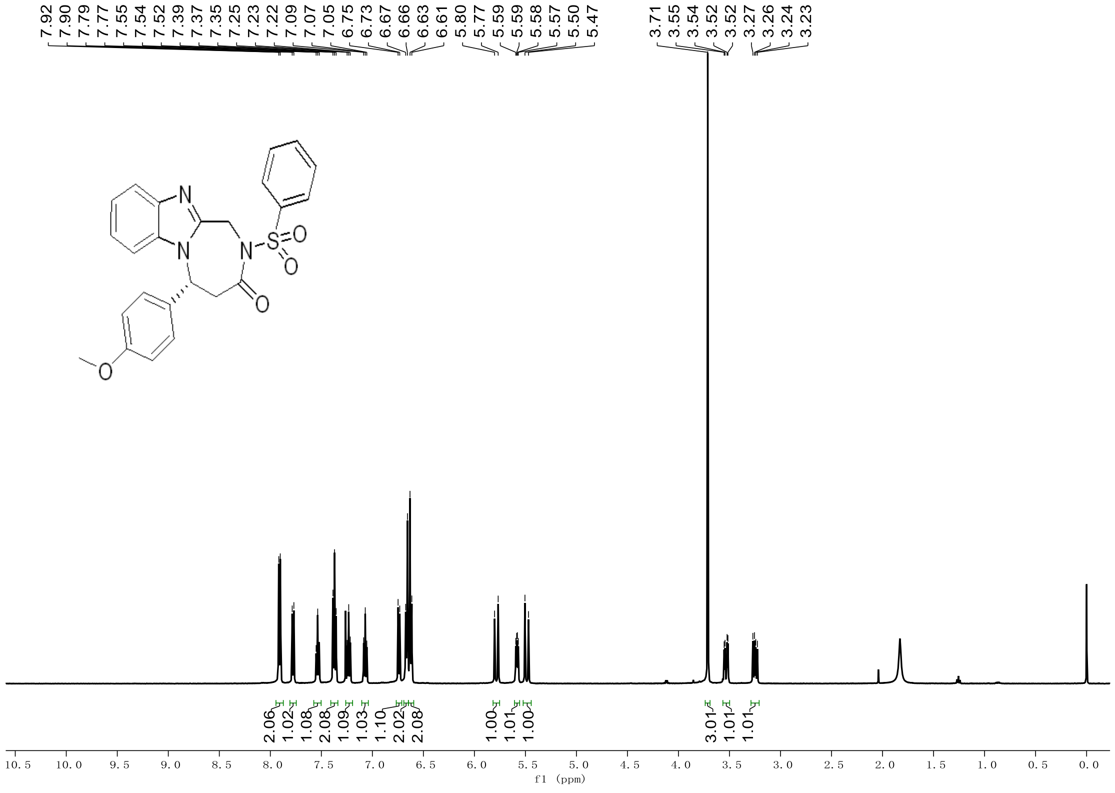

3o: ^{13}C NMR

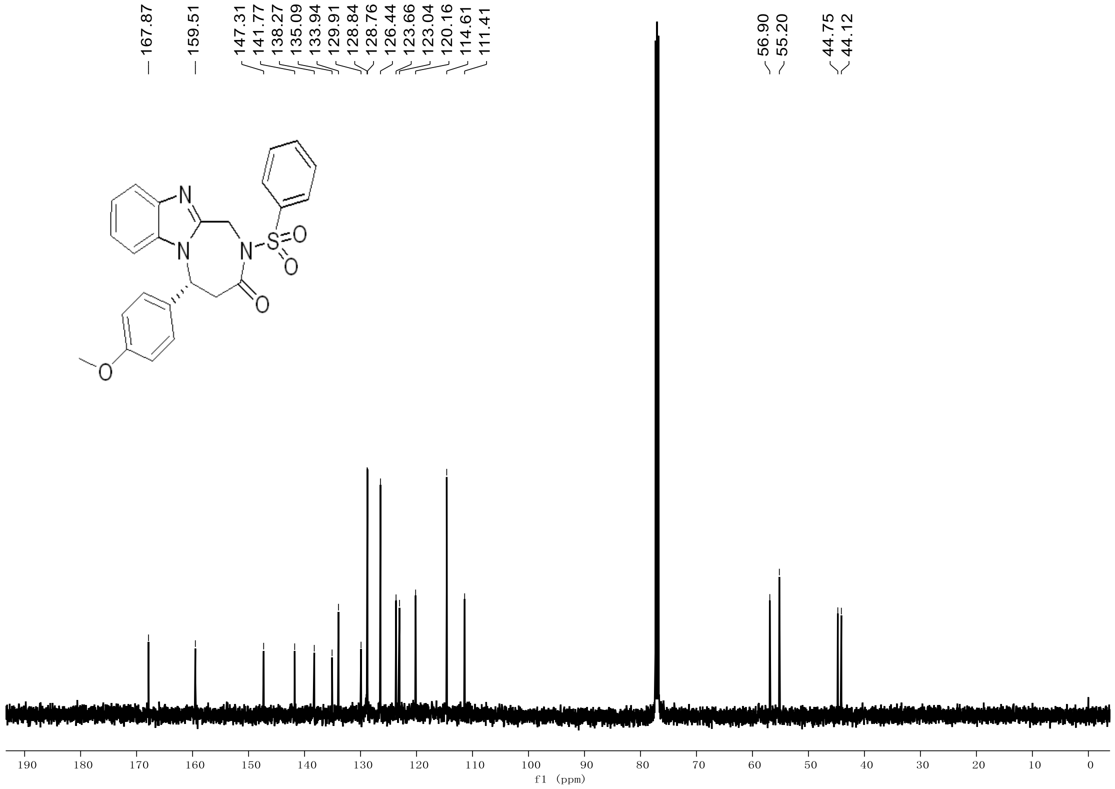

3p: ^{1}H NMR

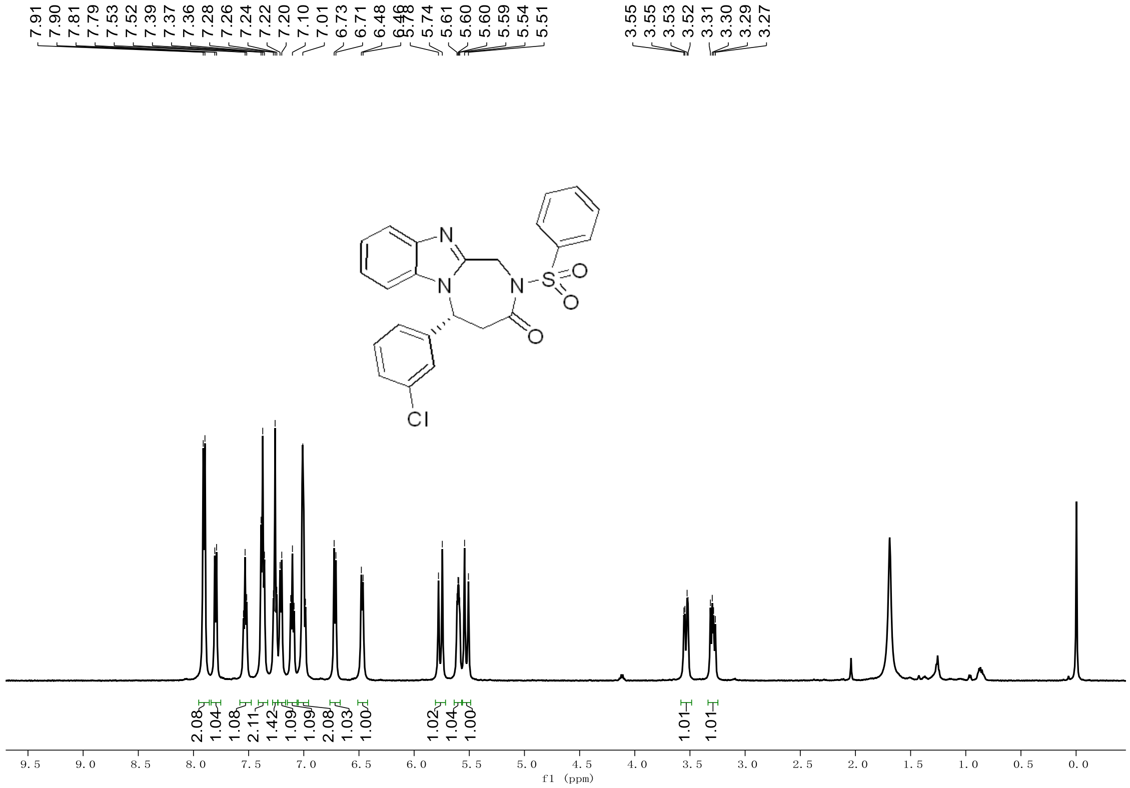

3p: ^{13}C NMR

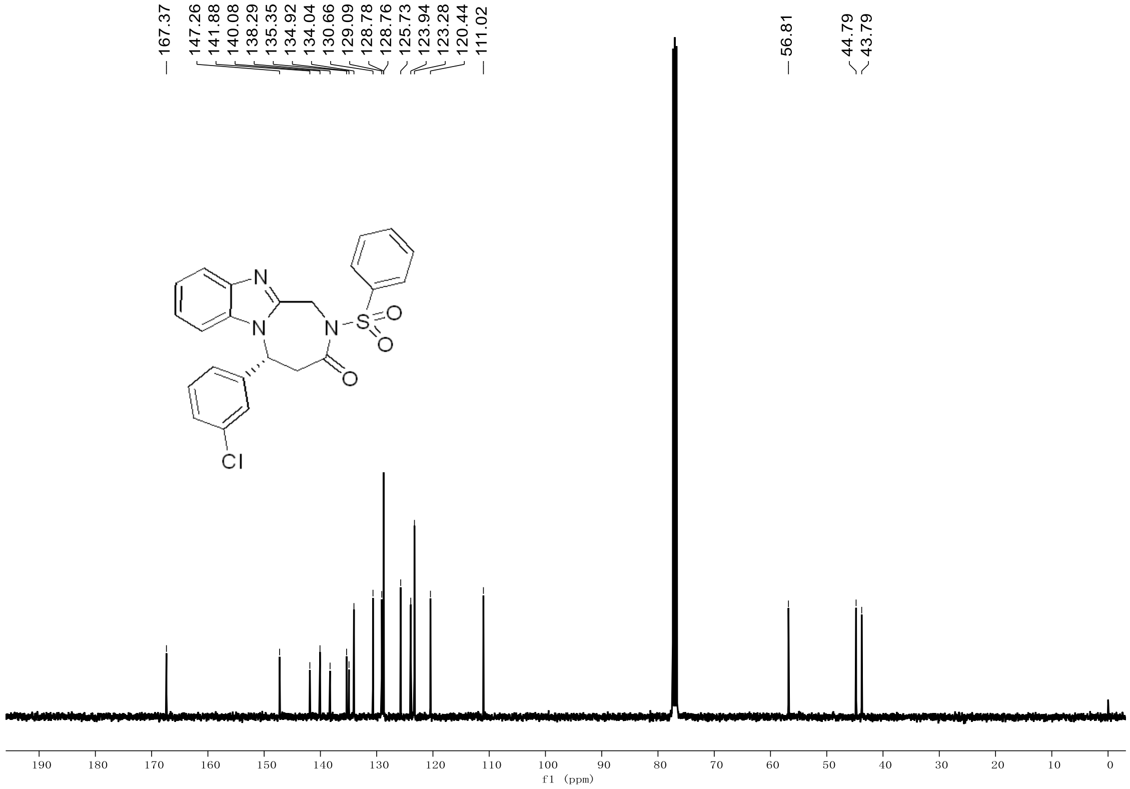

3q: ^{1}H NMR

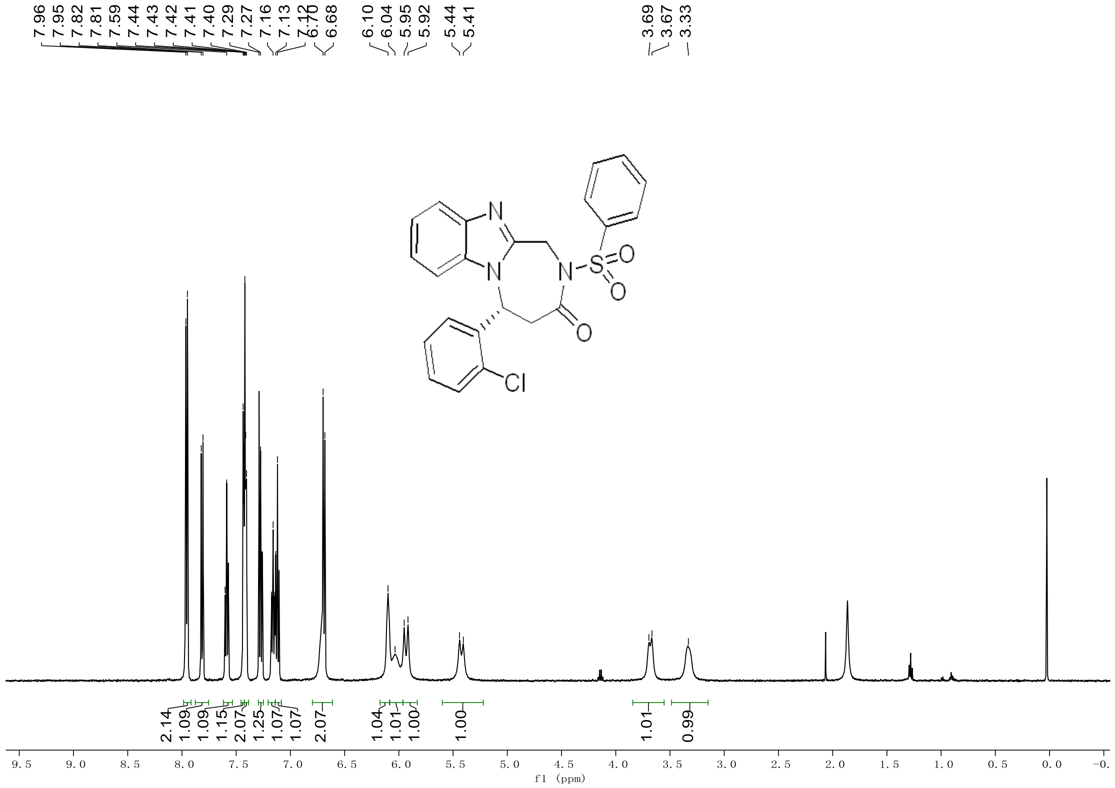

3q: ^{13}C NMR

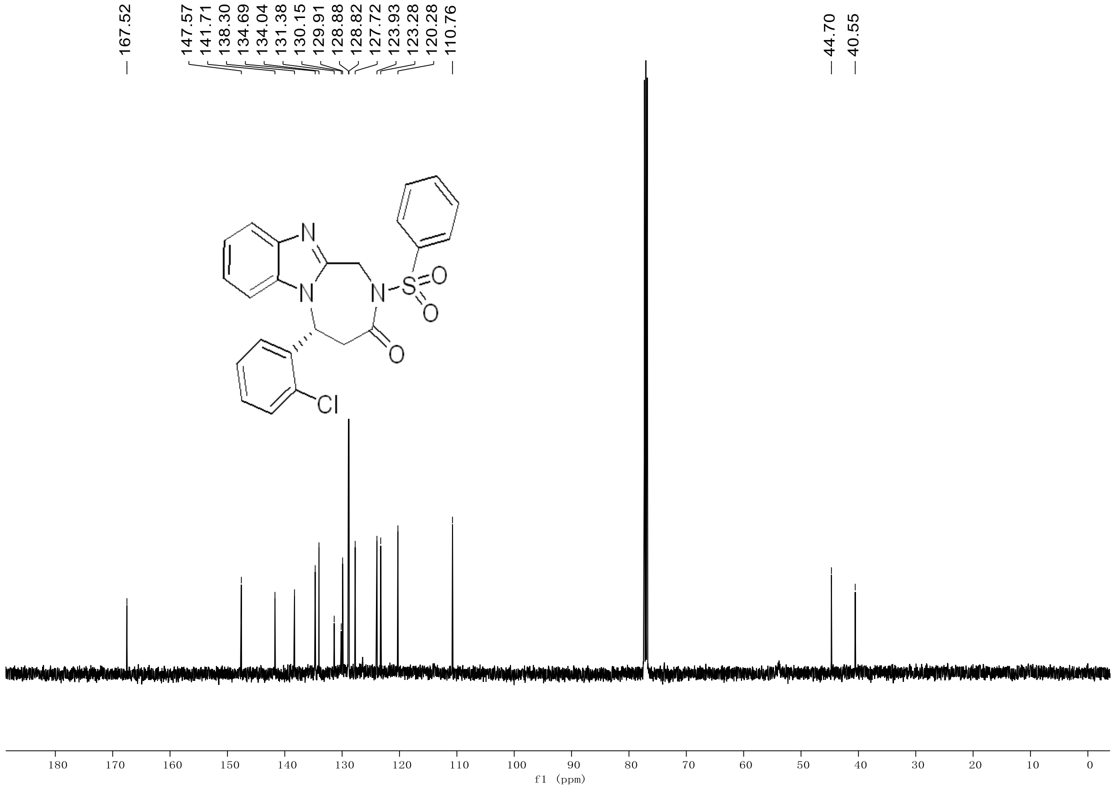

3r: ^{1}H NMR

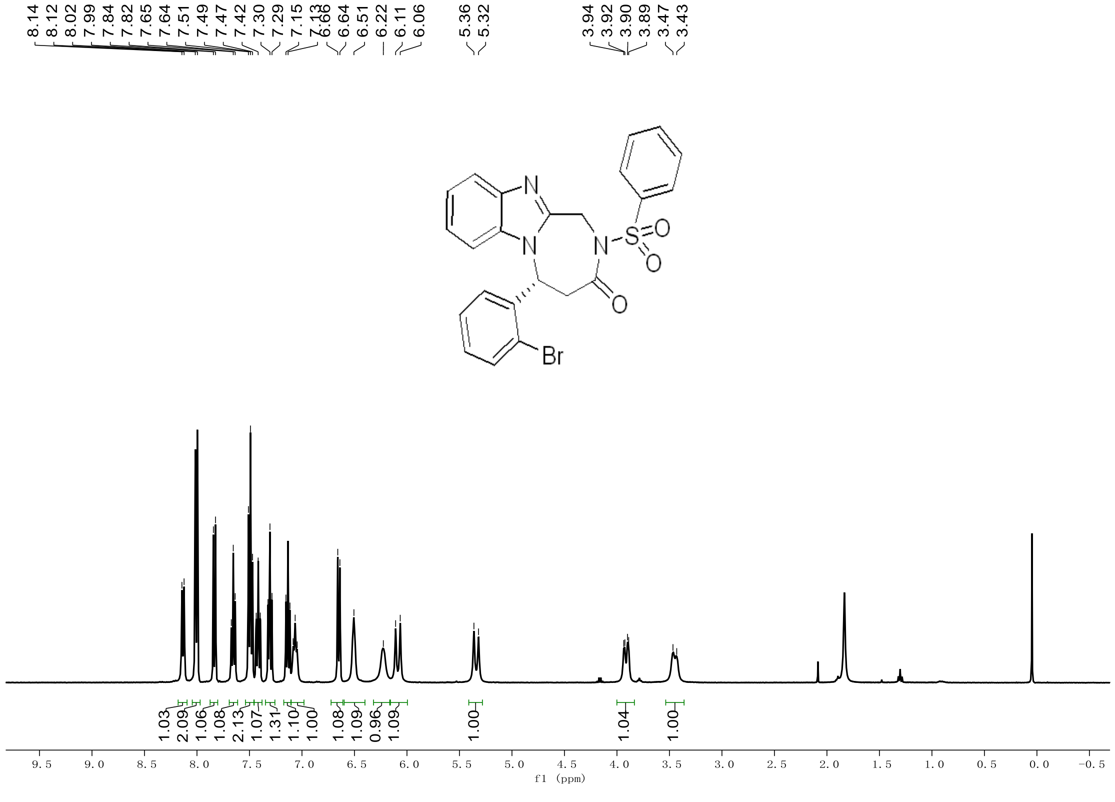

3r: ^{13}C NMR

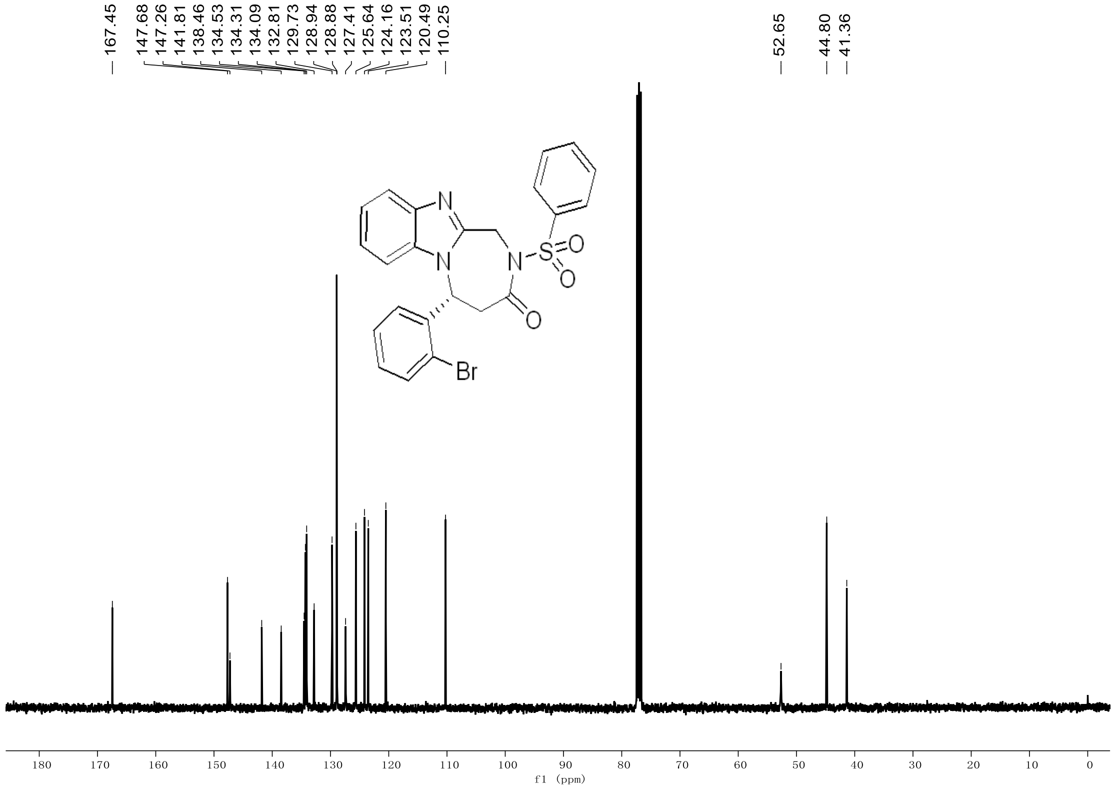

3s: ^{1}H NMR

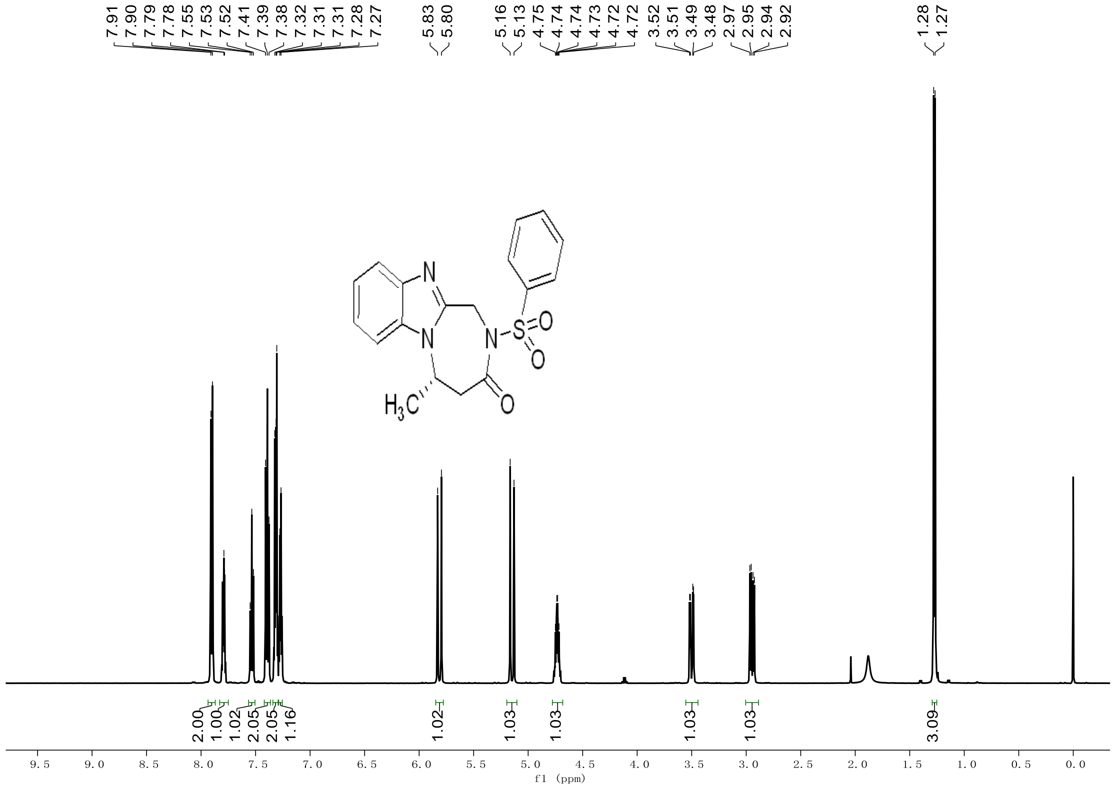

3s: ^{13}C NMR

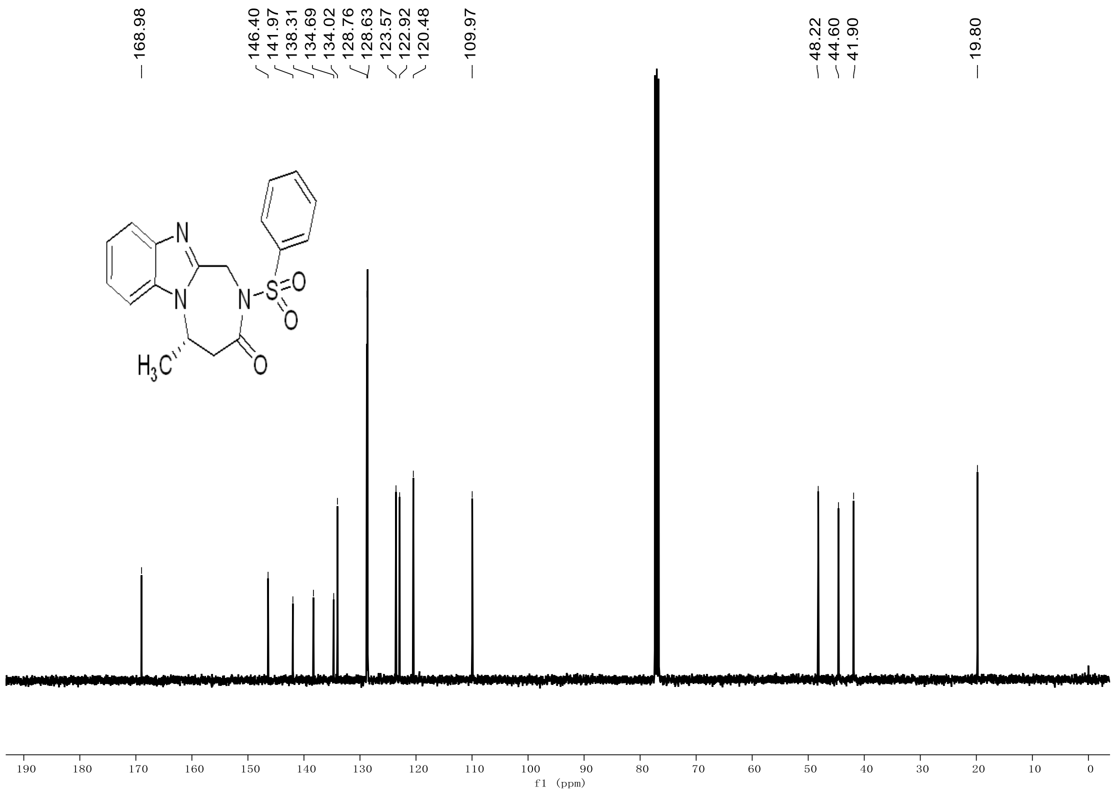

3t: ^{1}H NMR

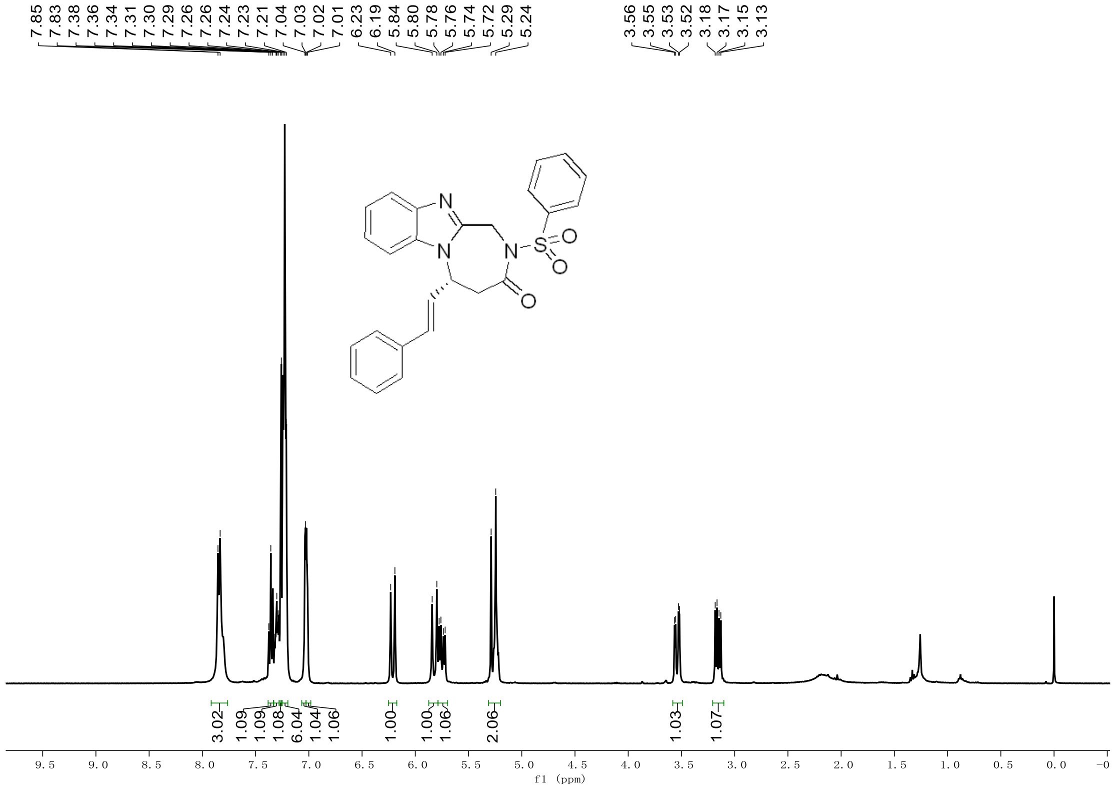

3t: ^{13}C NMR

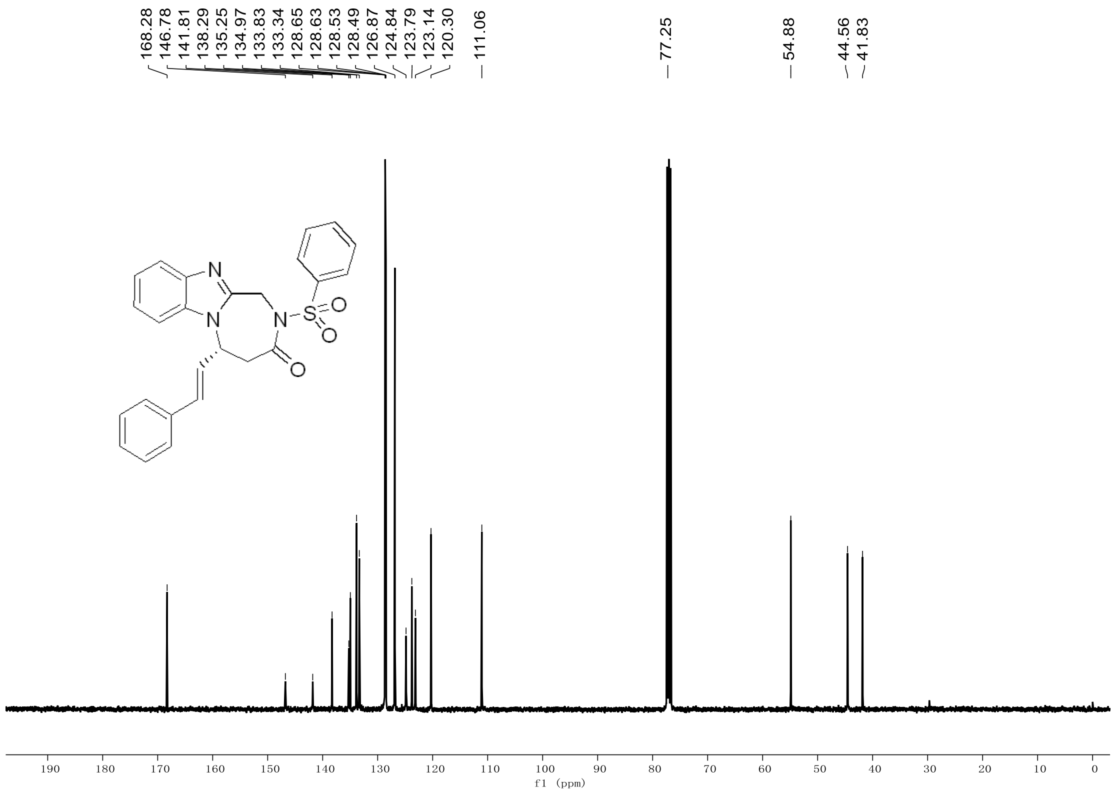

3u: ^{1}H NMR

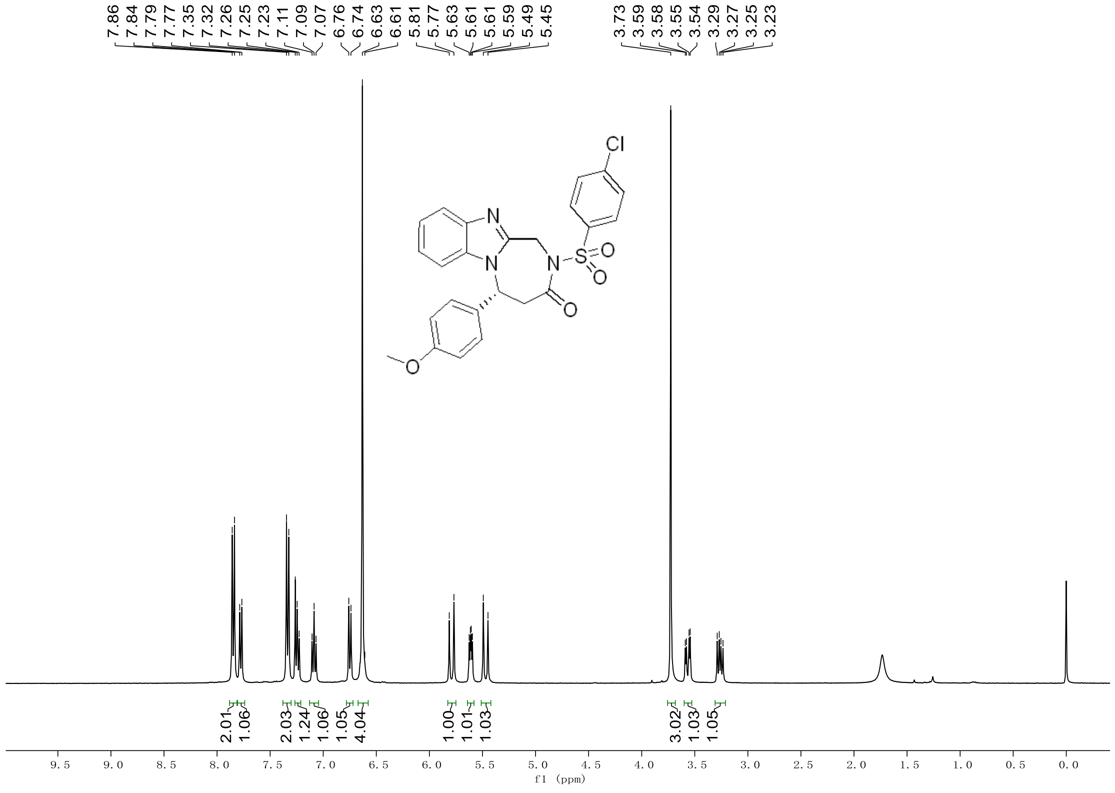

3u: ^{13}C NMR

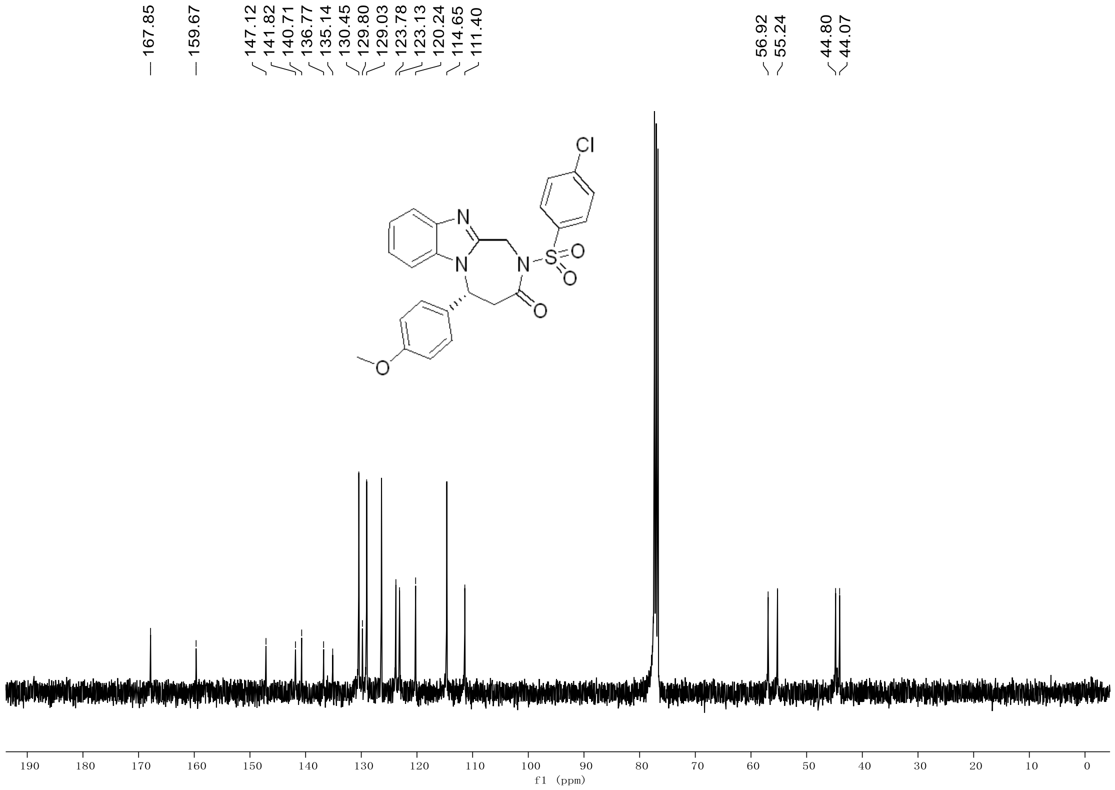

3v: ^{1}H NMR

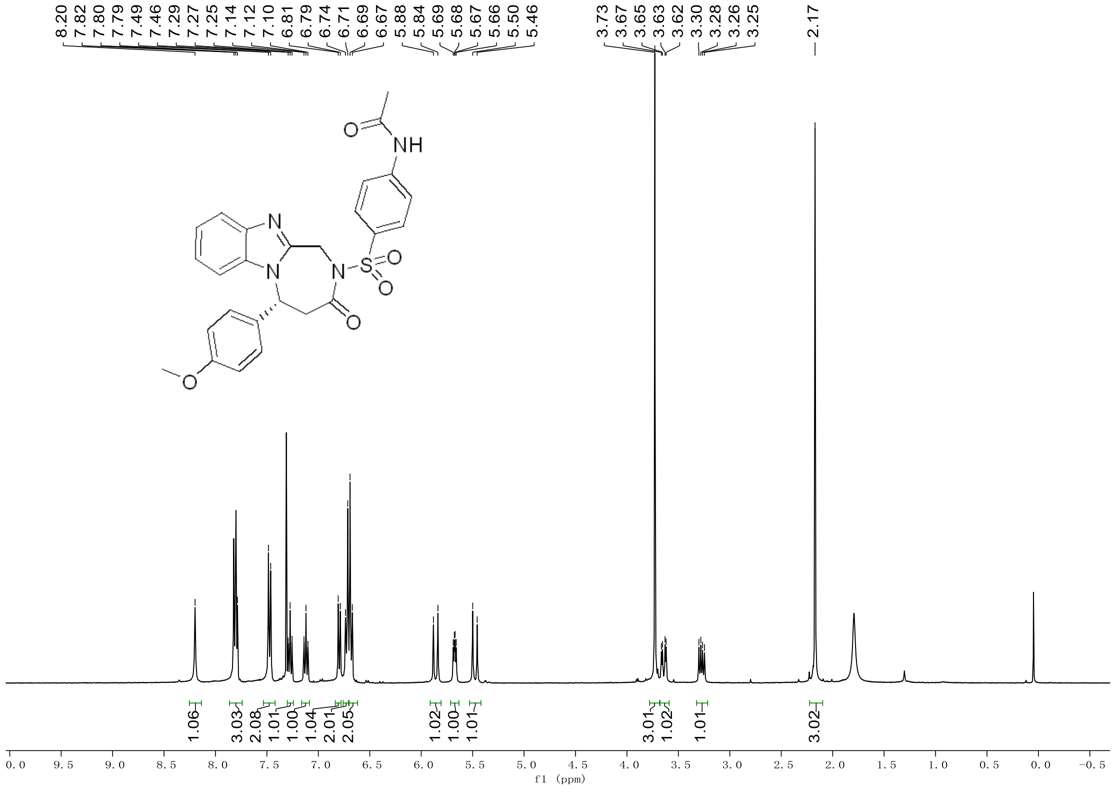

3v: ^{13}C NMR

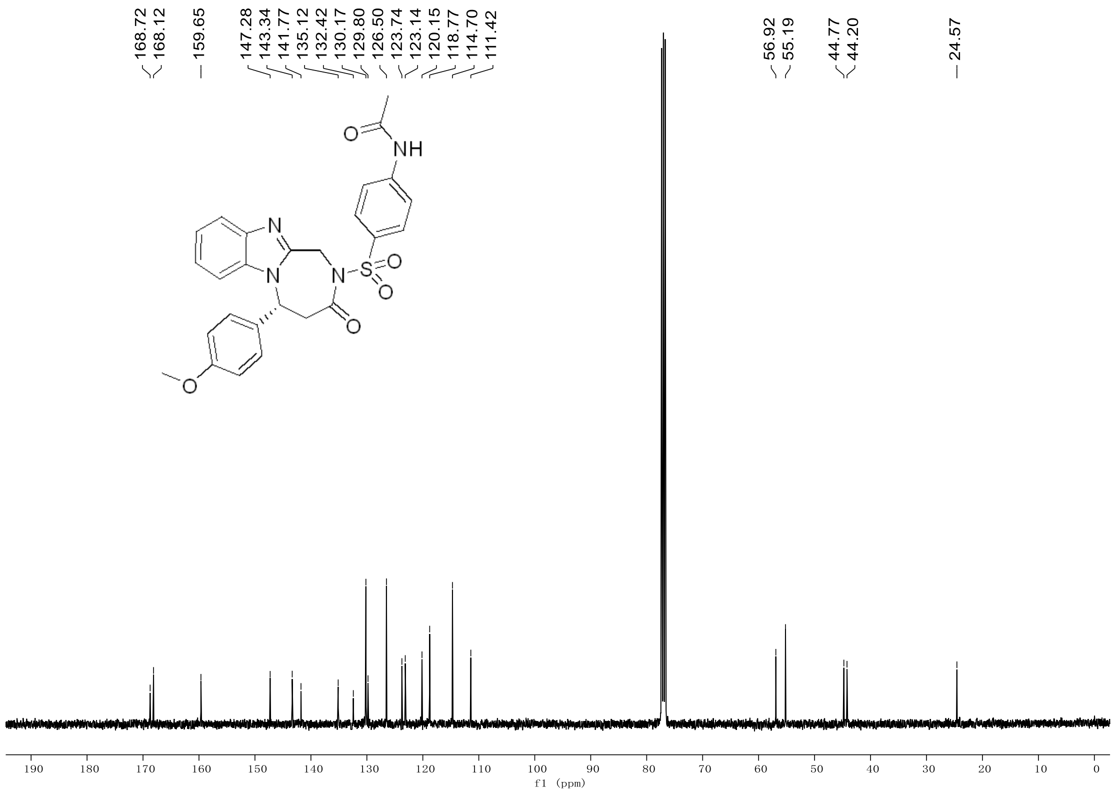

3w: ^{1}H NMR

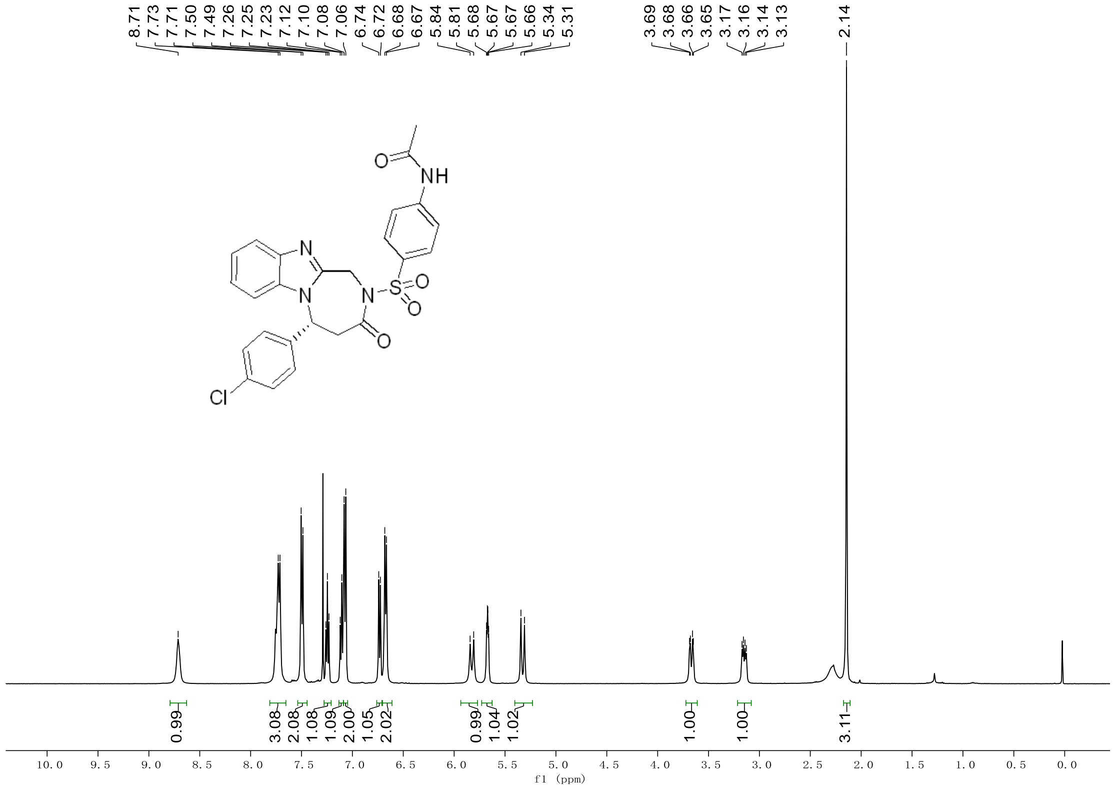

3w: ^{13}C NMR

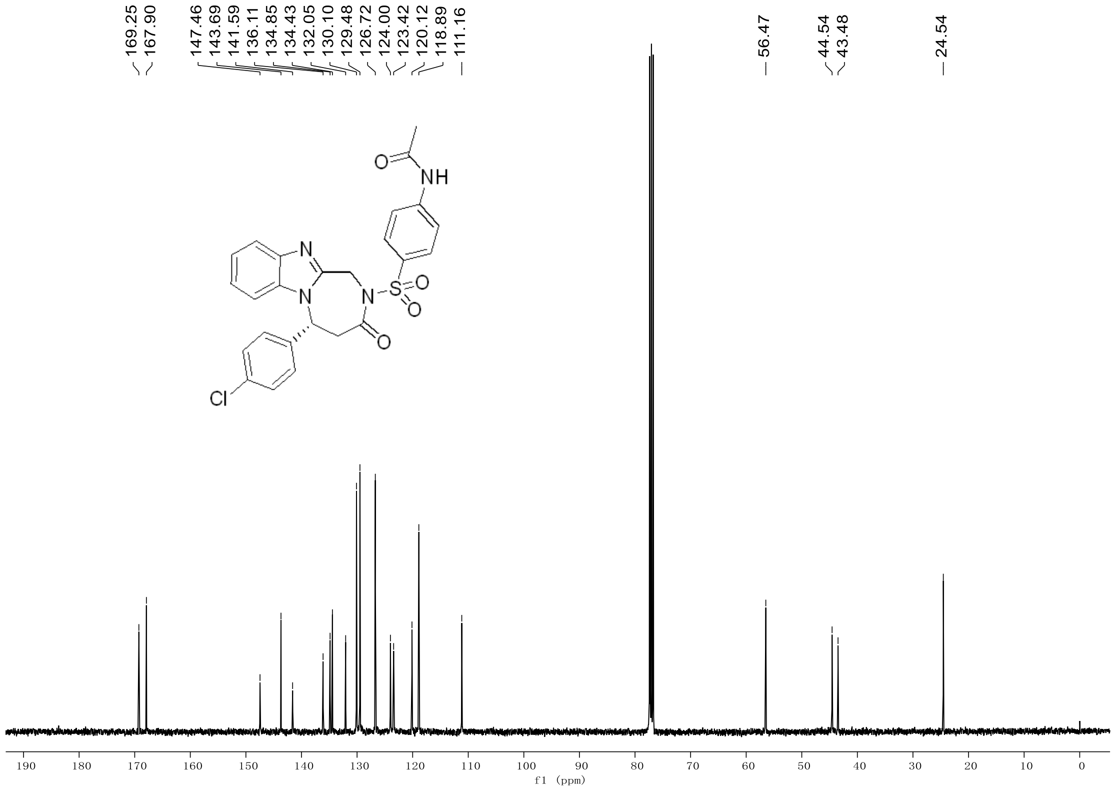

3x: ^{1}H NMR

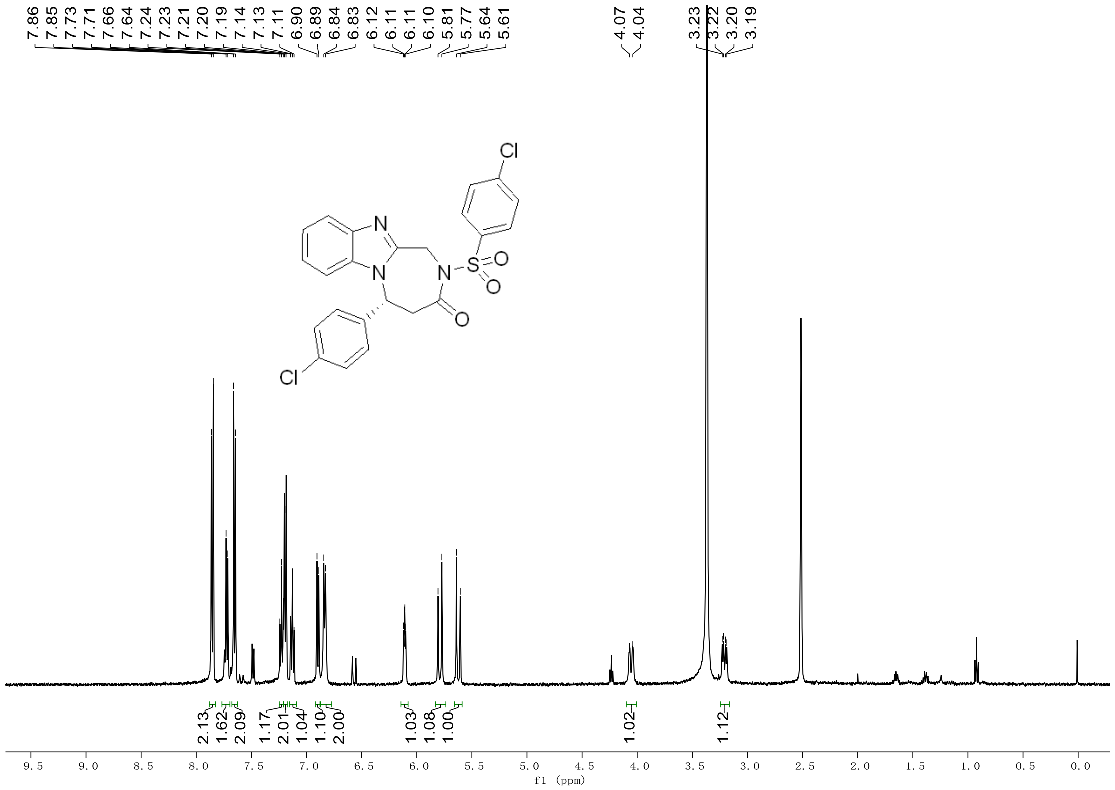

3x: ^{13}C NMR

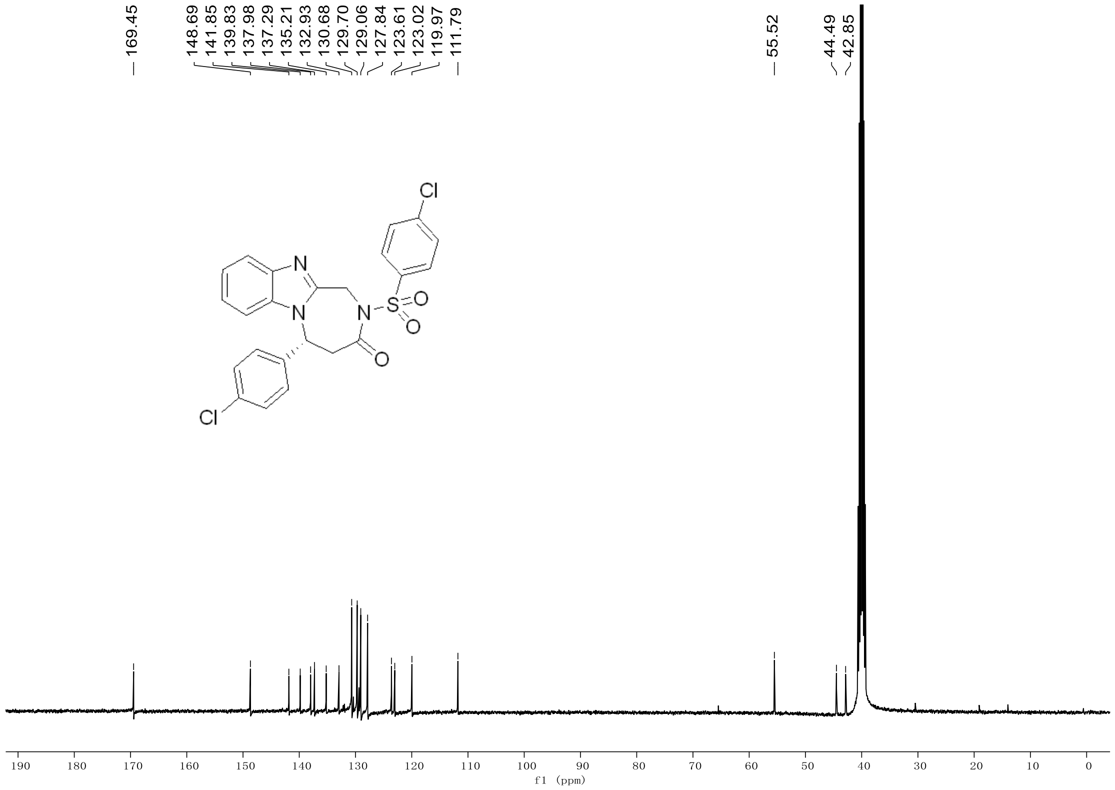

3y: ^{1}H NMR

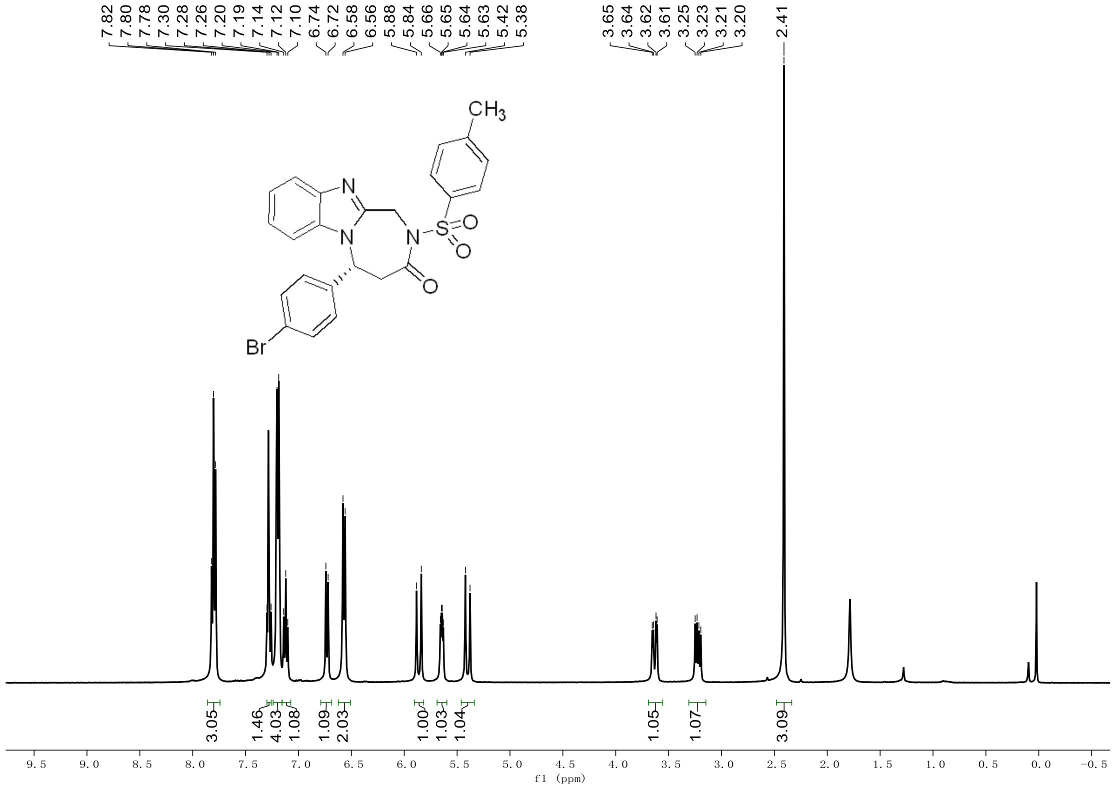

3y: ^{13}C NMR

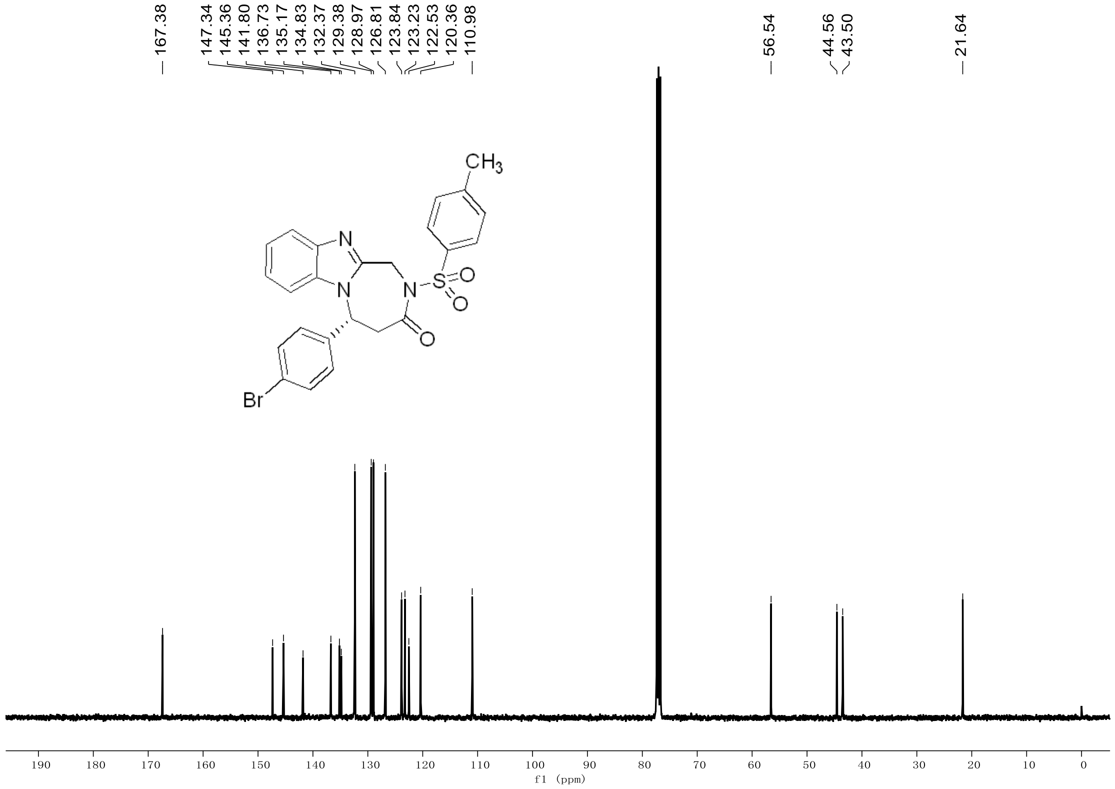

3z: ^{1}H NMR

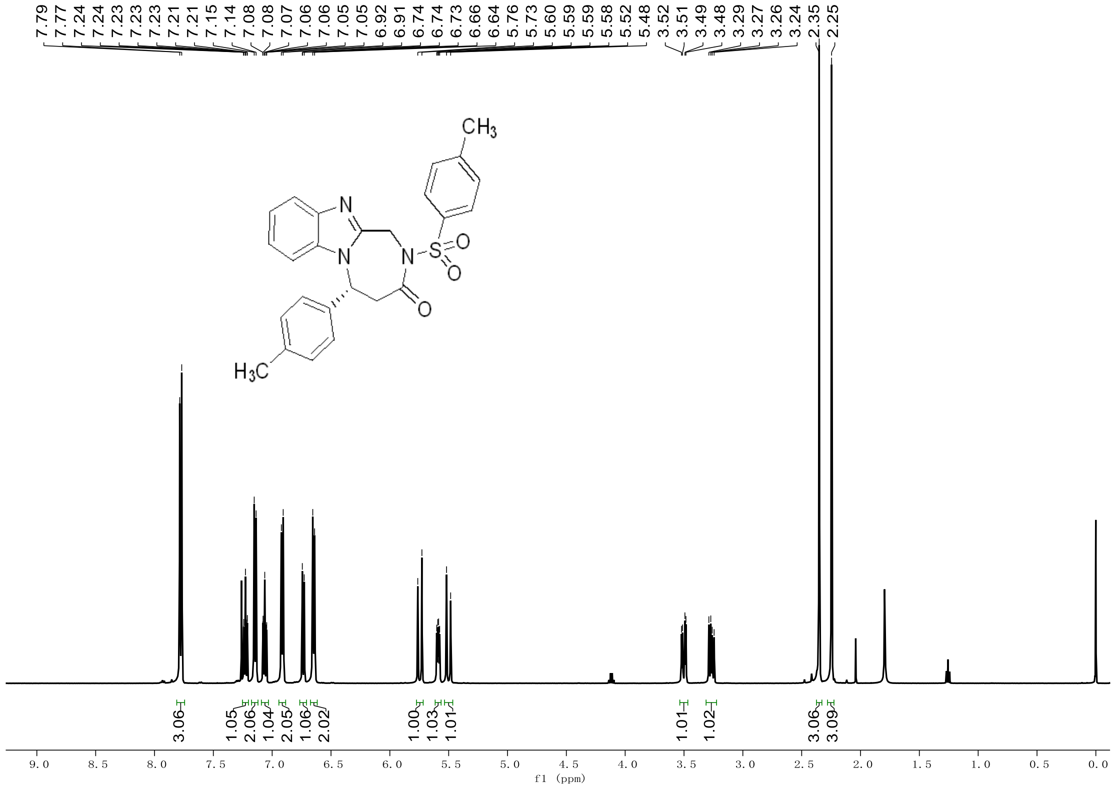

3z: ^{13}C NMR

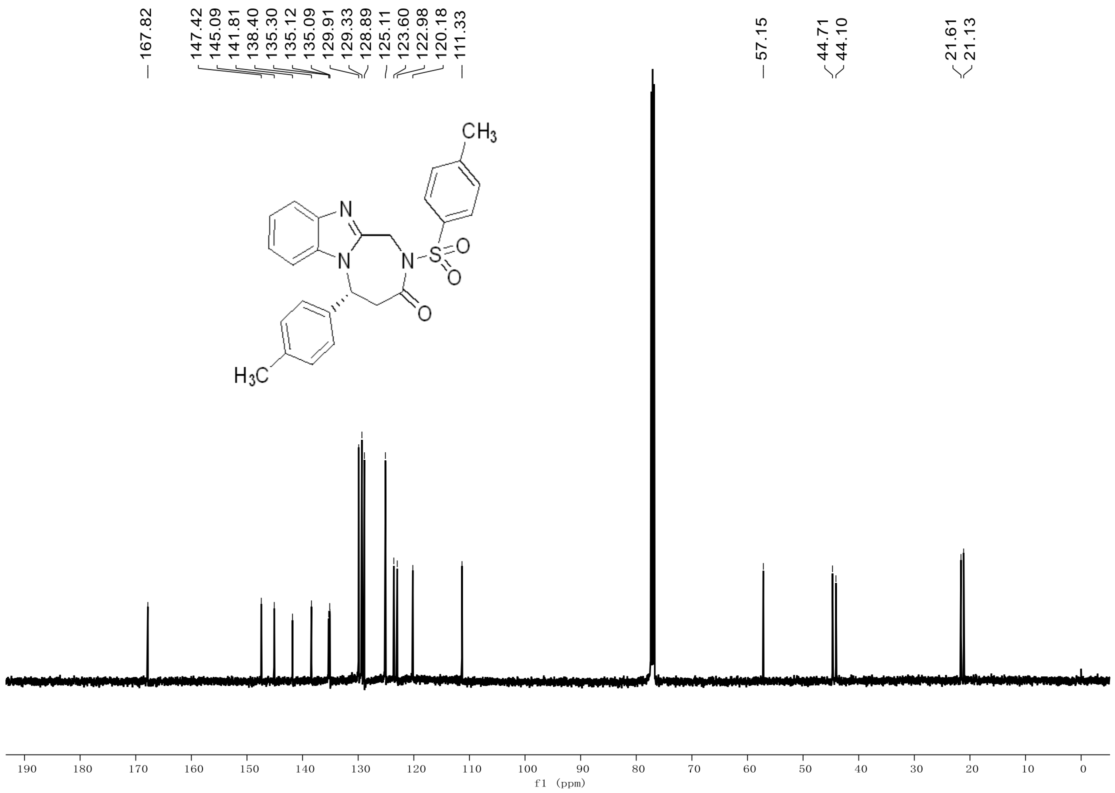

4: ^{1}H NMR

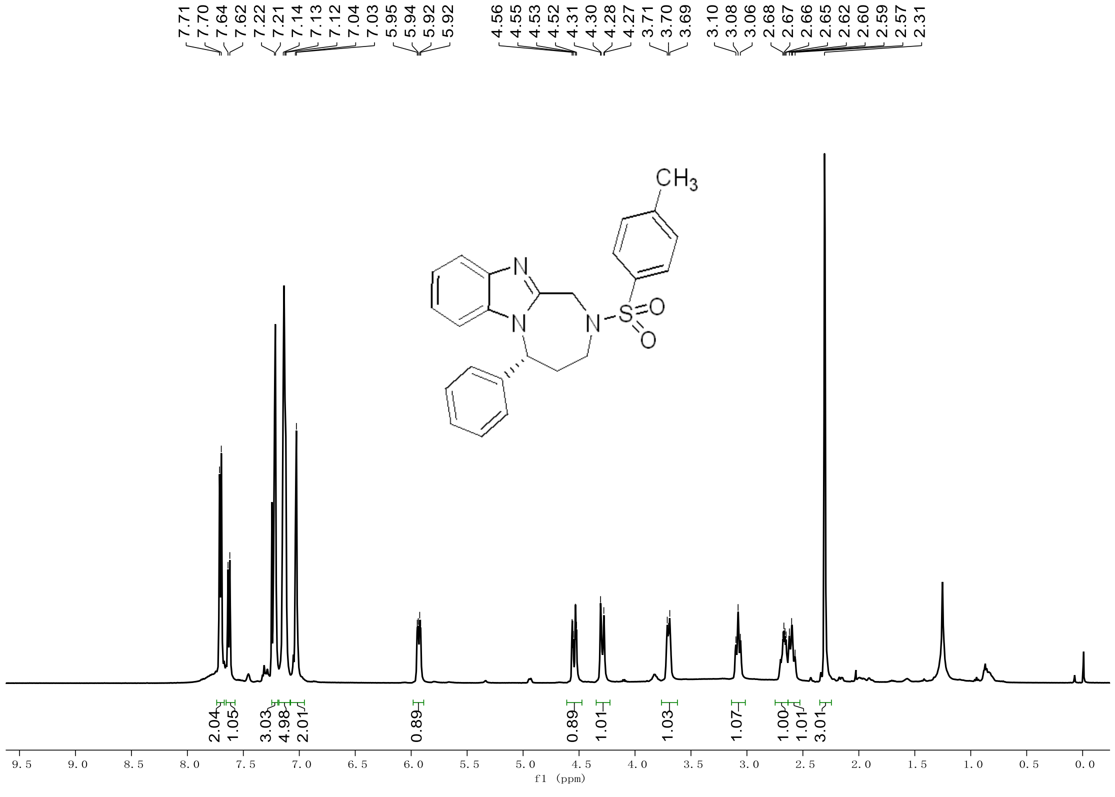

4: ^{13}C NMR

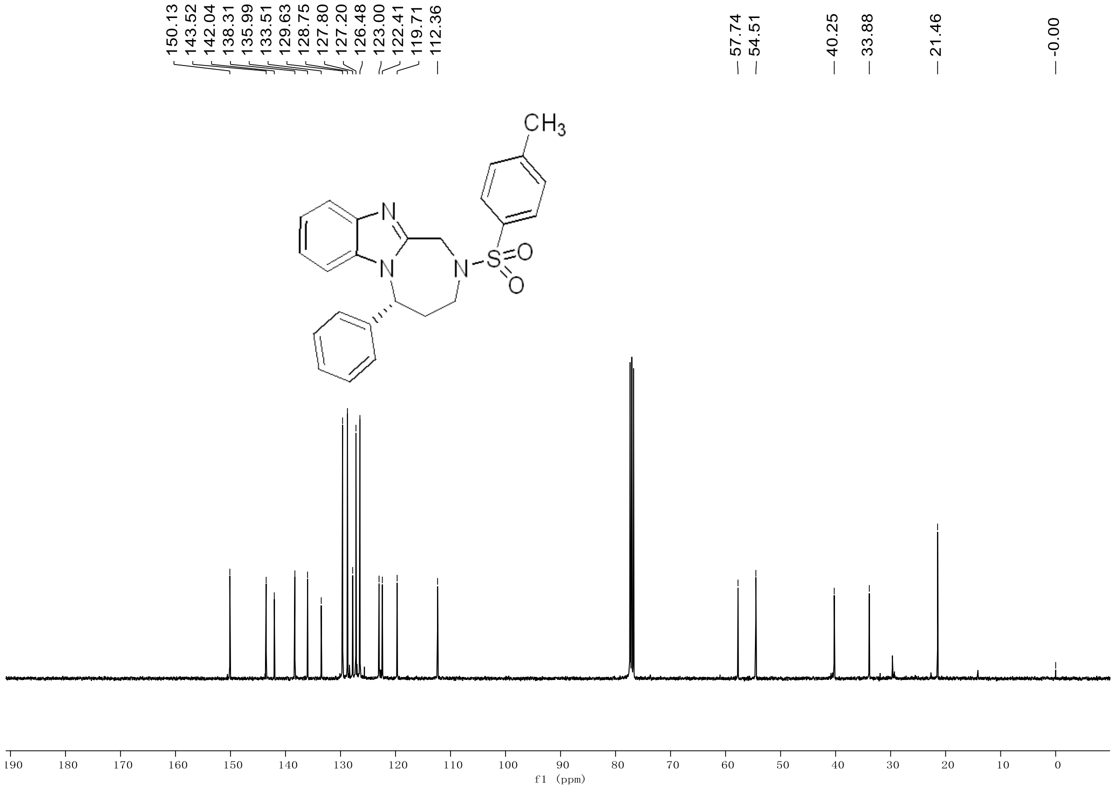

5: ^{1}H NMR

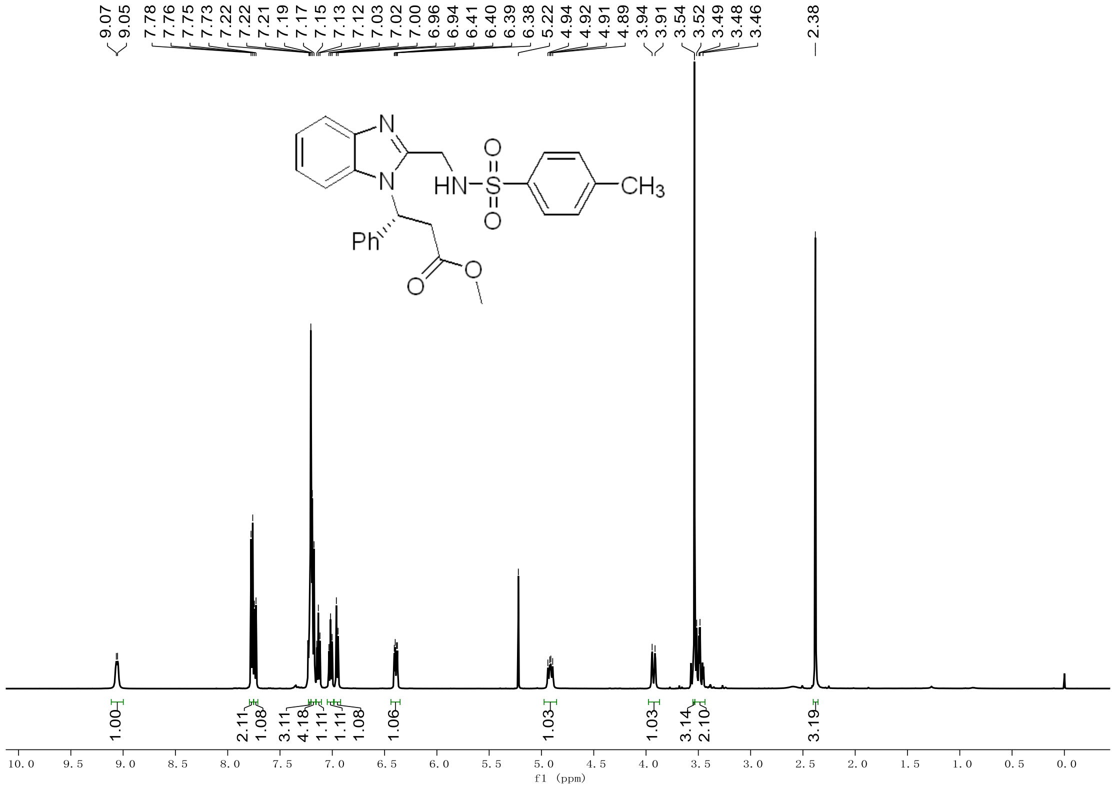

5: ^{13}C NMR

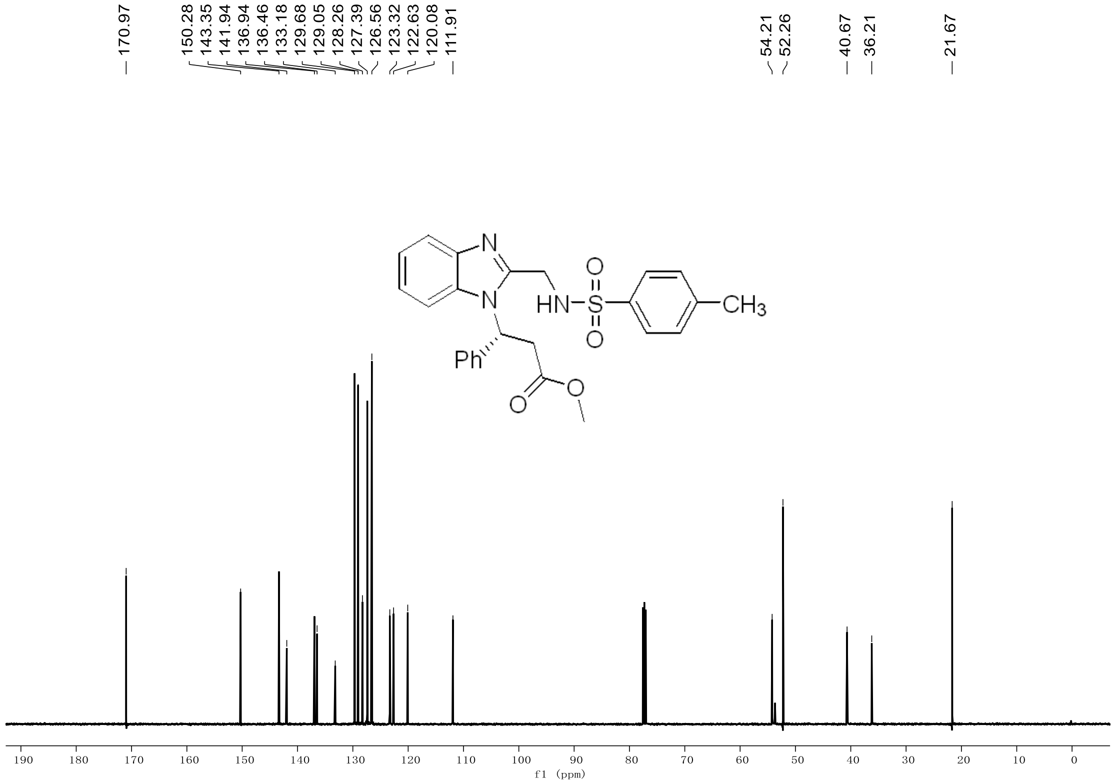
170.97
150.28
143.35
141.94
136.94
136.46
133.18
129.68
129.05
128.26
127.39
126.56
123.32
122.63
120.08
111.91
54.21
52.26
40.67
36.21
21.67
N
N
HN
S
O
O
CH3
Ph
O
O
190 180 170 160 150 140 130 120 110 100 90 80 70 60 50 40 30 20 10 0
f1 (ppm)

X. HPLC spectra of products

Racemic 3a

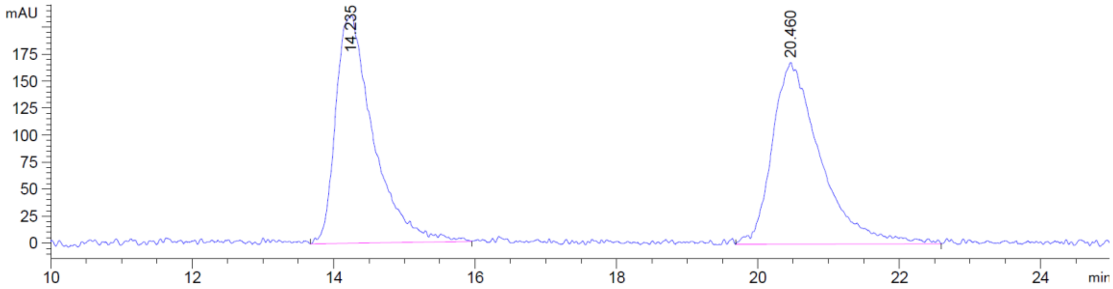

Peak#	Ret. Time	Area	Height	Area %
1	14.235	7814.60107	210.1821	49.6083
2	20.460	7938.01221	168.0347	50.3917
Total		1.58E+04	378.2168	100

(R)-3a

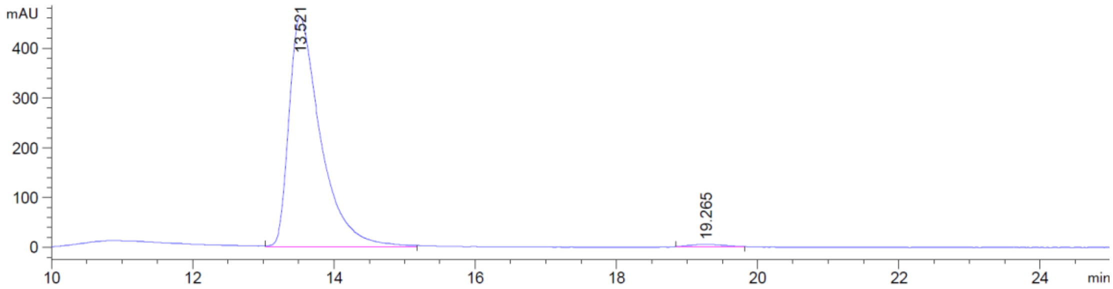

Peak#	Ret. Time	Area	Height	Area %
1	13.521	1.44E+04	461.62936	99.2410
2	19.265	109.93604	4.05708	0.7590
Total		1.45E+04	465.68644	100

(S)-3a

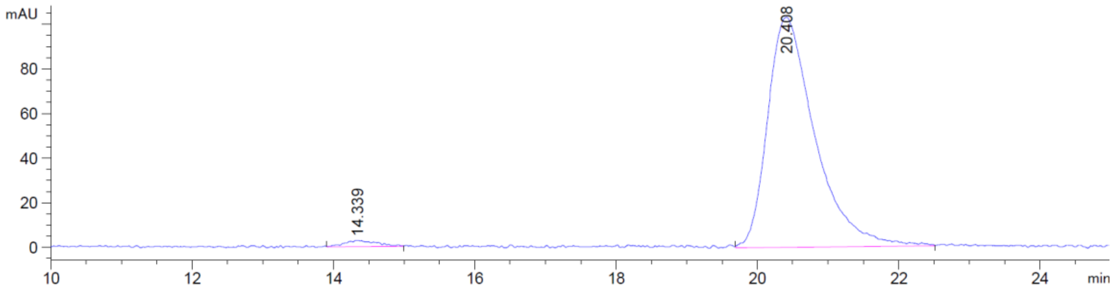

Peak#	Ret. Time	Area	Height	Area %
1	14.339	89.43652	2.77137	1.8250
2	20.408	4811.23145	103.21333	98.1750
Total		4900.66796	105.9847	100

Racemic 3b

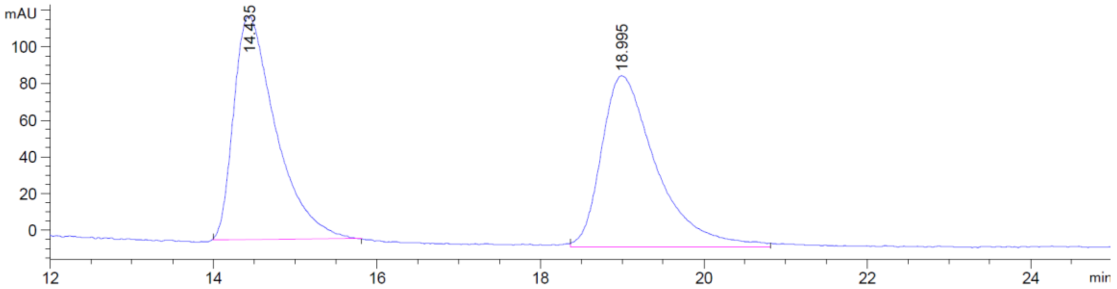

Peak#	Ret. Time	Area	Height	Area %
1	14.435	4367.68604	121.88024	50.0104
2	18.995	4365.86572	93.32619	49.9896
Total		8733.55176	215.20643	100

(R)-3b

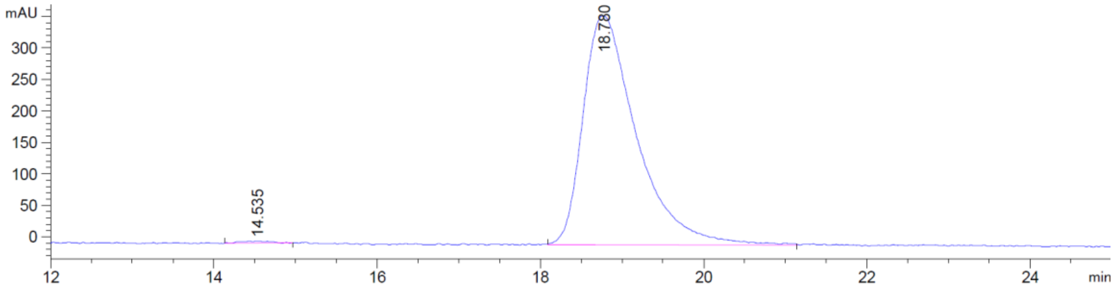

Peak#	Ret. Time	Area	Height	Area %
1	14.535	63.92875	2.84858	0.3837
2	18.78	1.66E+04	363.36917	99.6163
Total		1.67E+04	366.21775	100

(S)-3b

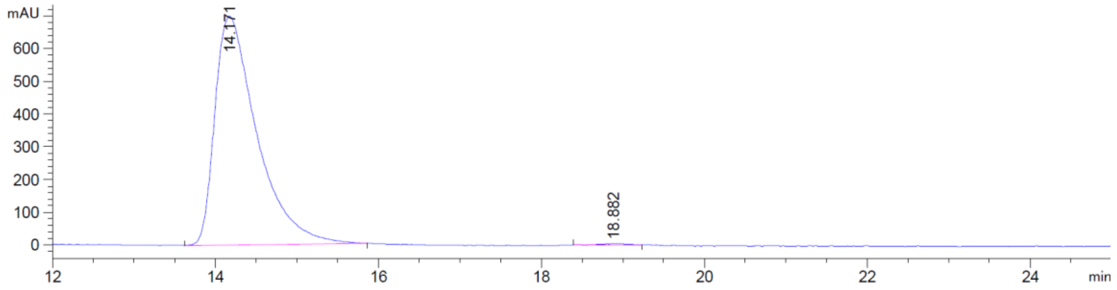

Peak#	Ret. Time	Area	Height	Area %
1	14.171	2.50E+04	699.14673	99.5758
2	18.882	106.59032	4.22179	0.4242
Total		2.51E+04	703.36852	100

Racemic 3c

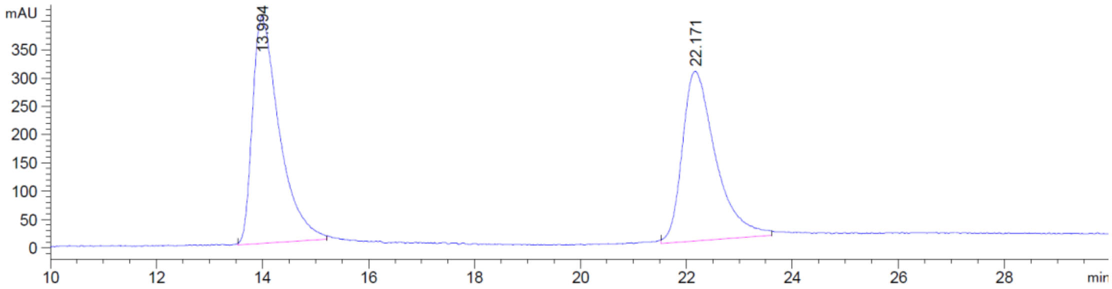

Peak#	Ret. Time	Area	Height	Area %
1	13.994	1.41E+04	400.92038	51.9006
2	22.171	1.31E+04	299.53528	48.0994
Total		2.72E+04	700.45566	100

(R)-3c

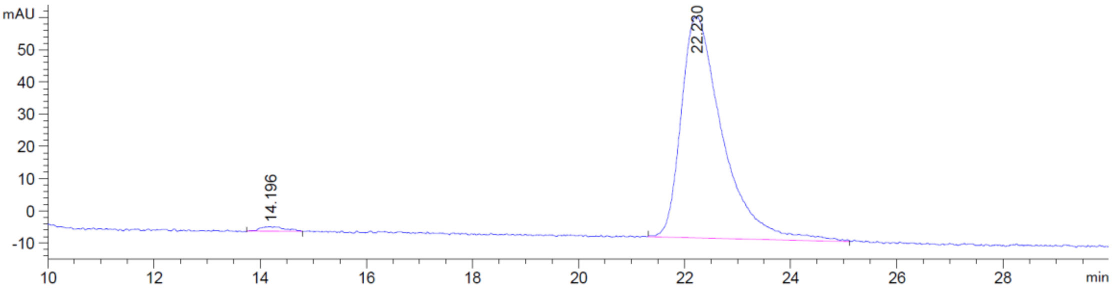

Peak#	Ret. Time	Area	Height	Area %
1	14.196	48.81293	1.49173	1.2956
2	22.23	3718.73657	68.74124	98.7044
Total		3767.5495	70.23297	100

(S)-3c

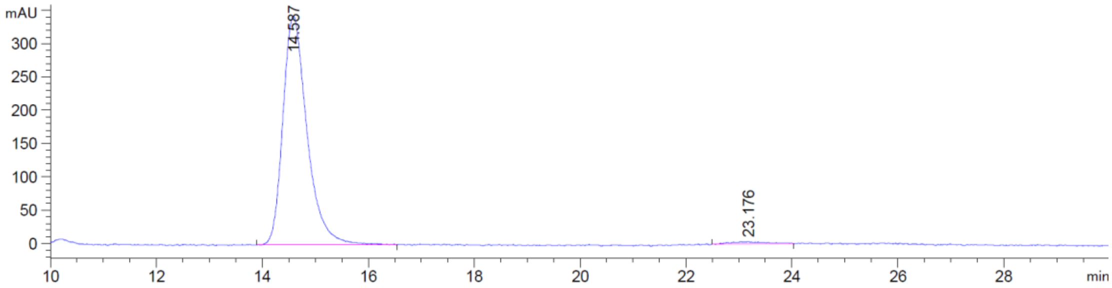

Peak#	Ret. Time	Area	Height	Area %
1	14.587	1.09E+04	343.74408	98.7962
2	23.176	133.28378	3.34832	1.2038
Total		1.11E+04	347.0924	100

Racemic 3d

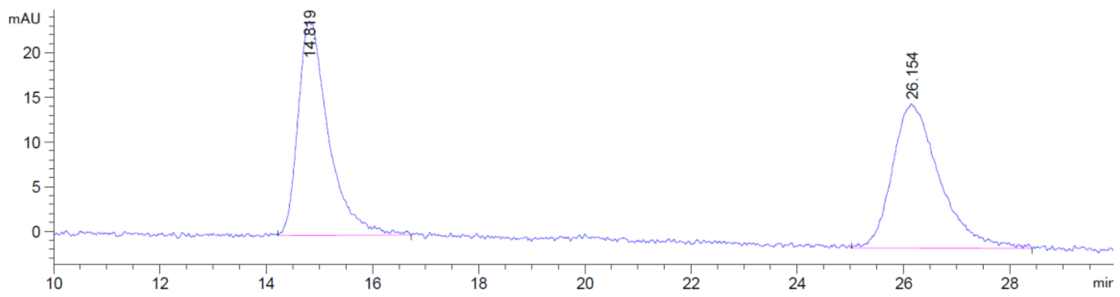

Peak#	Ret. Time	Area	Height	Area %
1	14.819	954.80884	23.95081	48.9310
2	26.154	996.52808	16.18719	51.0690
Total		1951.33691	40.13801	100

(R)-3d

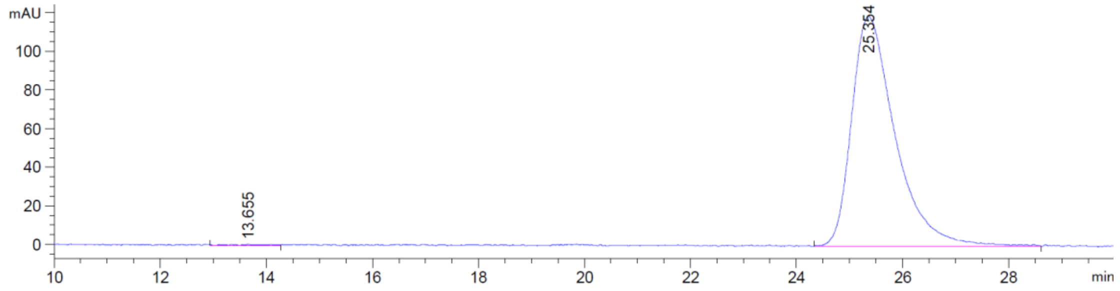

Peak#	Ret. Time	Area	Height	Area %
1	13.655	25.5097	6.85E-01	0.3757
2	25.354	6764.90039	119.15955	99.6243
Total		6790.41009	119.84428	100

(S)-3d

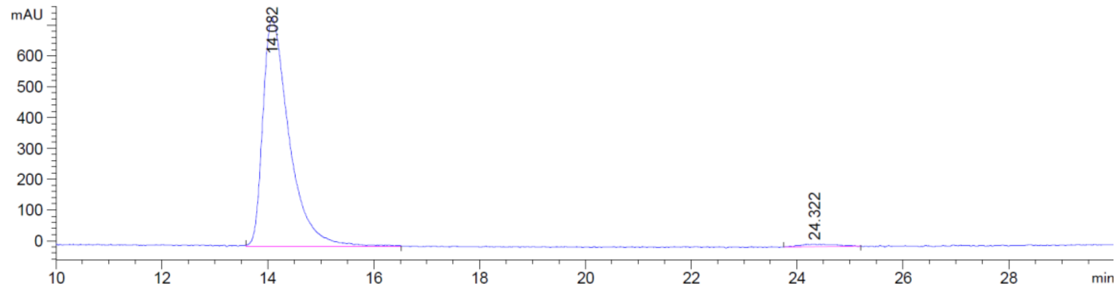

Peak#	Ret. Time	Area	Height	Area %
1	14.082	2.55E+04	741.49084	98.4028
2	24.322	413.23407	9.19298	1.5972
Total		2.59E+04	750.68383	100

Racemic 3e

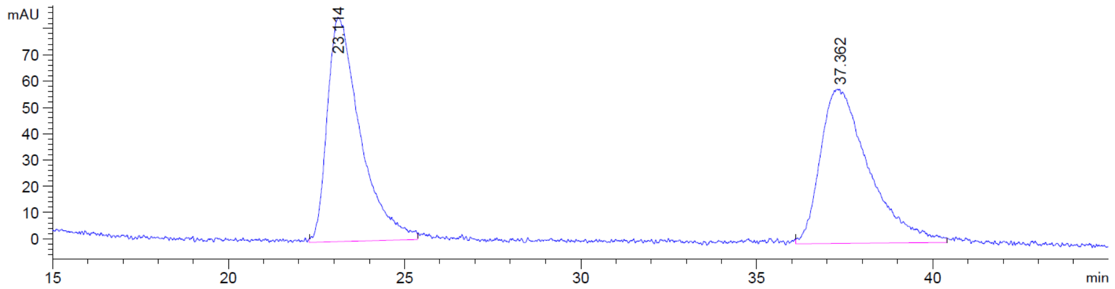

Peak#	Ret. Time	Area	Height	Area %
1	23.114	5555.03857	85.38091	49.6787
2	37.362	5626.9043	58.69958	50.3213
Total		1.12E+04	144.08049	100

(R)-3e

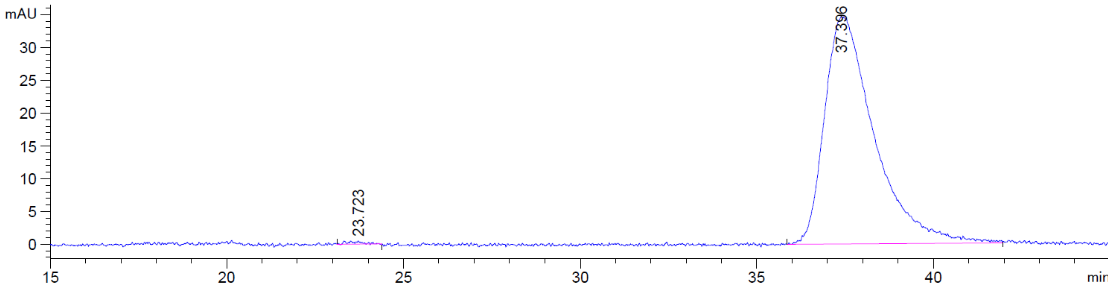

Peak#	Ret. Time	Area	Height	Area %
1	23.723	14.30759	5.00E-01	0.4197
2	37.396	3394.87549	34.65944	99.5803
Total		3409.18308	35.15946	100

(S)-3e

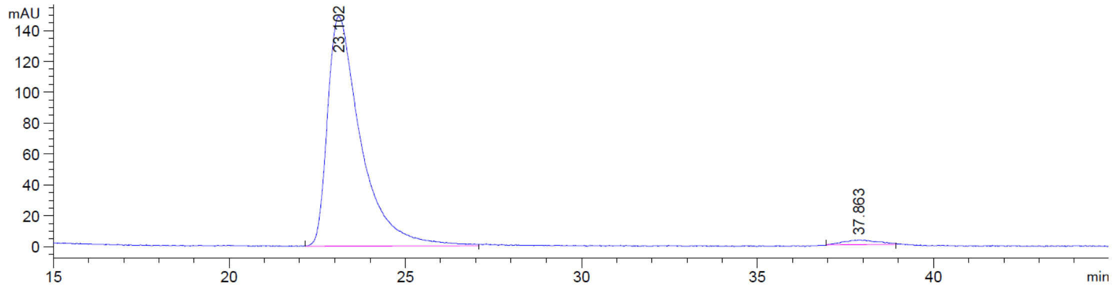

Peak#	Ret. Time	Area	Height	Area %
1	23.102	9794.20215	148.87308	97.9143
2	37.863	208.63208	3.14206	2.0857
Total		1.00E+04	152.01514	100

Racemic 3f

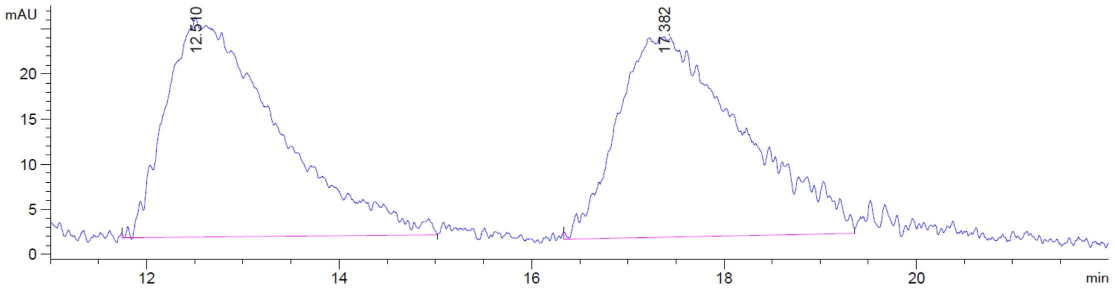

Peak#	Ret. Time	Area	Height	Area %
1	12.510	1957.40686	24.37128	49.3701
2	17.382	2007.35095	22.26219	50.6299
Total		3964.75781	46.63347	100

(R)-3f

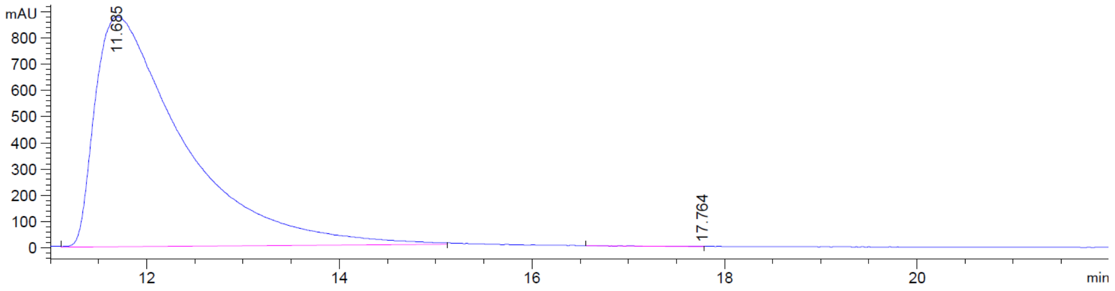

Peak#	Ret. Time	Area	Height	Area %
1	11.685	5.64E+04	877.06854	99.8829
2	17.764	66.16866	1.59644	0.1171
Total		5.65E+04	878.66498	100

(S)-3f

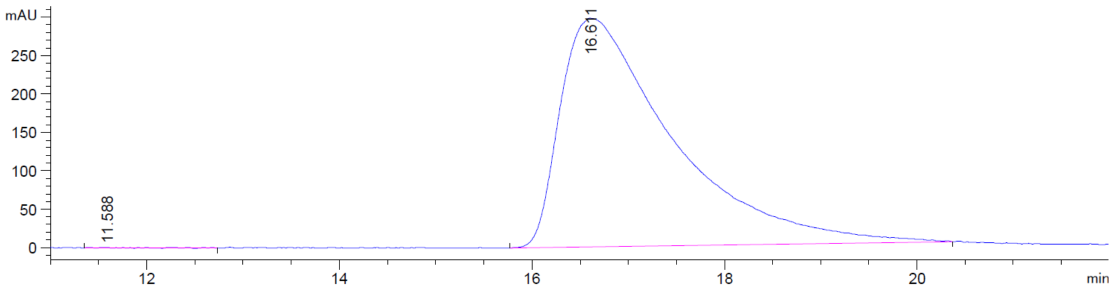

Peak#	Ret. Time	Area	Height	Area %
1	11.588	29.31755	0.900179	0.121
2	16.611	2.42E+04	297.99976	99.879
Total		2.42E+04	298.89993	100

Racemic 3g

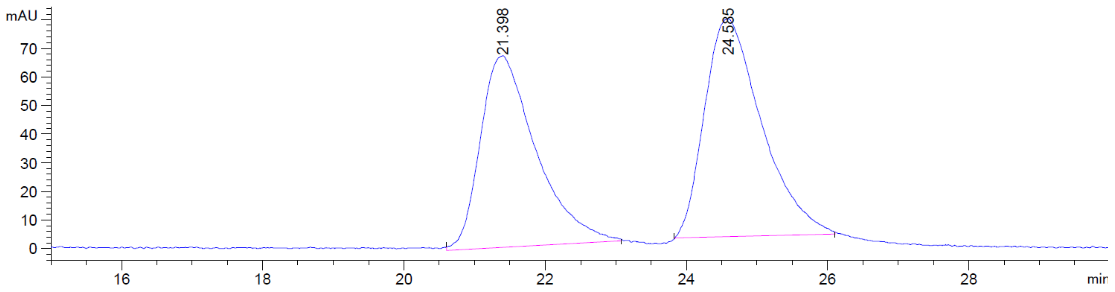

Peak#	Ret. Time	Area	Height	Area %
1	21.398	3678.48486	67.01451	46.1965
2	24.585	4284.20801	76.21009	53.8035
Total		7962.69287	143.2246	100

(R)-3g

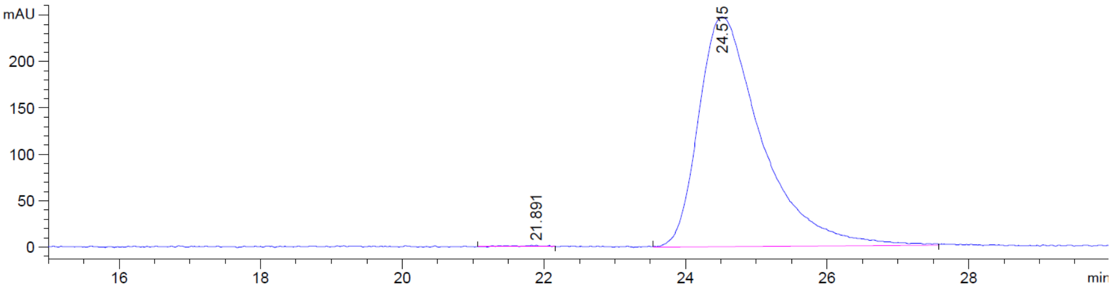

Peak#	Ret. Time	Area	Height	Area %
1	21.891	41.89524	1.44861	0.2734
2	24.515	1.53E+04	247.46713	99.7266
Total		1.53E+04	248.91574	100

(S)-3g

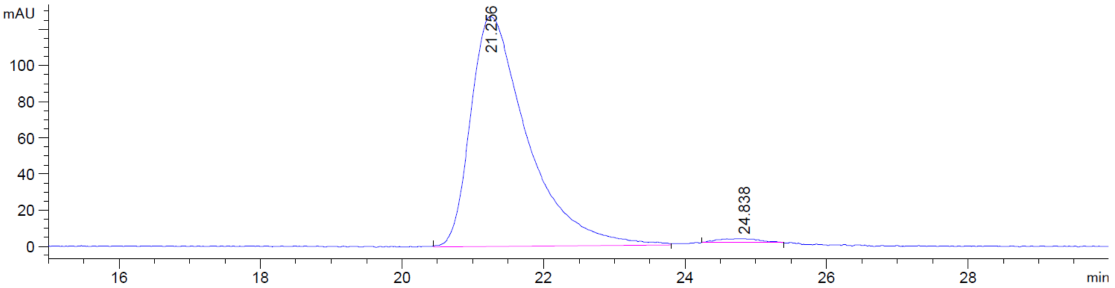

Peak#	Ret. Time	Area	Height	Area %
1	21.256	7099.78809	127.23132	98.7189
2	24.838	92.13232	2.30065	1.2811
Total		7191.92041	129.53197	100

Racemic 3h

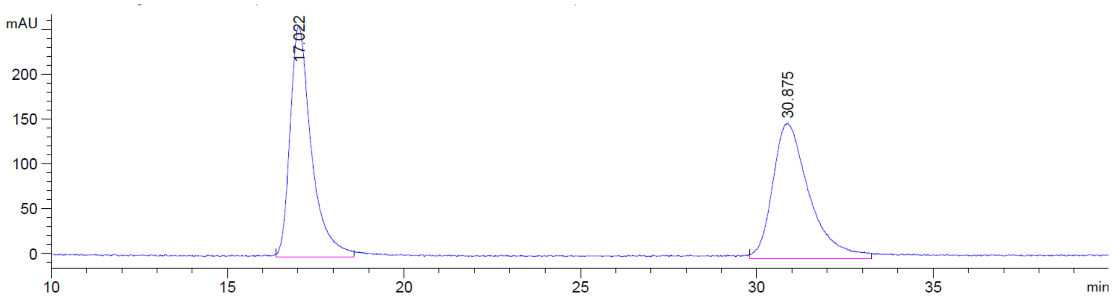

Peak#	Ret. Time	Area	Height	Area %
1	17.022	1.08E+04	257.9747	49.9041
2	30.875	1.09E+04	151.23686	50.0959
Total		2.17E+04	409.21156	100

(R)-3h

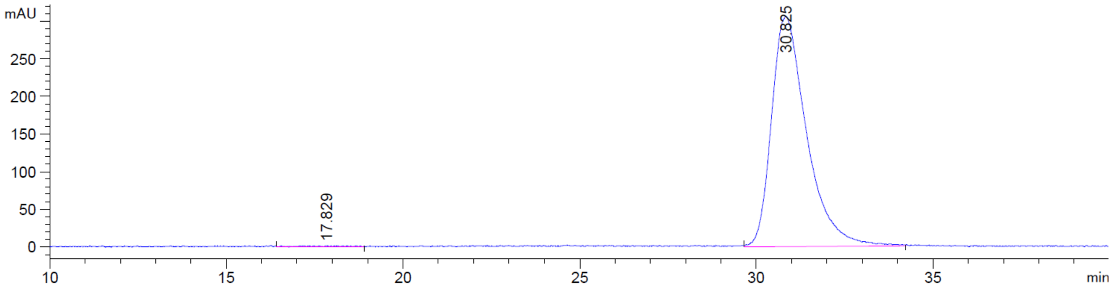

Peak#	Ret. Time	Area	Height	Area %
1	17.829	69.20311	1.31143	0.3255
2	30.825	2.12E+04	305.80008	99.6745
Total		2.13E+04	307.11151	100

(S)-3h

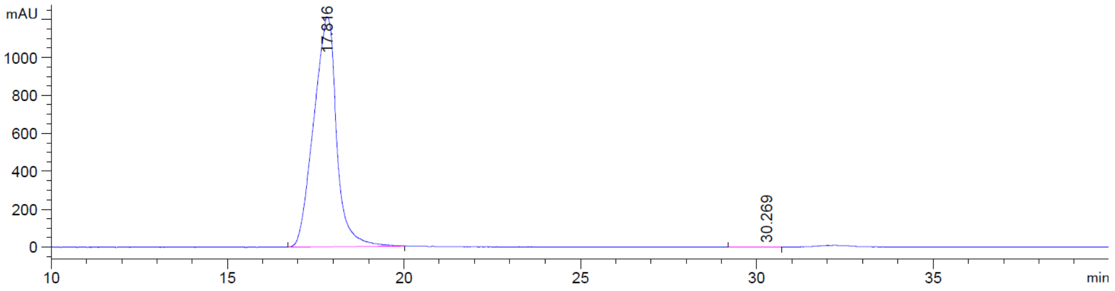

Peak#	Ret. Time	Area	Height	Area %
1	17.816	5.45E+04	1213.47607	99.8524
2	30.269	80.60387	1.64067	0.1476
Total		5.46E+04	1215.11674	100

Racemic 3i

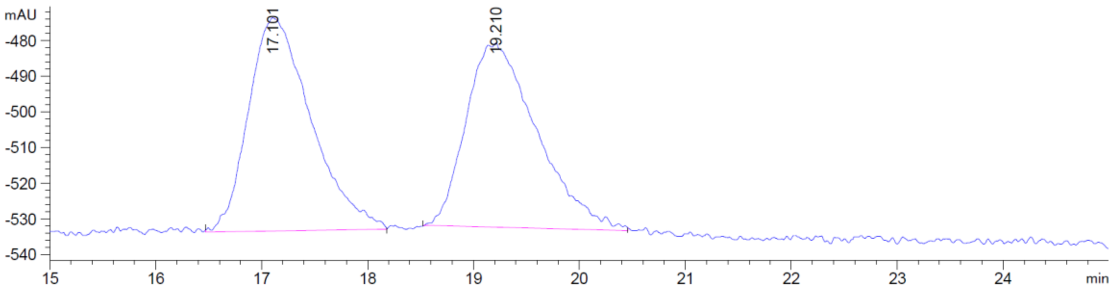

Peak#	Ret. Time	Area	Height	Area %
1	17.101	2457.06079	59.71291	50.5732
2	19.210	2401.36621	51.61246	49.4268
Total		4858.42700	111.32537	100

(R)-3i

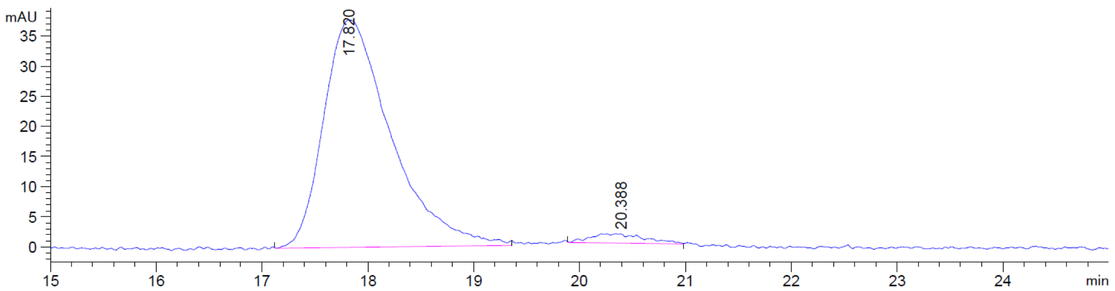

Peak#	Ret. Time	Area	Height	Area %
1	17.820	1666.47339	37.70516	96.7658
2	20.388	55.69886	1.54366	3.2342
Total		1722.17225	39.24882	100

(S)-3i

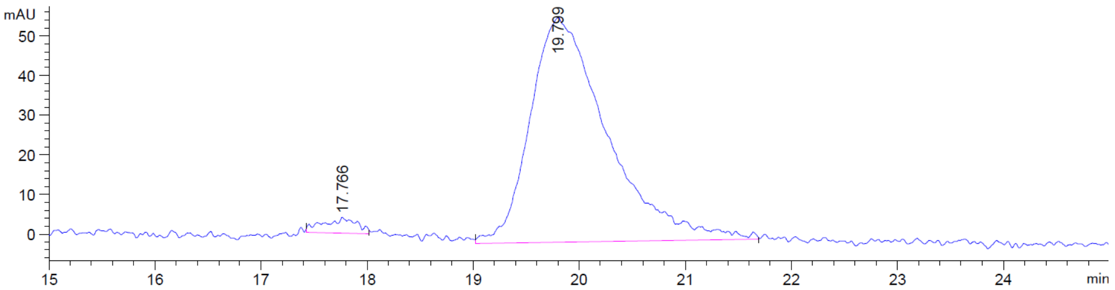

Peak#	Ret. Time	Area	Height	Area %
1	17.766	84.49088	4.00773	2.8634
2	19.799	2866.25366	56.6673	97.1366
Total		2950.74454	60.67503	100

Racemic 3j

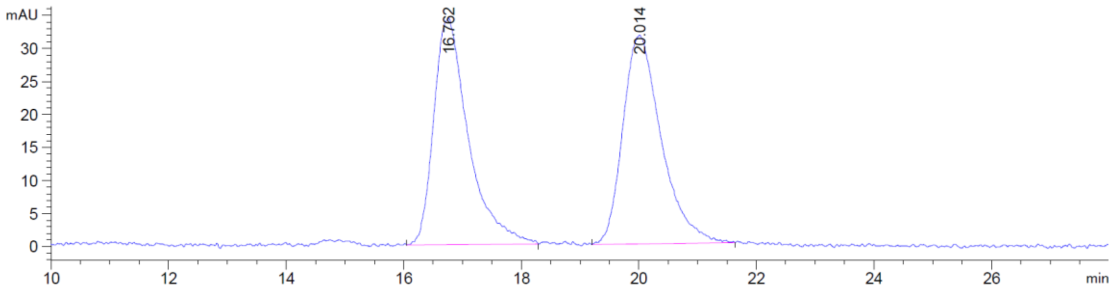

Peak#	Ret. Time	Area	Height	Area %
1	16.762	1405.58447	34.33652	49.4454
2	20.014	1437.11499	31.60749	50.5546
Total		2842.69946	65.94401	100

(R)-3j

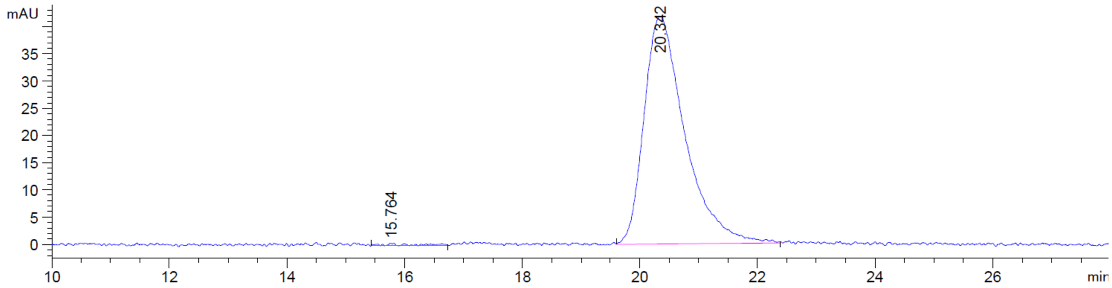

Peak#	Ret. Time	Area	Height	Area %
1	15.764	8.31346	3.63E-01	0.4093
2	20.342	2022.58142	41.77503	99.5907
Total		2030.89488	42.13817	100

(S)-3j

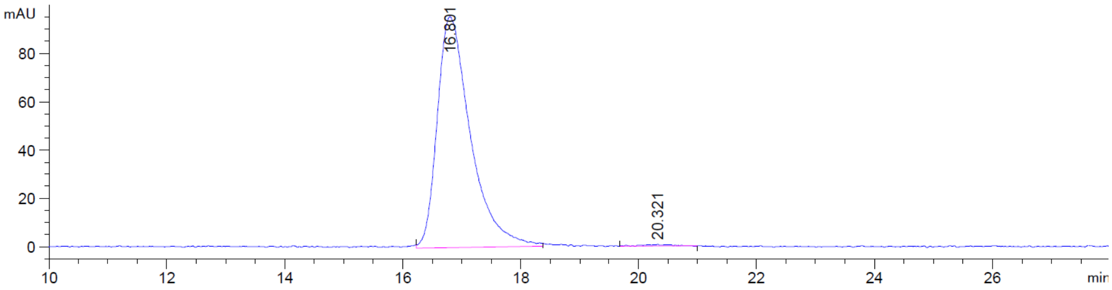

Peak#	Ret. Time	Area	Height	Area %
1	16.801	3747.42578	95.53815	99.4321
2	20.321	21.40235	0.68609	0.5679
Total		3768.82813	96.22425	100

Racemic 3k

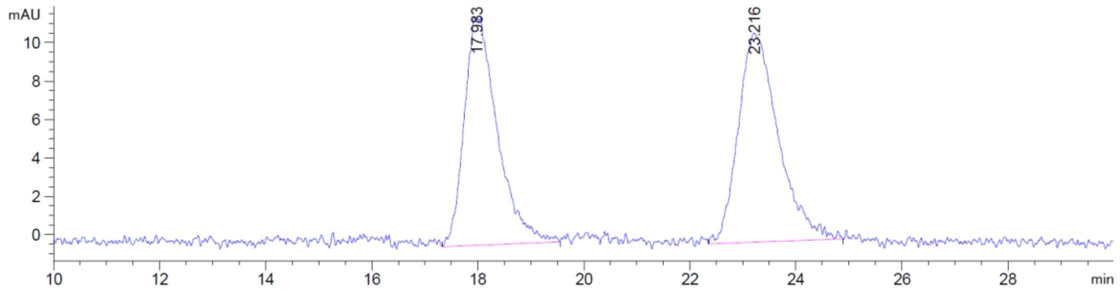

Peak#	Ret. Time	Area	Height	Area %
1	17.983	518.06964	11.79803	47.5418
2	23.216	571.64435	10.87944	52.4582
Total		1089.71399	22.67747	100

(R)-3k

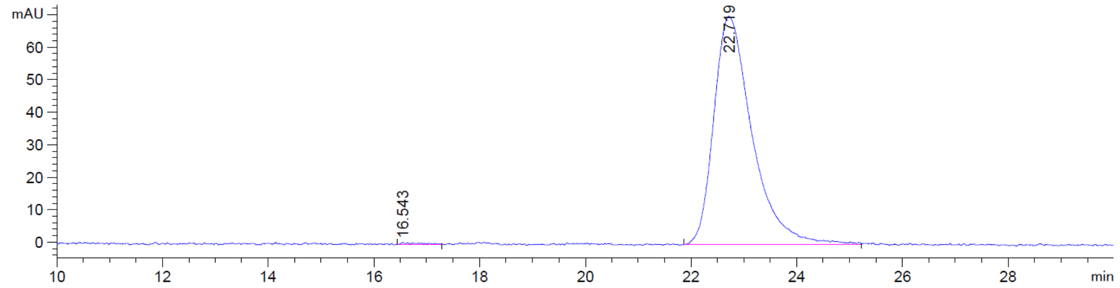

Peak#	Ret. Time	Area	Height	Area %
1	16.543	14.28822	6.11E-01	0.4030
2	22.719	3530.93335	70.18157	99.5970
Total		3545.22157	70.79281	100

(S)-3k

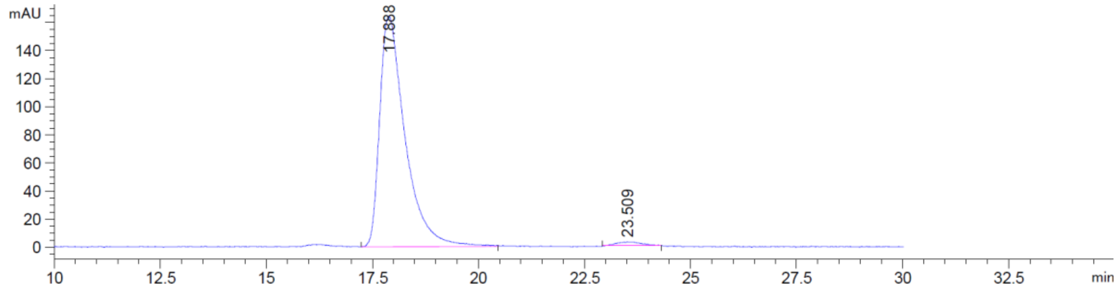

Peak#	Ret. Time	Area	Height	Area %
1	17.888	6840.56738	164.26079	98.5260
2	23.509	102.33601	2.68085	1.4740
Total		6942.9034	166.94164	100

Racemic 3l

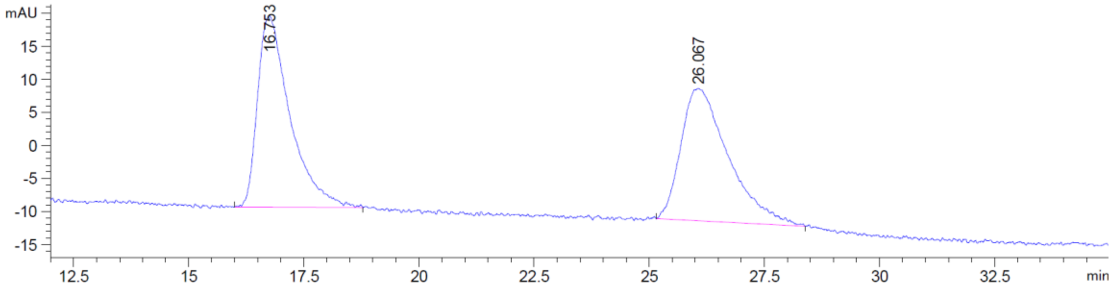

Peak#	Ret. Time	Area	Height	Area %
1	16.753	1419.74829	28.99684	49.6847
2	26.067	1437.76953	20.03554	50.3153
Total		2857.51782	49.03238	100

(R)-3l

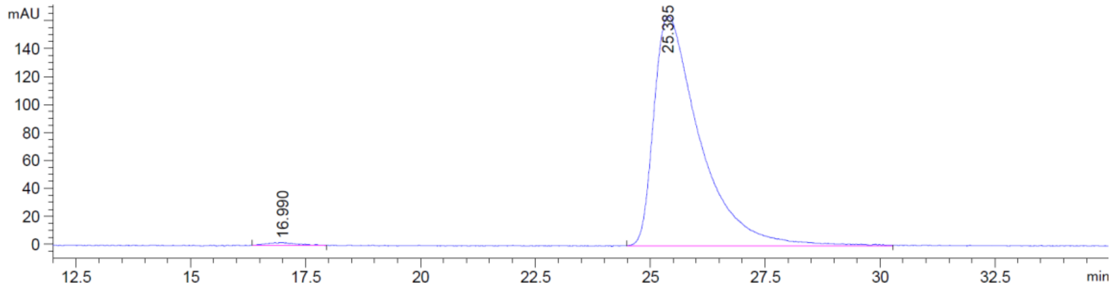

Peak#	Ret. Time	Area	Height	Area %
1	16.99	71.29169	1.81872	0.6174
2	25.385	1.15E+04	164.07285	99.3826
Total		1.15E+04	165.89157	100

(S)-3l

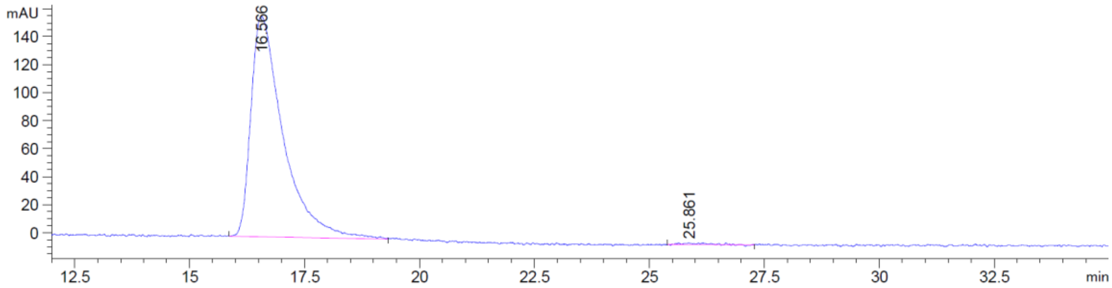

Peak#	Ret. Time	Area	Height	Area %
1	16.566	7662.3501	157.56442	99.342
2	25.861	50.7512	1.21058	0.658
Total		7713.1013	158.77501	100

Racemic 3m

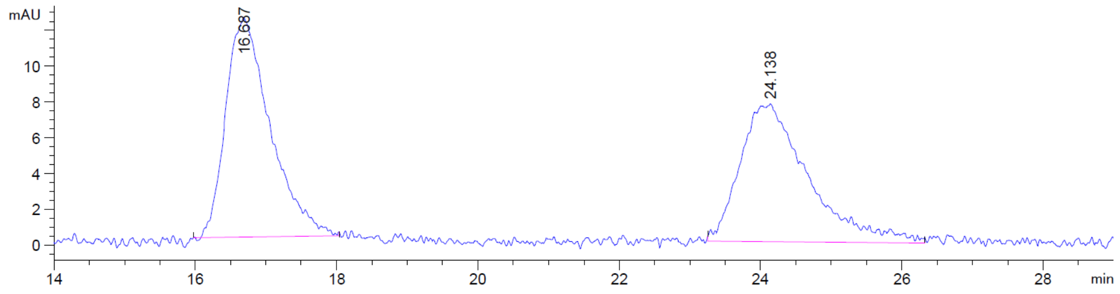

Peak#	Ret. Time	Area	Height	Area %
1	16.687	536.18365	12.24921	50.9733
2	24.138	515.70837	7.72048	49.0267
Total		1051.89203	19.96969	100

(R)-3m

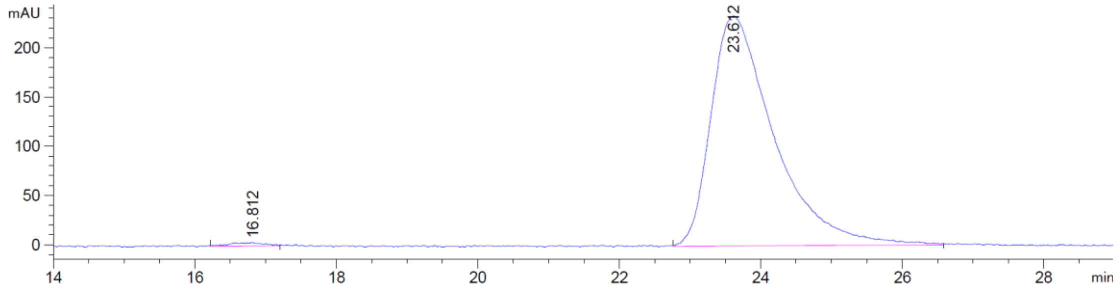

Peak#	Ret. Time	Area	Height	Area %
1	16.812	124.34901	3.49059	0.8543
2	23.612	1.44E+04	233.38474	99.1457
Total		1.46E+04	236.87532	100

(S)-3m

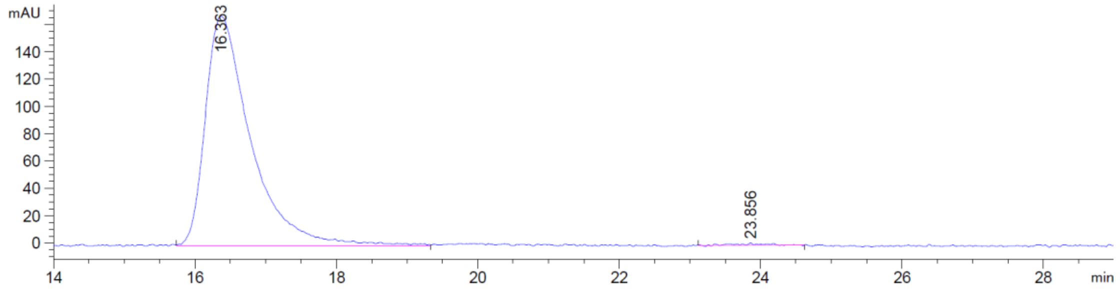

Peak#	Ret. Time	Area	Height	Area %
1	16.363	7498.44971	168.09459	99.5634
2	23.856	32.88212	1.63391	0.4366
Total		7531.33183	169.72849	100

Racemic 3n

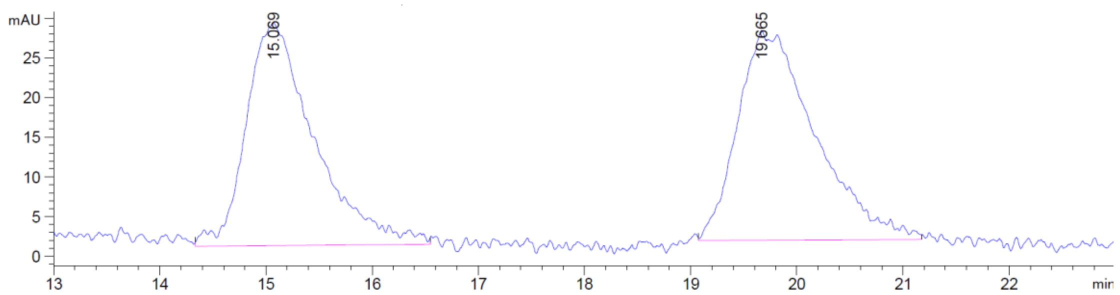

Peak#	Ret. Time	Area	Height	Area %
1	15.069	1247.3949	28.00523	48.2643
2	19.665	1337.11475	26.40146	51.7357
Total		2584.50964	54.40669	100

(R)-3n

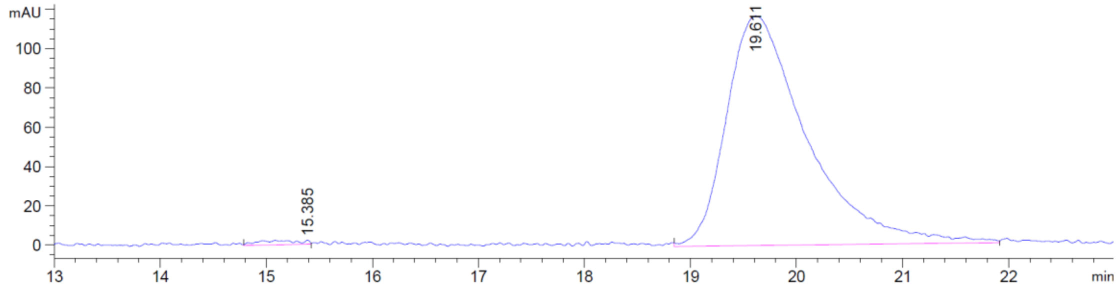

Peak#	Ret. Time	Area	Height	Area %
1	15.385	56.78538	1.94244	0.9106
2	19.611	6179.37939	117.02175	99.0894
Total		6236.16477	118.96419	100

(S)-3n

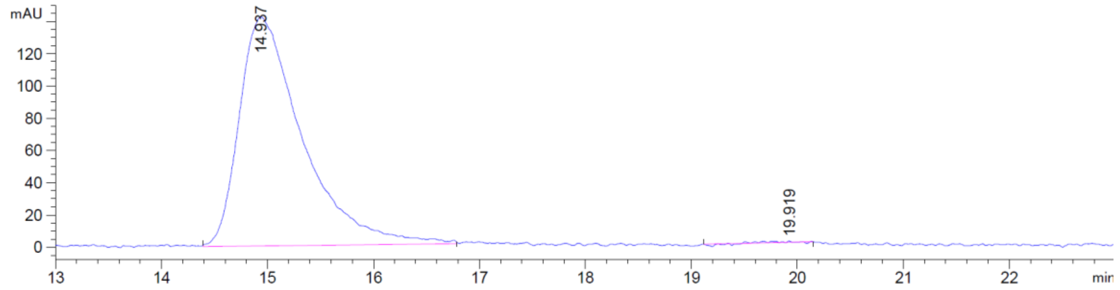

Peak#	Ret. Time	Area	Height	Area %
1	14.937	5881.52295	142.17091	99.7067
2	19.919	17.30142	0.933597	0.2933
Total		5898.82437	143.10451	100

Racemic 3o

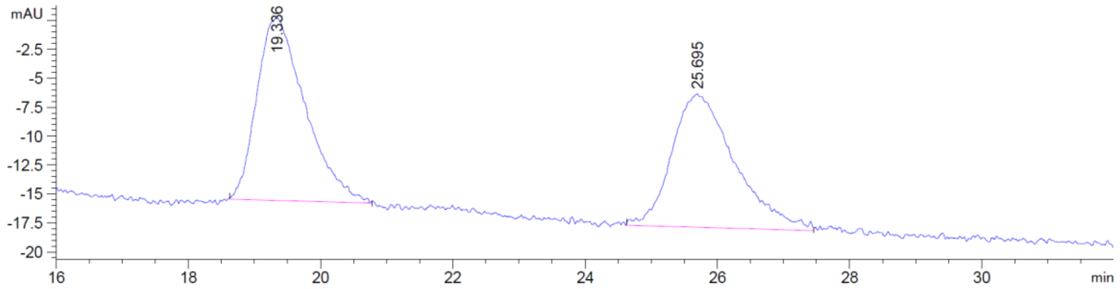

Peak#	Ret. Time	Area	Height	Area %
1	19.336	805.69727	15.87615	51.3572
2	25.695	763.11328	11.53148	48.6428
Total		1568.81055	27.40763	100

(R)-3o

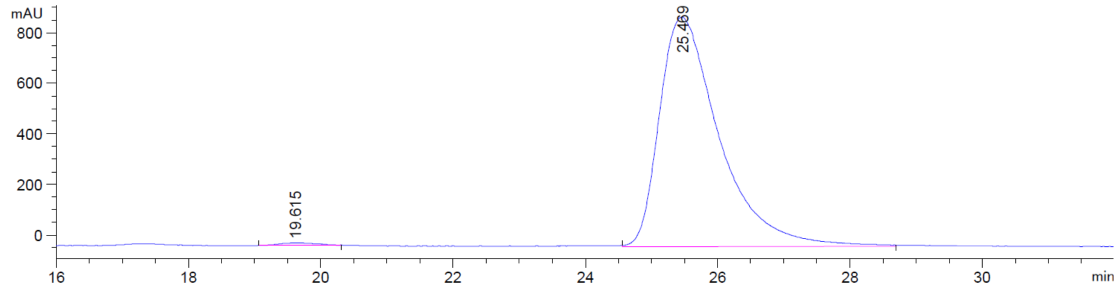

Peak#	Ret. Time	Area	Height	Area %
1	19.615	349.86359	9.28171	0.6072
2	25.469	5.73E+04	910.33600	99.3928
Total		5.76E+04	919.61771	100

(S)-3o

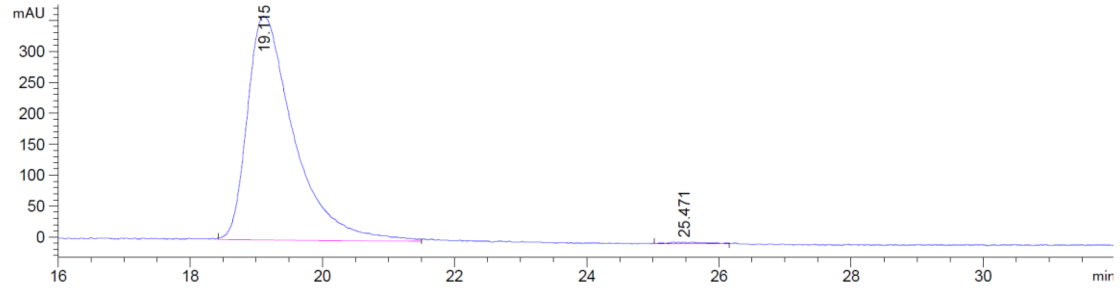

Peak#	Ret. Time	Area	Height	Area %
1	19.115	1.77E+04	361.78647	99.5911
2	25.471	72.60406	2.36389	0.4089
Total		1.78E+04	364.15035	100

Racemic 3p

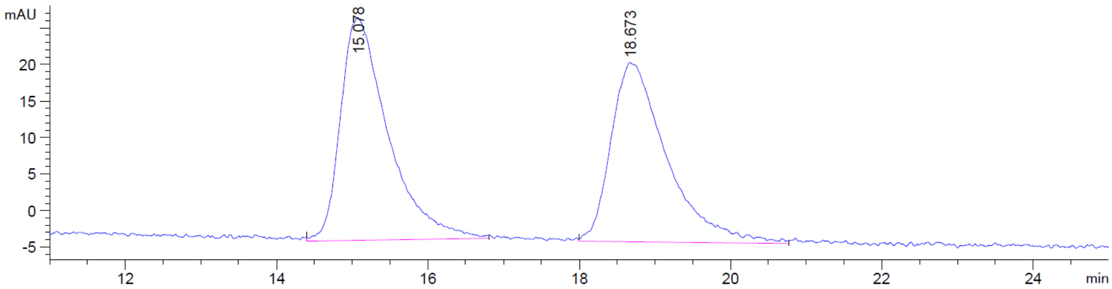

Peak#	Ret. Time	Area	Height	Area %
1	15.078	1326.47778	30.46709	51.3918
2	18.673	1254.63086	24.52537	48.6082
Total		2581.10864	54.99246	100

(R)-3p

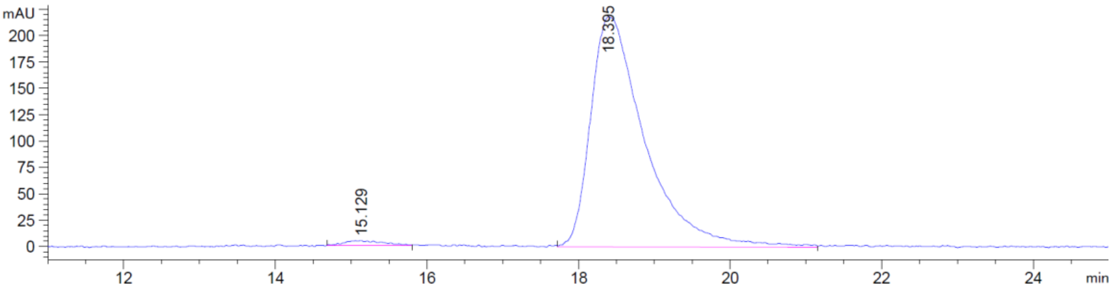

Peak#	Ret. Time	Area	Height	Area %
1	15.129	163.2025	4.71204	1.4738
2	18.395	1.09E+04	218.55911	98.5262
Total		1.11E+04	223.27115	100

(S)-3p

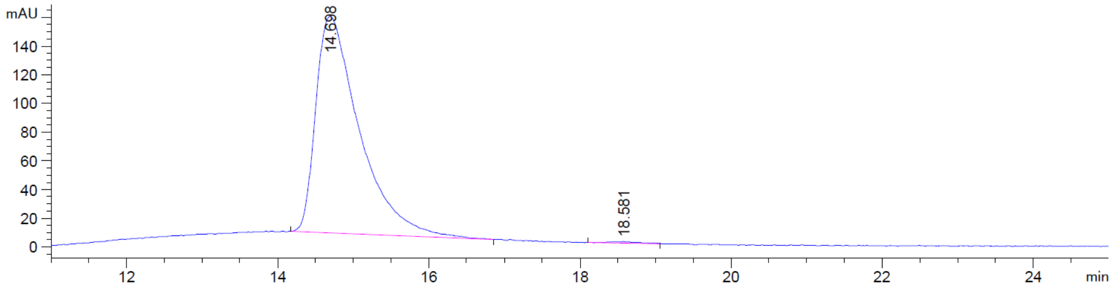

Peak#	Ret. Time	Area	Height	Area %
1	14.698	6061.97998	150.95914	99.5134
2	18.581	29.64137	1.06718	0.4866
Total		6091.62136	152.02632	100

Racemic 3q

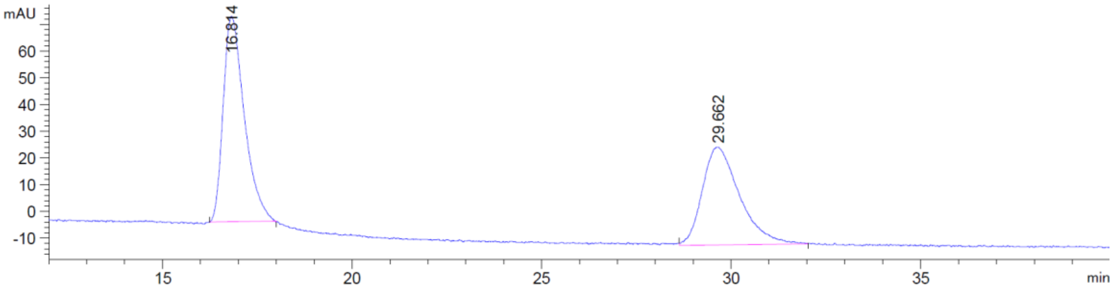

Peak#	Ret. Time	Area	Height	Area %
1	16.814	2978.90137	76.84653	54.2091
2	29.662	2516.30542	36.64914	45.7909
Total		5495.20679	113.49567	100

(R)-3q

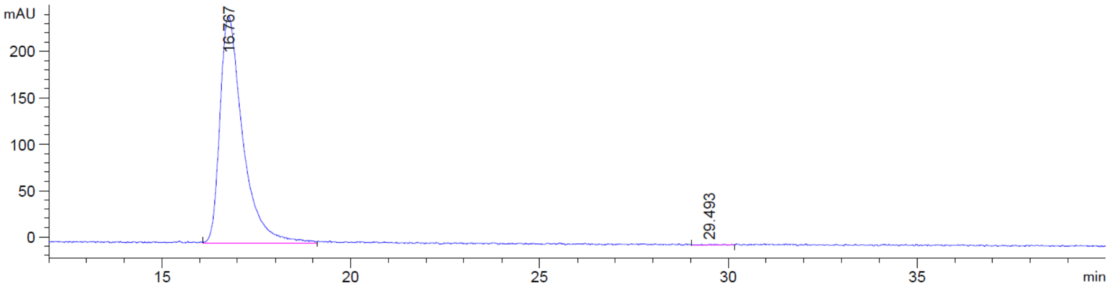

Peak#	Ret. Time	Area	Height	Area %
1	16.767	1.03E+04	2.44E+02	99.7895
2	29.493	21.62613	1.00367	0.2105
Total		1.03E+04	244.83743	100

(S)-3q

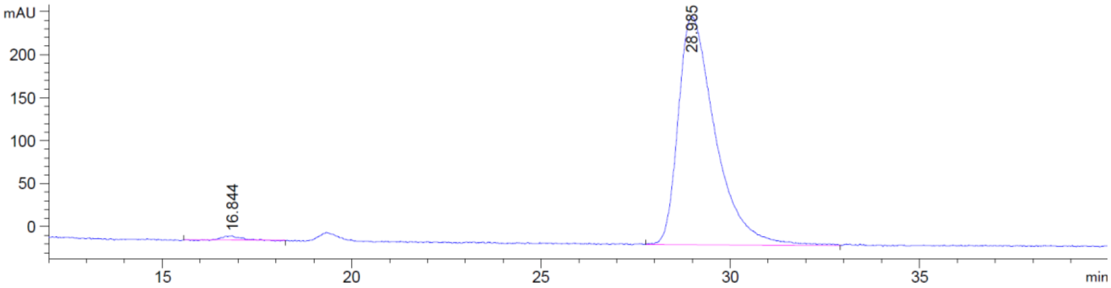

Peak#	Ret. Time	Area	Height	Area %
1	16.844	148.60652	4.71581	0.8164
2	28.985	1.81E+04	265.82199	99.1836
Total		1.82E+04	270.53781	100

Racemic 3r

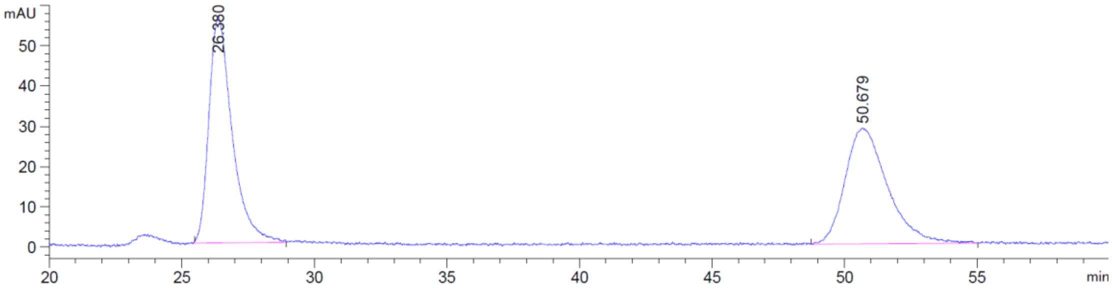

Peak#	Ret. Time	Area	Height	Area %
1	26.38	3413.69336	56.21083	51.8053
2	50.679	3175.77856	28.66201	48.1947
Total		6589.47192	84.87284	100

(R)-3r

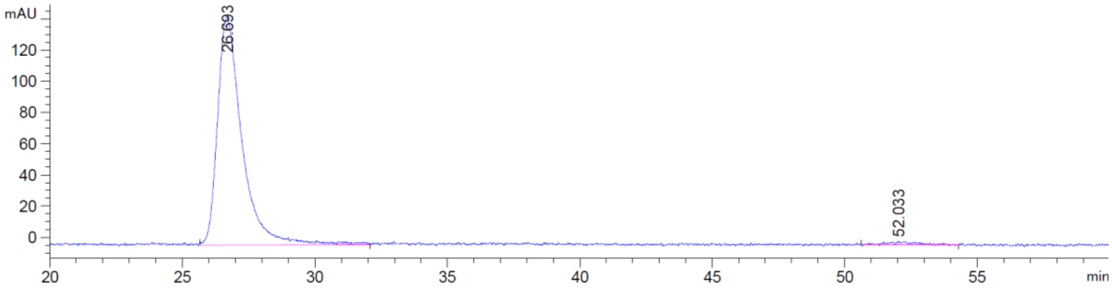

Peak#	Ret. Time	Area	Height	Area %
1	26.693	9397.35059	146.66017	98.7008
2	52.033	123.69892	2.04084	1.2992
Total		9521.04951	148.70102	100

(S)-3r

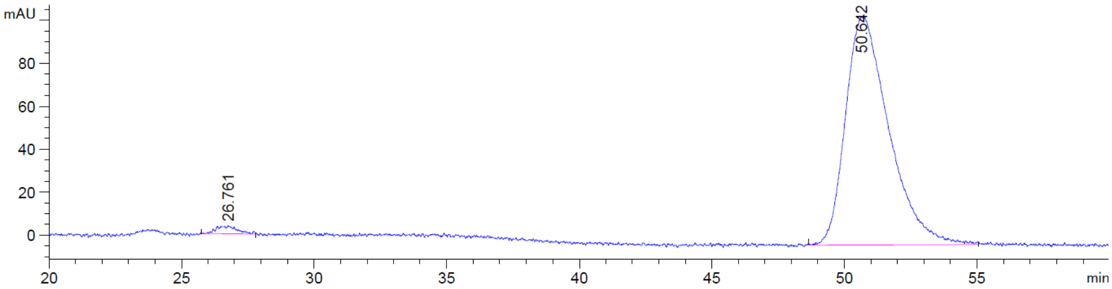

Peak#	Ret. Time	Area	Height	Area %
1	26.761	195.647	3.76826	1.5907
2	50.642	1.21E+04	106.45315	98.4093
Total		1.23E+04	110.22141	100

Racemic 3s

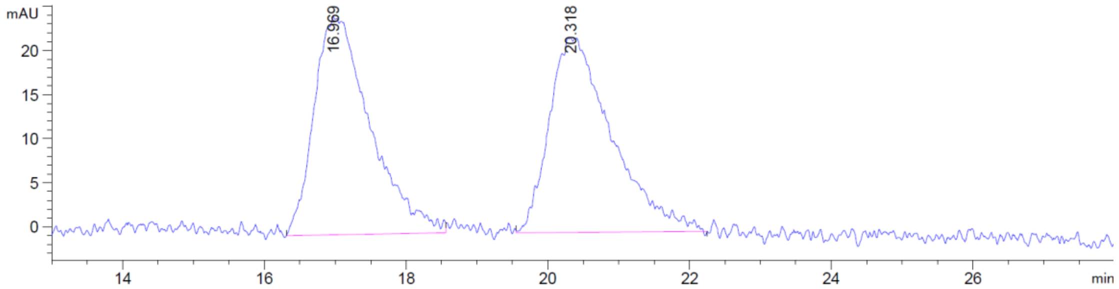

Peak#	Ret. Time	Area	Height	Area %
1	16.969	1367.39539	24.76033	50.122
2	20.318	1360.73669	22.09849	49.878
Total		2728.13208	46.85882	100

(R)-3s

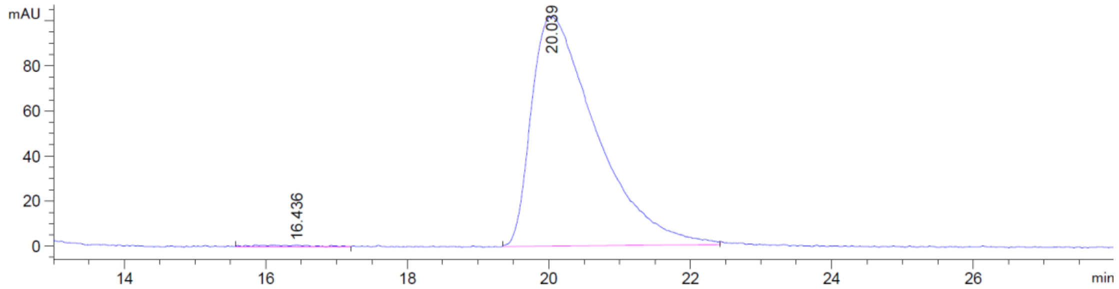

Peak#	Ret. Time	Area	Height	Area %
1	16.436	26.48434	6.05E-01	0.4067
2	20.039	6485.60303	101.8335	99.5933
Total		6512.08736	102.43892	100

(S)-3s

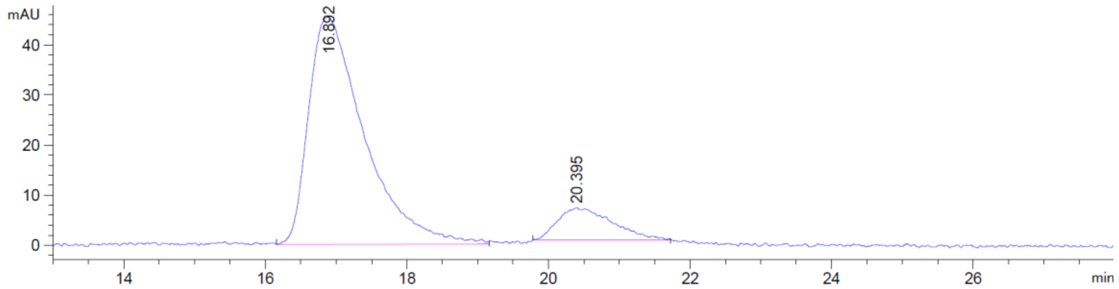

Peak#	Ret. Time	Area	Height	Area %
1	16.892	2476.60645	45.34282	87.5688
2	20.395	351.57571	6.52793	12.4312
Total		2828.18216	51.87075	100

Racemic 3t

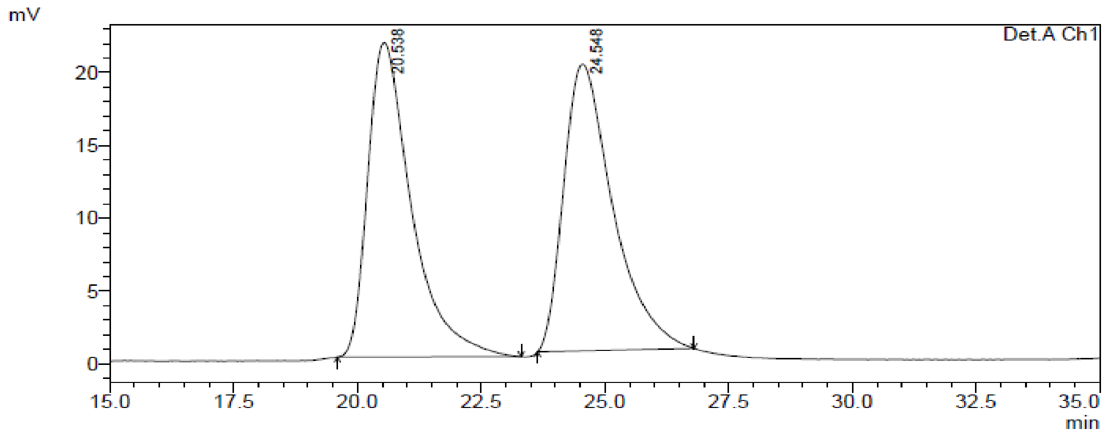

Peak#	Ret. Time	Area	Height	Area %
1	20.538	1327344	21588	49.327
2	24.548	1363576	19691	50.673
Total		2690920	41280	100

(R)-3t

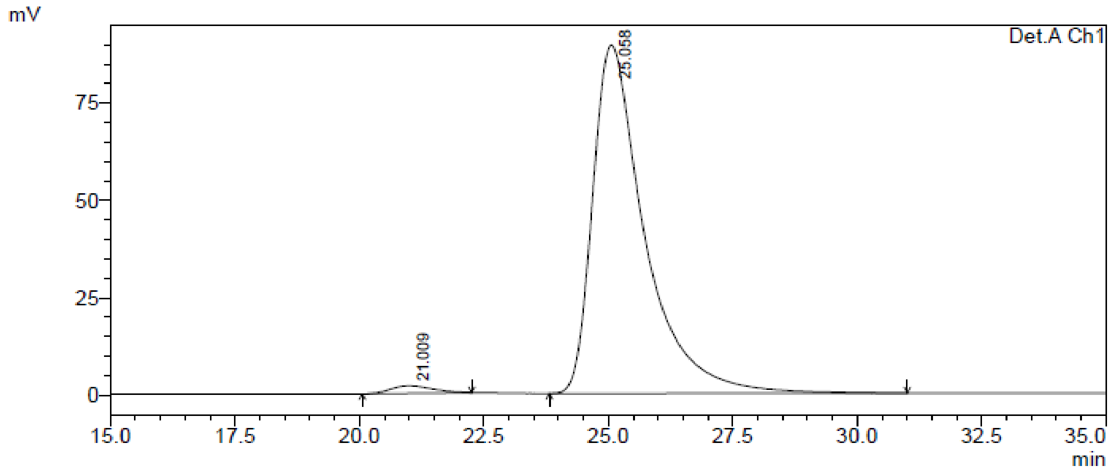

Peak#	Ret. Time	Area	Height	Area %
1	21.009	107169	1885	1.621
2	25.058	6502488	89511	98.379
Total		6609657	91396	100

Racemic 3u

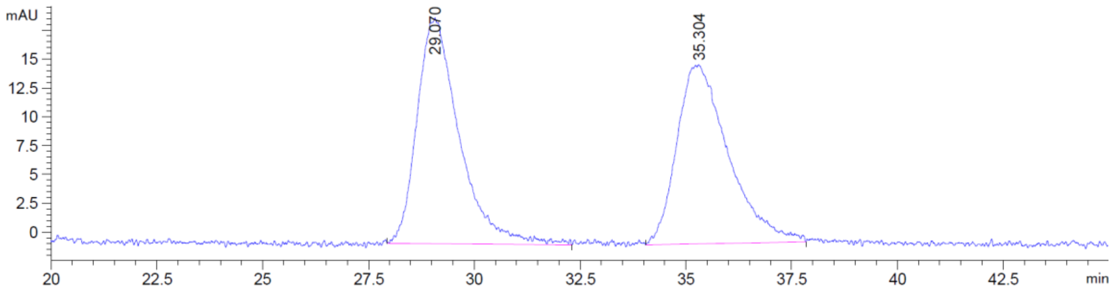

Peak#	Ret. Time	Area	Height	Area %
1	29.07	1358.50525	19.52679	50.8245
2	35.304	1314.43091	15.4625	49.1755
Total		5550.12915	75.05910	100

(R)-3u

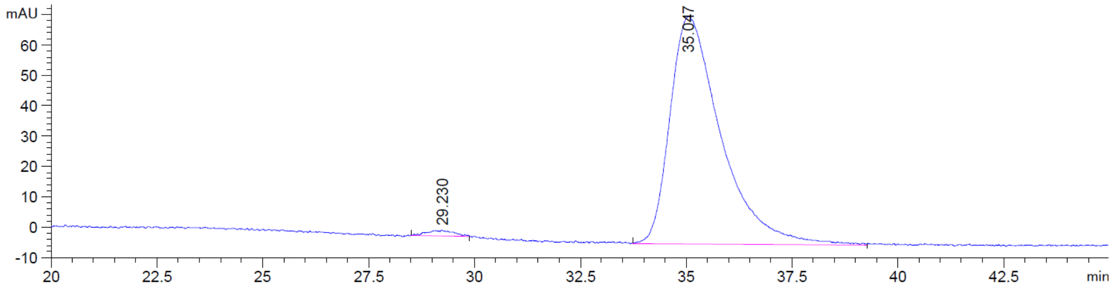

Peak#	Ret. Time	Area	Height	Area %
1	29.101	170.09424	3.94929	1.3331
2	35.073	1.26E+04	154.26450	98.6669
Total		1.28E+04	158.21379	100

(S)-3u

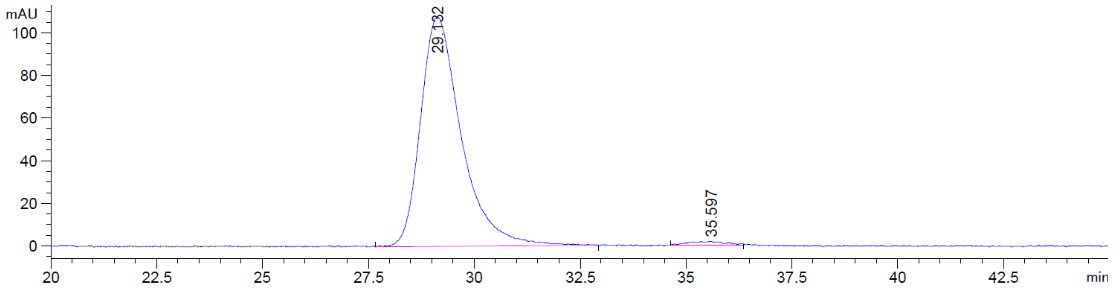

Peak#	Ret. Time	Area	Height	Area %
1	29.132	7232.5542	107.58244	98.6576
2	35.597	98.41095	1.69564	1.3424
Total		7330.96515	109.27807	100

Racemic 3v

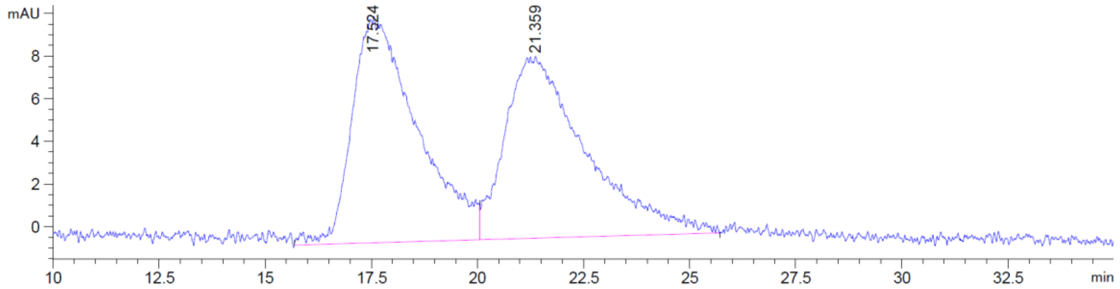

Peak#	Ret. Time	Area	Height	Area %
1	17.524	1133.71826	10.35238	50.0458
2	21.359	1131.64209	8.52675	49.9542
Total		2265.36035	18.87914	100

(R)-3v

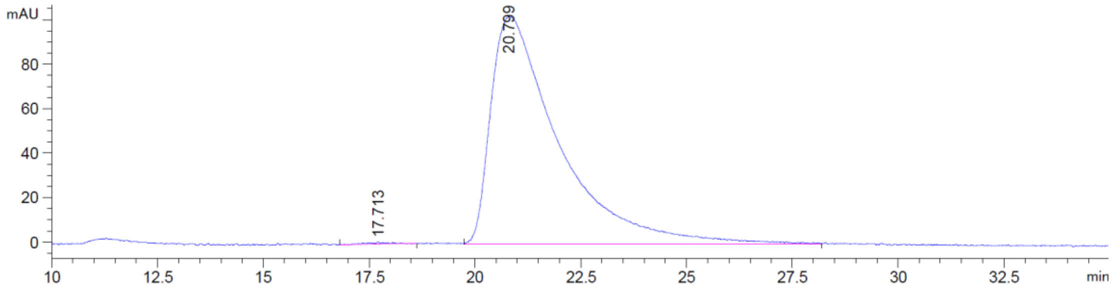

Peak#	Ret. Time	Area	Height	Area %
1	17.713	35.01977	0.942405	0.3031
2	20.799	1.15E+04	102.38928	99.6969
Total		1.16E+04	103.33169	100

(S)-3v

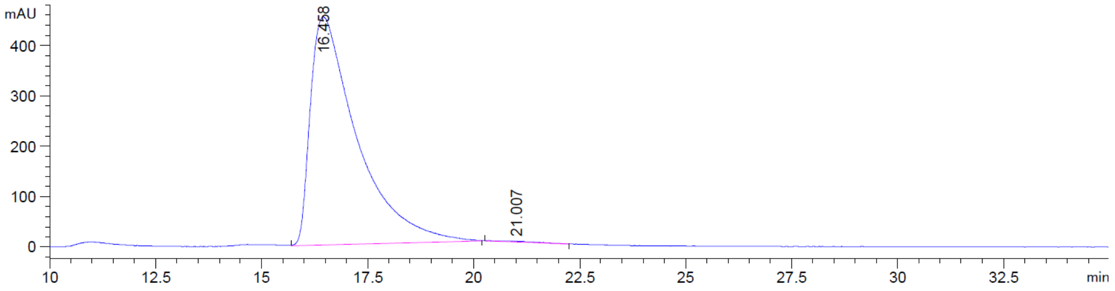

Peak#	Ret. Time	Area	Height	Area %
1	16.458	3.46E+04	452.81876	99.6736
2	21.007	113.28883	1.76258	0.3264
Total		3.47E+04	454.58134	100

Racemic 3w

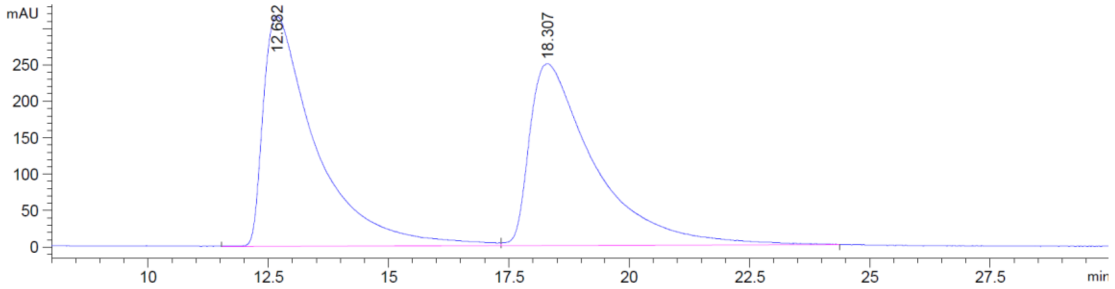

Peak#	Ret. Time	Area	Height	Area %
1	12.682	2.42E+04	315.68881	50.6898
2	18.307	2.35E+04	249.35506	49.3102
Total		4.77E+04	565.04387	100

(R)-3w

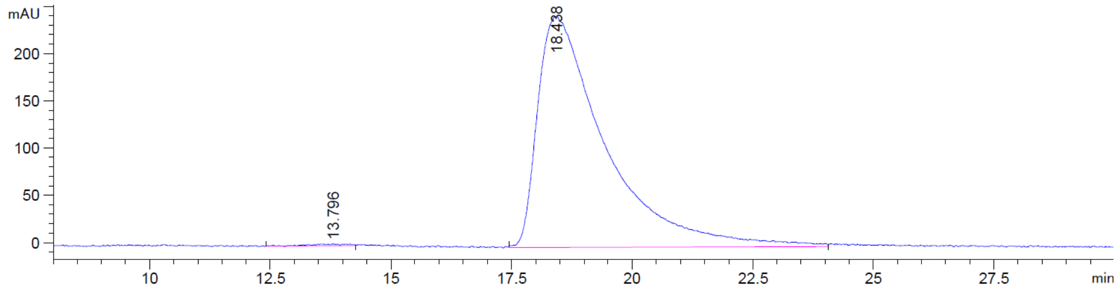

Peak#	Ret. Time	Area	Height	Area %
1	13.796	83.27142	1.9408	0.3471
2	18.438	2.39E+04	244.08846	99.6529
Total		2.40E+04	246.02925	100

(S)-3w

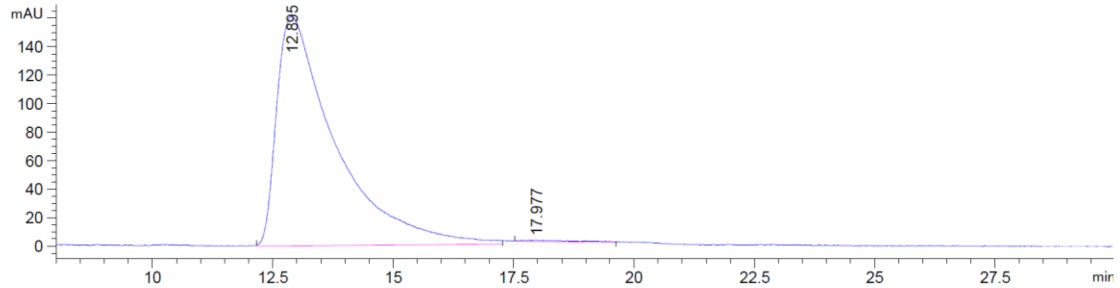

Peak#	Ret. Time	Area	Height	Area %
1	12.895	1.37E+04	161.30269	99.6664
2	17.977	45.7588	0.713493	0.3336
Total		1.37E+04	162.01618	100

Racemic 3x

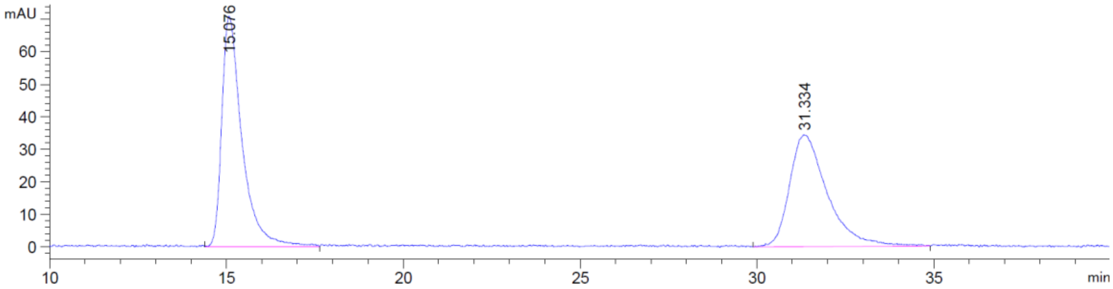

Peak#	Ret. Time	Area	Height	Area %
1	15.076	2808.46973	70.81115	52.3463
2	31.334	2556.70093	34.40984	47.6537
Total		5365.17065	105.22099	100

(R)-3x

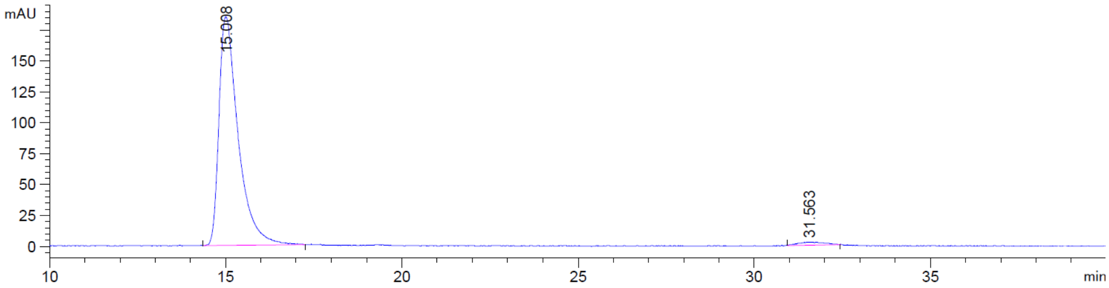

Peak#	Ret. Time	Area	Height	Area %
1	15.008	7014.73877	185.13393	98.293
2	31.563	121.82282	2.46342	1.707
Total		7136.56159	187.59735	100

(S)-3x

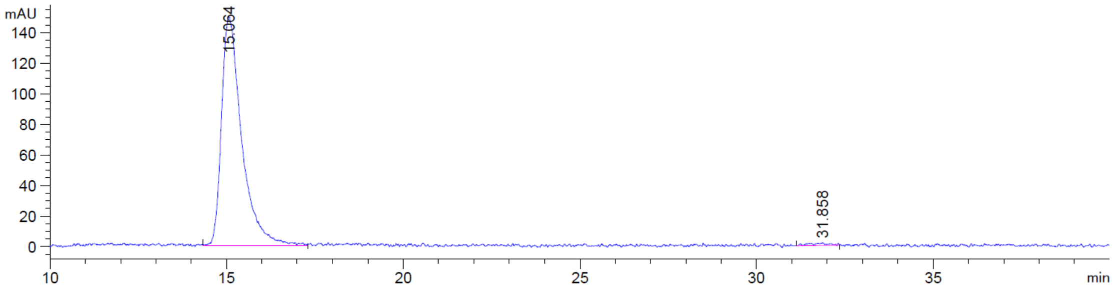

Peak#	Ret. Time	Area	Height	Area %
1	15.064	5896.73047	149.43005	98.9795
2	31.858	60.79724	60.79724	1.0205
Total		5957.52771	151.08756	100

Racemic 3y

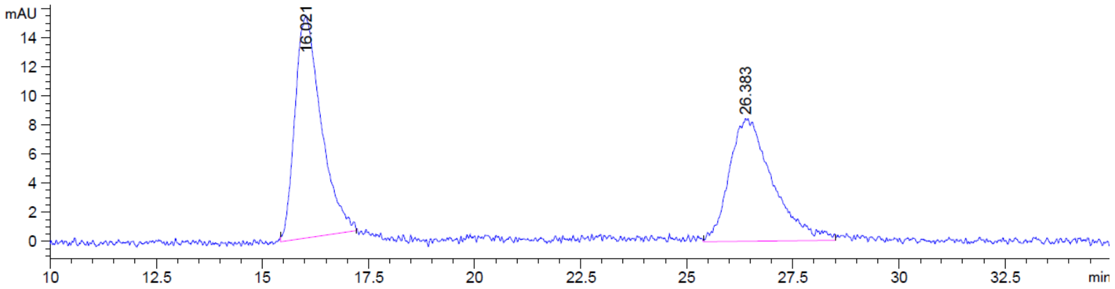

Peak#	Ret. Time	Area	Height	Area %
1	16.021	655.83466	15.21631	52.3536
2	26.383	596.86694	8.40443	47.6464
Total		1252.7016	23.62074	100

(R)-3y

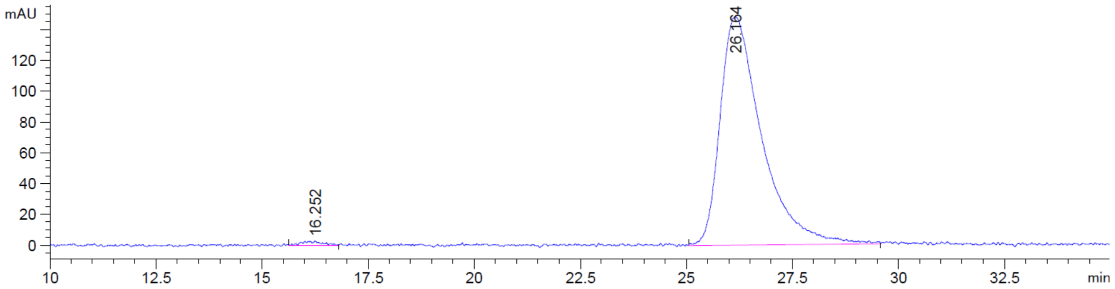

Peak#	Ret. Time	Area	Height	Area %
1	16.252	76.78291	2.62393	0.7525
2	26.164	1.01E+04	148.35654	99.2475
Total		1.02E+04	150.98047	100

(S)-3y

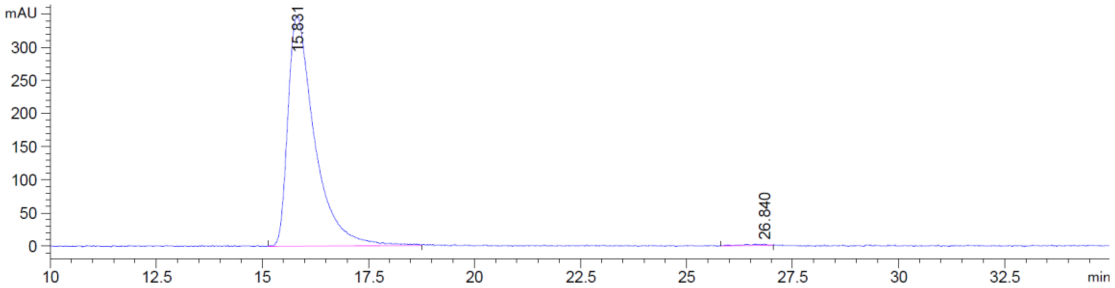

Peak#	Ret. Time	Area	Height	Area %
1	15.831	1.53E+04	347.84988	99.5461
2	26.840	69.8802	1.56174	0.4539
Total		1.54E+04	349.41162	100

Racemic 3z

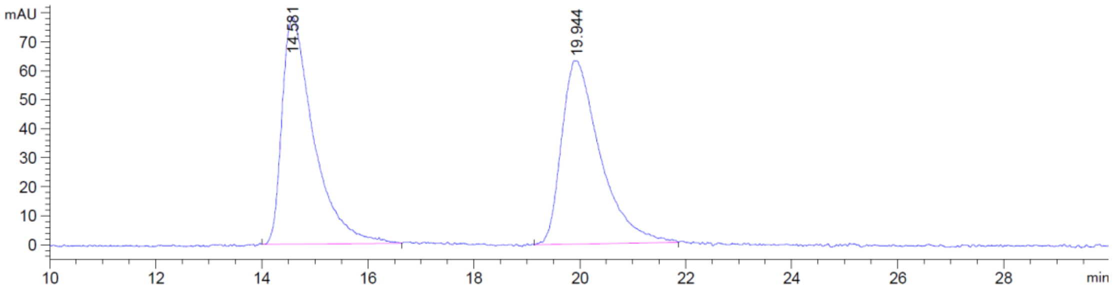

Peak#	Ret. Time	Area	Height	Area %
1	14.581	3245.05273	78.41568	50.3486
2	19.944	3200.11353	63.19697	49.6514
Total		6445.16626	141.61265	100

(R)-3z

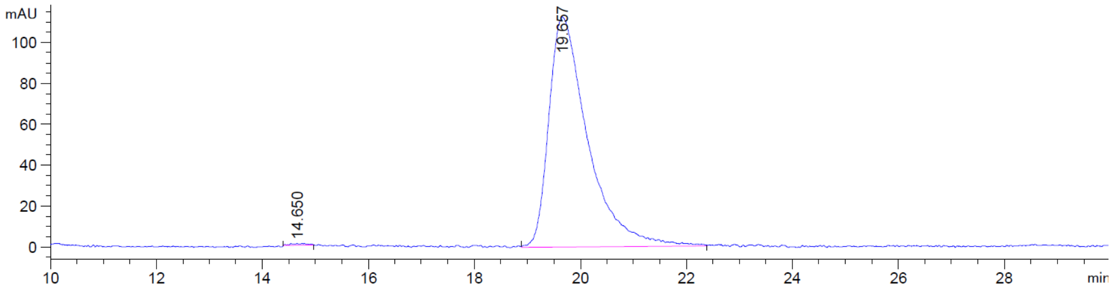

Peak#	Ret. Time	Area	Height	Area %
1	14.650	18.64397	8.34E-01	0.3237
2	19.657	5740.13525	112.64197	99.6763
Total		5758.77923	113.47624	100

(S)-3z

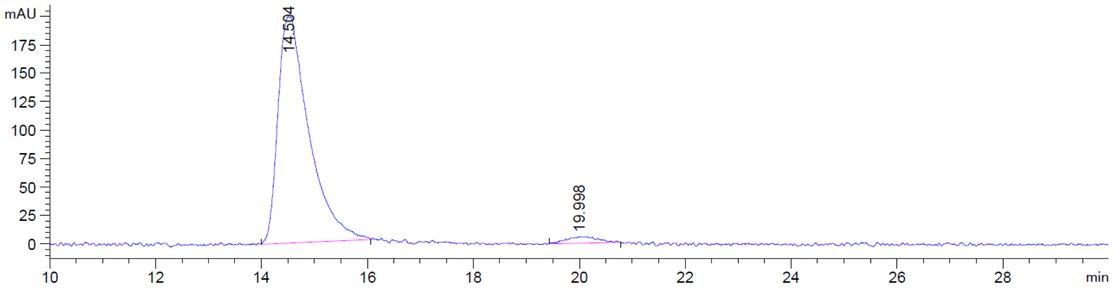

Peak#	Ret. Time	Area	Height	Area %
1	14.504	8067.06494	199.01576	97.2351
2	19.998	229.38701	5.6941	2.7649
Total		8296.45195	204.70986	100

Racemic 4

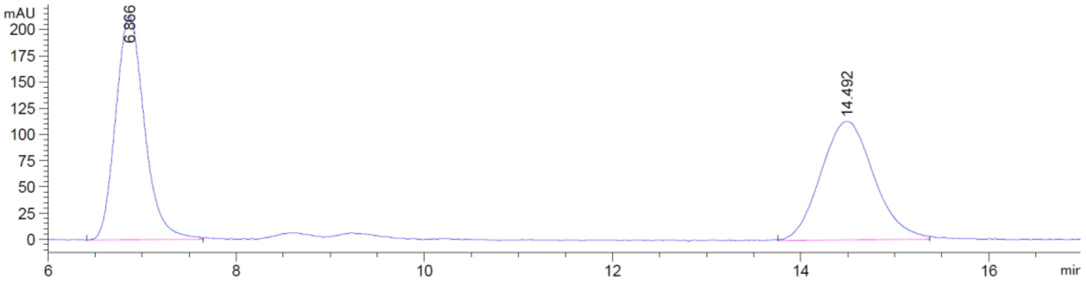

Peak#	Ret. Time	Area	Height	Area %
1	6.866	4546.5996	213.6537	50.571
2	14.492	4444.0405	113.1128	49.429
Total		8990.6401	326.7666	100

(R)-4

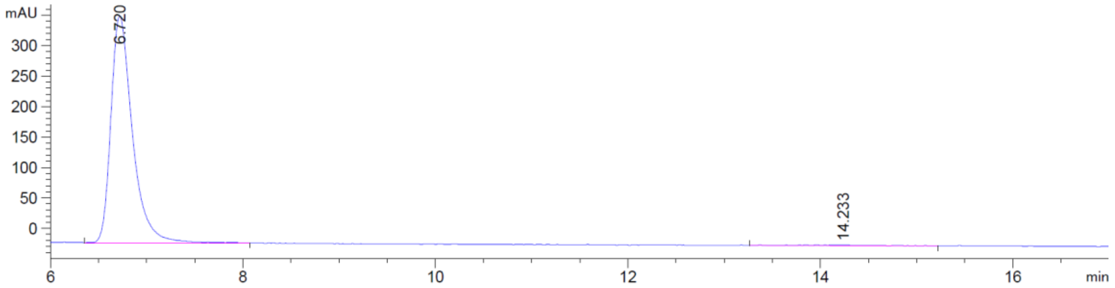

Peak#	Ret. Time	Area	Height	Area %
1	6.72	5801.51709	372.31171	98.9253
2	14.233	63.02462	1.16945	1.0747
Total		5864.54171	373.48115	100

Racemic 5

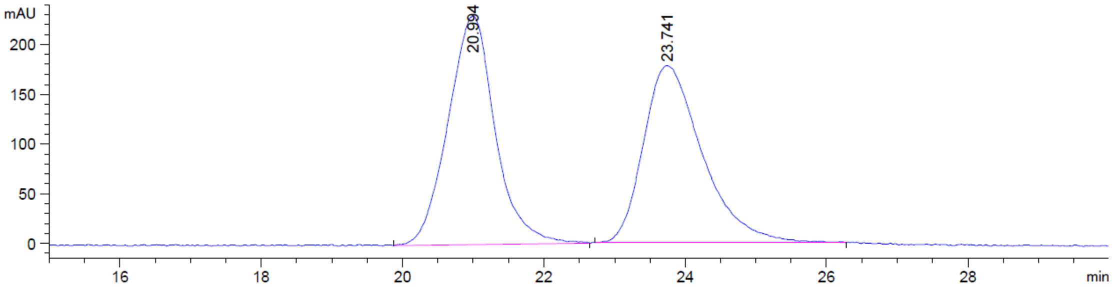

Peak#	Ret. Time	Area	Height	Area %
1	15.438	3797.4604	106.0592	48.434
2	16.46	4043.1047	96.5039	51.566
Total		7840.5651	202.5631	100

(R)-5

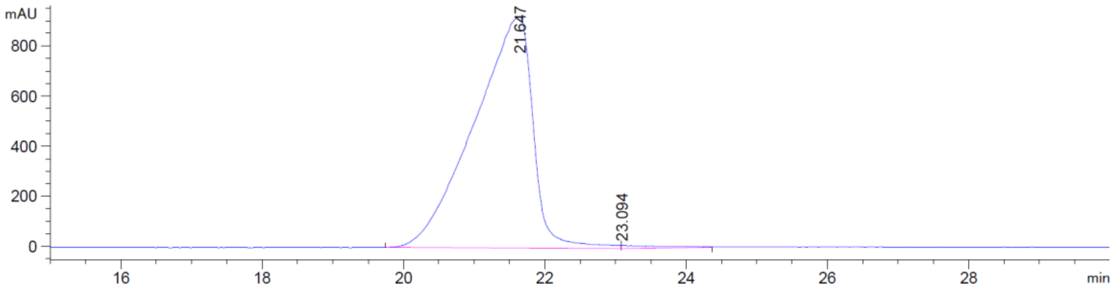

Peak#	Ret. Time	Area	Height	Area %
1	21.647	5.36E+04	922.81635	99.1397
2	23.094	465.11322	11.42434	0.8603
Total		5.41E+04	934.24068	100
